# Synthesis, Cytotoxicity Evaluation, and Molecular Dynamics of 4-Thiazolidinone Derivatives: A Potential Path to EGFR-Targeted Anticancer Therapy

**DOI:** 10.2174/0109298673400295250913044755

**Published:** 2025-10-10

**Authors:** Tomasz Szostek, Dagmara Otto-Ślusarczyk, Michał A. Dobrowolski, Maciej Wiśniewski, Piotr Roszkowski, Daniel Szulczyk

**Affiliations:** 1 Chair and Department of Biochemistry, Medical University of Warsaw, 02-097 Warsaw, Poland;; 2 Faculty of Chemistry, University of Warsaw, Pasteura 1, 02-093 Warsaw, Poland;; 3 Laboratory of Bioinformatics and Computational Genomics, Faculty of Mathematics and Information Science, Warsaw, Poland;; 4 Laboratory of Functional and Structural Genomics, Center of New Technologies, University of Warsaw, Warsaw, Poland

**Keywords:** 4-thiazolidinone, anticancer, cytotoxicity, selectivity index, breast adenocarcinoma, molecular docking

## Abstract

**Introduction:**

Heterocyclic compounds remain cornerstones of contemporary drug discovery because their ring-embedded heteroatoms confer adaptable electronics, conformational flexibility, and a broad spectrum of biological activities. The skeleton structure of 4-thiazolidinone is present in many cytotoxically active compounds and is often used in the design of new antitumor agents. This study aimed to synthesize, characterize, and evaluate the anticancer potential of fifteen new (2-imino-4-oxo-1,3-thiazolidin-5-yl)acetic acid derivatives.

**Methods:**

Compounds were synthesized using a consistent synthetic route involving a reaction between a thiourea derivative and maleic anhydride, which formed the thiazolidin-4-one ring through cyclization. The compounds were then categorized into three sets based on the attached heterocyclic rings (tryptamine, thiazole, and 1,2,4-triazole). The NMR and X-ray analysis followed the synthesis. Apoptotic effects, cell cycle arrest, IL-6 suppression, docking, and dynamics simulations were conducted. Preliminary cytotoxic activity was tested on metastatic colorectal cancer (SW620) and human breast adenocarcinoma (MDA-MB-231) cell lines using the MTT assay.

**Results:**

Compounds **5**, **6**, and **7** demonstrated notable selectivity indexes (4.73, 2.42, 4.16, respectively) and were further investigated for their mechanisms of action, revealing pro-apoptotic properties and the ability to induce cell cycle arrest. Additionally, compound **5** inhibited IL-6 secretion by 76%. *In silico* studies revealed the formation of an energetically stable complex between compound **5** and the EGFR crystal structure (min/max binding affinities of -9.4|-8.0 kcal/mol, compared to the -7.71 kcal/mol for the native ligand).

**Discussion:**

This preliminary study provides compelling data on synthesized derivatives, but more advanced testing is needed to assess their therapeutic value fully. Compared with earlier reports on related thiazolidinone scaffolds, the present derivatives exhibit improved potency, clearer selectivity, and mechanistic features consistent with EGFR inhibition and cytokine modulation.

**Conclusion:**

These findings validate (2-imino-4-oxo-1,3-thiazolidin-5-yl)acetic acid as a privileged core for cytotoxic lead generation and indicate that strategic substitution with either a tryptamine moiety (compound **5**) or a 1,2,4-triazole ring (compound **7**) is particularly advantageous. These compounds are promising EGFR-targeting anticancer candidates, warranting further investigation.

## 1 INTRODUCTION

Regardless of many recent discoveries in the field of cancer treatment, its global epidemic remains one of the biggest challenges to the healthcare system. Despite promising data, as reported by the American Cancer Society, the total mortality rate associated with cancer in the United States decreased by 27% from 1991 to 2016 [1]. However, this phenomenon concerns only highly developed countries. According to Mattiuzzi and Lippi, global cancer frequency and mortality increase every year and are expected to maintain an incremental trend [2]. Moreover, currently used cancer treatments and newly discovered candidates for potential replacement are often highly toxic to normal human cells. This indicates that some cancer therapies, even if effective, cause dangerous adverse effects and reduce patients’ quality of life [3]. Therefore, the development of new anticancer compounds is of critical importance, particularly those distinguished by high selectivity.

The group of 4-Thiazolidinones has been proven to be particularly biologically active scaffolds. Their wide spectrum of action includes anticancer, antiprotozoal, anti-inflammatory, anticonvulsant, and antimicrobial activity [4]. Currently, a few medicines containing a 4-thiazolidinone structure are available on the worldwide pharmaceutical market with particular emphasis on their use in diabetes treatment. Glitazones, including Pioglitazone (Fig. **1**) [5] and Rosiglitazone [6], are considered type 2 diabetes medicines, and Epalrestat was approved in Japan for the treatment of diabetic peripheral neuropathy [7]. Other therapeutic areas using medicines containing 4-thiazolidinones derivatives as active substances include Diuretics (Etozoline) [8], anticonvulsants (Ralitoline) [9], and, most recently, from 2021, Ponesimod (Ponvory), which was approved by the FDA for the treatment of multiple sclerosis and psoriasis [10].

The skeleton structure of 4-thiazolidinone is present in many cytotoxically active compounds and is often used in the design of new antitumor agents. Türe *et al*. synthesized a series of new 4-thiazolidinone derivatives hybridized with Imatinib and investigated their cytotoxic properties. Three of the obtained hybrids demonstrated significant antiproliferative activity, further confirmed through apoptosis and cell cycle arrest tests. The most potent compound showed an IC_50_ of 4.86 ± 0.73 μM and 3.52 ± 0.91 μM for 24 and 48 hours, respectively, against K562 cells [11]. Szychowski *et al.* reported stimulation of ROS production in the squamous carcinoma cell line (SCC-15) after treatment with new 4-thiazolidinone derivatives (Les-2194, Les-3377, Les-3640) [12].

Particularly interesting are derivatives of (2-Imino-4-oxo-1,3-thiazolidin-5-yl)acetic acid, which were reported as potential antitumor agents by Amin *et al.* (2023). The compound was tested for antiproliferative activity with different scaffolds, such as thiosemicarbazides, and showed results of GI% = 11.9 and GI% = 12.9 against renal (UO-31) and breast (MCF7) cancer cell lines, respectively. Researchers concluded that preliminary anticancer studies suggested a better potential for (2-imino-4-oxo-1,3-thiazolidin-5-yl)acetic acid over thiosemicarbazides in this study [13]. Nossier *et al*. synthesized four new derivatives of 4-oxo-3-phenylthiazolidin-5-yl-acetic acid and evaluated their *in vitro* cytotoxic activity. In the cell viability test, the HEPG-2 (hepatoblastoma), PC-3 (prostatic adenocarcinoma), and A549 (non-small cell lung cancer) cell lines were treated with the compounds at a concentration of 100 μg/ml. Two synthesized compounds exhibited significant cytotoxic activity compared to the reference drug, doxorubicin. For the HEPG-2 cell line, the compounds showed viability percentages of 97 ± 1.10% and 92.9 ± 1.62%, with IC_50_ values of 3.43 ± 0.32 μM and 1.5 ± 0.18 μM, respectively. These compounds were subsequently subjected to molecular docking studies, which revealed an inhibitory effect against the GSTP1 enzyme [14].

The most recent research investigating the antitumor activity of novel (2-imino-4-oxo-1,3-thiazolidin-5-yl)acetic acid derivatives comes from 2024. They were hybridized with thymol and targeted against proviral integration sites for Moloney-murine leukemia (PIM) virus kinases. Researchers found that compounds exhibited inhibitory properties toward enzymes important for cancer metabolism and inflammation, including 5-LOX, COX-2, and PIM [15].

Moreover, 4-thiazolidinones were chosen because, unlike many frontline cytostatics that are virtually insoluble in water (*e.g.*, paclitaxel <0.01 mg/ml and < 60% oral bioavailability) [16], recent profiling shows most thiazolidinone-bearing leads dissolve in phosphate-buffered saline at ≥0.2 mg/ml and satisfy Lipinski/Veber rules, giving them a markedly better predicted biopharmaceutical profile [17].

The aim of the study was to obtain new biologically active compounds. To maximize the probability of achieving the desired effect, a strategic approach was necessary during the design phase of the synthesis. As a solution, characteristic heterocyclic structures were selected for inclusion in the new (2-imino-4-oxo-1,3-thiazolidin-5-yl)acetic acid derivatives scaffolds, giving small molecule hybrids. Hybridization of molecules with well-documented activity has been proven to increase the probability of obtaining more cytotoxic, selective, multitargeting agents [18]. The choice of specific heterocycles in our research was based on recent scientific reports showing their potential for improving cytotoxic activity.

Heterocyclic compounds remain cornerstones of contemporary drug discovery because their ring-embedded heteroatoms confer adaptable electronics, conformational flexibility, and a broad spectrum of biological activities. Recent examples illustrate this versatility: bis-Schiff bases bearing a thiobarbiturate nucleus have emerged as potent α-glucosidase inhibitors, validated by docking and DFT analyses [19], and closely related thiobarbiturate derivatives display dual urease-inhibitory and antioxidant properties [20]. Benzimidazole frameworks continue to yield high-value leads, ranging from submicromolar prolyl-oligopeptidase antagonists [21] to neuroprotective analogues that target enzymes implicated in neurodegeneration [22]. Hydrazone-Schiff benzimidazoles likewise inhibit urease and perform robustly in molecular dynamics simulations [23], while oxadiazole-flurbiprofen hybrids extend anti-inflammatory chemistry into the anticancer arena [24]. Quantum-chemical studies further clarify how heteroatom substitution modulates charge distribution and ligand-target recognition in these motifs [25]. Collectively, such findings highlight the strategic value of heterocycles and rationalize our focus on 4-thiazolidinone hybrids as a fertile platform for anticancer lead optimization.

Tryptamine derivatives are considered mainly as 5-hydroxytryptamine receptor agonists. However, recent studies report that they can also possess cytotoxic activity. Salikov *et al*., screened newly synthesized tryptamine analogs for antiproliferative activity against HEK293 (embryonic kidney), HEPG-2 (hepatoblastoma), and Jurkat (T lymphocyte) cell lines. The best candidate demonstrated an IC_50_ value of 4.3 ± 0.05 μM against the HEPG-2 cell line. Further confirmation of cytotoxic activity was obtained through Annexin V/PI staining, which indicated the presence of cells in apoptotic and necrotic states [26]. In another similar study, tryptamine derivatives exhibited even lower IC_50_ values. For example, the most active compound showed an IC_50_ value of 0.0015 μM against IGROV1 (human ovarian carcinoma) cells [27]. 1,2,4-Triazole and its derivatives possess a broad spectrum of biological activity including antifungal, antibacterial, and anticancer effects [28]. One of the most successful compounds containing 1,2,4-triazole, letrozole, has been approved for clinical tumor treatment [29]. *Guan et al.* reported a series of 1,2,4-triazoles hybridized with other N-heterocycles that showed promising cytotoxic activity through mechanisms of targeting VEGFR, TDO inhibition, microtubule inhibition, and others. Notably, the study mentions a hybrid of 1,2,3-triazole with 2,4-thiazolidinedione that exhibited higher cytotoxic activity against MCF-7 (human breast cancer) cells compared to the reference drug cisplatin [30]. The cytotoxic activity of thiazole derivatives is also well-known to scientists, and they are often used in designing new scaffolds with the potential of having antitumor activity [31]. Recent studies on thiazoles linked with other N-heterocycles, specifically thiosemicarbazones, have reported obtaining potentially cytotoxic active hybrids. After preliminary tests against HL-60 (human leukemia) cells, the best result of IC_50_ value was 3.67 ± 0.60 μM [32].

Thus, based on the extensive experience of our research team, as evidenced by the successful discovery of new biologically active compounds in previous studies [33-36], we propose a well-justified strategy for designing novel small-molecule hybrids. This approach focuses on aligning the highly active central structure of (2-imino-4-oxo-1,3-thiazolidin-5-yl)acetic acid with tryptamine, 1,2,4-triazole, and thiazole rings as active substituents. This strategy holds significant promise for the development of new potential antitumor agents, highlighting the critical importance of these molecular frameworks in enhancing both cytotoxic efficacy and selectivity.

## 2 MATERIALS AND METHODS

### 2.1 Chemistry

#### 2.1.1 Chemicals and Reagents

Chloroform, methanol, and acetone were obtained from Sigma Aldrich (Saint Louis, MO, USA). Tryptamine (98%) was sourced from Acros Organics (Geel, Belgium), while 2-aminothiazole (≥99%) and 3-amino-1,2,4-triazole (≥98.5%) were purchased from Sigma Aldrich (Saint Louis, MO, USA). All other reagents used were of analytical grade and applied without additional purification. Nuclear magnetic resonance (NMR) spectra were acquired using a Bruker AVANCE spectrometer (Bruker, Karlsruhe, Germany) operating at 300 or 500 MHz for ^1^H NMR, and at 75 or 125 MHz for ^13^C NMR. The spectra were recorded in CDCl_3_ or a CDCl_3_/CD_3_OD mixture (9:1), and chemical shifts (δ) are reported in ppm relative to tetramethylsilane (TMS). Electrospray ionization (ESI) mass spectra were measured on a high-resolution LCT Micromass TOF instrument (Micromass UK Limited, Manchester, UK). Melting points were determined using a KSP1D Melting Point Meter (A. Krüss Optronic, Hamburg, Germany) and are uncorrected. Thin-layer chromatography (TLC) was performed on silica gel plates (Merck Kiesegel GF254, Merck, Darmstadt, Germany) and visualized under UV light or by exposure to iodine vapors. Column chromatography was carried out at atmospheric pressure using Silica Gel 60 (230-400 mesh, Merck, Darmstadt, Germany), with a chloroform/methanol mixture (0-10%) as the mobile phase.

#### 2.1.2 General Procedure for the Synthesis of the Compounds

A solution containing the thiourea derivative (146 mg, 1 mmol) and maleic anhydride (98 mg, 1 mmol) in acetone (15 mL) was stirred and heated under reflux for 15 hours. Upon completion, the solvent was removed under reduced pressure. The resulting residue was then refluxed with a small volume of a benzene/water mixture to eliminate any residual anhydride. The organic phase was separated, washed with water (30 mL), and dried over anhydrous sodium sulfate (Na_2_SO_4_). Following solvent evaporation under reduced pressure, the crude product was purified *via* column chromatography on silica gel using a chloroform/methanol gradient (0-10% MeOH) as the eluent. The purified compound was subsequently recrystallized from a 1:1 mixture of water and absolute ethanol.

##### 2.1.2.1 [(2Z,5R)-2-(3-chloro-4-fluorophenyl)imino-3- (2-(1H-indol-3-yl)ethyl)-4-oxo-1,3-thiazolidin-5-yl]acetic acid (1)

White solid, Mp 122-124°C, yield: 76%. ^1^H NMR (300 MHz, DMSO-d_6_): δ 2.73-2.82 (m, 1H), 3.00-3.09 (m, 3H), 3.97-4.02 (m, 2H), 4.49 (dd, J = 3.9 Hz, 6.3 Hz, 1H), 6.82-6.87 (m, 1H), 6.90-6.93 (m, 1H), 6.96-7.01 (m, 1H), 7.05-7.10 (m, 1H), 7.21 (d, J = 2.4 Hz, 1H), 7.34-7.38 (m, 2H), 7.59 (d, J = 7.8 Hz, 1H), 10.86 (d, J = 2.4 Hz, 1H, NH), 12.77 (1H, COOH). ^13^C NMR (75 MHz, DMSO-d_6_): 22.4, 36.7, 43.0, 43.6, 110.6, 111.4, 117.4 (d, J_2_ = 21.8 Hz), 118.2, 118.4, 119.7(d, J_2_ = 18.0 Hz), 121.0, 121.4 (d, J_3_ = 7.5 Hz), 122.7, 123.3, 127.2, 136.3, 145.5 (d, J_4_ = 3.0 Hz), 154.1 (d, J_1_ = 241.5 Hz), 156.3 (2xC), 171.7, 173.3. HRMS (ESI) m/z 446.9008 calc. for C_21_H_17_ClFN_3_O_3_S [M+H]+; found 446.9011.

##### 2.1.2.2 {(2Z,5R)-2-ethylimino-3-[2-(1H-indol-3-yl)ethyl]-4-oxo-1,3-thiazolidin-5-yl}acetic acid (2)

White solid, Mp 118-119°C, yield: 82%. ^1^H NMR (300 MHz, DMSO-d6): δ 1.21 (t, J = 7.2 Hz, 3H, CH_3_), 2.69-2.78 (m, 1H), 2.92-3.05 (m, 3H), 3.28 (q, J = 7.2 Hz, 3H, CH_2_), 3.83-3.88 (m, 2H), 4.42 (dd, J = 3.9 Hz, 6.3 Hz, 1H), 6.96-7.01 (m, 1H), 7.04-7.07 (m, 1H), 7.17 (d, J = 2.4 Hz, 1H), 7.34 (d, J = 8.1 Hz, 1H), 7.62 (d, J = 7.5 Hz, 1H), 10.83 (d, J = 2.1 Hz, 1H, NH), 12.72 (1H, COOH). ^13^C NMR (75 MHz, DMSO-d6): 15.9, 22.5, 37.1, 42.7, 43.1, 46.0, 110.8, 111.4, 118.3 (2xC), 121.0, 122.9, 127.2, 136.3, 151.0, 171.7, 172.9. HRMS (ESI) m/z 346.4244 calc. for C_17_H_20_N_3_O_3_S [M+H]+; found 346.4246.

##### 2.1.2.3 {(2Z,5R)-3-[2-(1H-indol-3-yl)ethyl]-4-oxo-2-phenylimino-1,3-thiazolidin-5-yl}acetic acid (3)

White solid, Mp 133-135°C, yield: 79%. ^1^H NMR (300 MHz, DMSO-d_6_): δ 2.70-2.79 (m, 1H), 2.98-3.11 (m, 3H), 3.98-4.03 (m, 2H), 4.45 (dd, J = 3.6 Hz, 9.3 Hz, 1H), 6.88-6.91 (m, 2H), 6.96-7.01 (m, 1H), 7.05-7.16 (m, 2H), 7.23 (d, J = 2.1 Hz, 1H), 7.34-7.39 (m, 3H), 7.62 (d, J = 7.8 Hz, 1H), 10.87 (d, J = 2.4 Hz, 1H, NH), 12.75 (1H, COOH). ^13^C NMR (75 MHz, DMSO-d_6_): 22.4, 36.8, 43.0, 43.3, 110.6, 111.5, 118.1, 118.4, 120.9 (2C), 123.1, 124.2, 127.3, 129.3 (2C), 136.3, 148.3, 154.4, 171.7, 173.3. HRMS (ESI) m/z 394.4684 calc. for C_21_H_20_N_3_O_3_S [M+H]+; found 394.4687.

##### 2.1.2.4 {(2Z,5R)-2-cyclohexylimino-3-[2-(1H-indol-3-yl)ethyl]-4-oxo-1,3-thiazolidin-5-yl}acetic acid (4)

White solid, Mp 120-121°C, yield: 83%. ^1^H NMR (300 MHz, DMSO-d_6_): δ 1.07-1.41 (m, 5H), 1.49-1.77 (m, 5H), 2.18-2.5 (m, 1H), 2.64-2.70 (m, 1H*), 2.73-2.81 (m, 1H), 2.92-3.03 (m, 3H), 3.45 (t, J = 7.2 Hz, 2H), 3.45 (t, J = 7.5 Hz, 2H*), 4.34 (dd, J = 3.6 Hz, 8.7 Hz, 1H), 4.40 (dd, J = 3.6 Hz, 9.3 Hz, 1H*), 6.94-7.00 (m, 1H), 7.03-7.09 (m, 1H), 7.16 (d, J = 2.1 Hz, 1H), 7.30 (d, J = 8.1 Hz, 1H), 7.55 (d, J = 7.8 Hz, 1H), 7.63 (d, J = 7.8 Hz, 1H*), 10.81 (s, 1H, NH), 12.75 (1H, COOH). ^13^C NMR (75 MHz, DMSO-d_6_): 22.4, 25.7, 26.3 (C*), 27.6, 27.7 (C*), 33.3, 33.3 (C*), 37.3, 42.3, 42.7 (C*), 43.0, 53.1, 54.4 (C*), 110.7, 111.3, 111.4 (C*), 118.1, 118.2 (C*), 118.3 (C*), 118.4, 120.8, 121.0 (C*), 122.8, 122.9 (C*), 127.3 (C*), 127.4, 136.1, 136.2 (C*), 149.1 (C*), 151.3, 171.5, 171.7 (C*), 172.9 (C*), 173.2. HRMS (ESI) m/z 400.5164 calc. for C_21_H_26_N_3_O_3_S [M+H]+; found 400.5167.

##### 2.1.2.5 {(2Z,5R)-2-(2-chloro-5-methylphenyl)imino-3-[2-(1H-indol-3-yl)ethyl]-4-oxo-1,3-thiazolidin-5-yl}acetic acid (5)

White solid, Mp 129-131°C, yield: 87%. ^1^H NMR (300 MHz, DMSO-d_6_): δ 2.82-2.88 (m, 1H), 3.00-3.13 (m, 3H), 3.18 (s, 3H), 4.00-4.05 (m, 2H), 4.50 (dd, J = 3.6 Hz, 8.4 Hz, 1H), 4.57 (dd, J = 4.2 Hz, 7.8 Hz, 1H*), 6.88-6.76 (d, J = 2.4 Hz, 1H), 6.94-7.01 (m, 1H), 7.05-7.11 (m, 2H), 7.23 -7.25 (m, 1H), 7.35-7.38 (m, 1H), 7.42-7.48 (m, 1H), 7.60 (d, J = 7.8 Hz, 1H), 10.78 (s, 1H*, NH), 10.88 (s, 1H, NH), 12.87 (1H, COOH). ^13^C NMR (75 MHz, DMSO-d_6_): 16.9, 22.5, 22.6 (C*), 36.2 (C*), 36.6, 43.1, 43.3 (C*), 43.5, 110.5 (C*), 110.6, 111.3 (C*), 111.5, 118.1, 118.2 (C*), 118.3 (C*), 118.4, 119.6, 120.9 (C*), 121.1, 122.8 (C*), 123.2, 123.9 (C*), 123.9, 127.2 (C*), 127.2, 128.4 (C*), 128.4, 130.4, 132.0, 136.1 (C*), 136.3, 148.4 (C*), 148.4, 155.1 (C*), 155.4, 170.5 (C*), 171.6, 173.2 (C*), 173.4. HRMS (ESI) m/z 442.9374 calc. for C_22_H_21_ClN_3_O_3_S [M+H]+; found 442.9377.

##### 2.1.2.6 [(2Z,5R)-4-oxo-2-phenylimino-3-(1H-1,2,4-triazol-5-yl)-1,3-thiazolidin-5-yl]acetic acid (6)

White solid, Mp 154-156°C, yield: 34%. ^1^H NMR (300 MHz, DMSO-d_6_): δ 3.12-3.35 (m, 2H, CH_2_), 4.59 (s, 1H, CH), 6.84-7.60 (m, 5H), 8.58 (s, 1H, CH) 11.18 (s, 1H, NH), 11.80 (s, 1H, COOH). ^13^C NMR (75 MHz, DMSO-d_6_): 43.52, 51.73, 119.52, 120.60, 124.33 (2C), 125.50, 128.52 (2C), 128.81, 138.76, 166.88, 171,76. HRMS (ESI) m/z 318.3304 calc. for C_13_H_12_N_5_O_3_S [M+H]+; found 318.3307.

##### 2.1.2.7 [(2Z,5R)-2-(4-methylphenyl)imino-4-oxo-3-(1H-1,2,4-triazol-5-yl)-1,3-thiazolidin-5-yl]acetic acid (7)

White solid, Mp 157-159°C, yield: 29%. ^1^H NMR (300 MHz, DMSO-d_6_): δ 2.29 (s, 3H, CH_3_), 3.05-3.08 (m, 2H, CH_2_), 4.60 (s, 1H, CH), 7.16-7.32 (m, 3H), 7.46-7.48 (d, J = 8.4 Hz, 2H), 8.56 (s, 1H, CH) 11.13 (s, 1H, NH), 11.72 (s, 1H, COOH). ^13^C NMR (75 MHz, DMSO-d_6_): 20.55, 43.26, 51.71, 120.56 (2C), 124.36 (2C), 133.10 (2C), 134.76, 136.19, 145.69, 157.46, 166.77. HRMS (ESI) m/z 332.3574 calc. for C_14_H_14_N_5_O_3_S [M+H]+; found 332.3577.

##### 2.1.2.8 [(2Z,5R)-4-oxo-2-phenylimino-3-(1,3-thiazol-2-yl)-1,3-thiazolidin-5-yl]acetic acid (8)

White solid, Mp 144-146°C, yield: 72%. ^1^H NMR (300 MHz, DMSO-d_6_): δ 3.14-3.17 (m, 2H, CH_2_), 4.60-4.64 (m, 1H, CH), 7.40-7.42 (m, 3H), 7.43-7.48 (m, 3H), 7.60-7.61 (d, J = 2.1 Hz, 1H), 7.60-7.61 (d, J = 1.5 Hz, 1H), 12.82 (s, 1H, COOH). ^13^C NMR (75 MHz, DMSO-d_6_): 43.38, 60.83, 117.26, 120.71, 124.18, 129.21 (2C), 135.40, 137.71, 139.86, 148.21, 155.39, 161.45, 169.01. HRMS (ESI) m/z 334.3874 calc. for C_14_H_12_N_3_O_3_S_2_ [M+H]+; found 334.3876.

##### 2.1.2.9 [(2Z,5R)-2-(4-methoxyphenyl)imino-4-oxo-3-(1,3-thiazol-2-yl)-1,3-thiazolidin-5-yl]acetic acid (9)

White solid, Mp 139-141°C, yield: 80%. ^1^H NMR (300 MHz, DMSO-d_6_): δ 3.12-3.14 (m, 2H, CH_2_), 3.81 (s, 3H, OCH_3_), 4.55-4.59 (m, 1H, CH), 7.03-7.10 (m, 3H), 7.23-7.31 (m, 3H), 7.39-7.40 (d, J = 3.6 Hz, 1H), 7.59-7.60 (d, J = 3.6 Hz, 1H), 12.78 (s, 1H, COOH). ^13^C NMR (75 MHz, DMSO-d_6_): 43.48, 55.35, 114.27 (2C), 117.17, 121.76, 127.80, 129.48 (2C), 139.83, 141.26, 159.20, 161.68, 171.76, 173.46. HRMS (ESI) m/z 364.4134 calc. for C_15_H_14_N_3_O_3_S_2_ [M+H]+; found 364.4136.

##### 2.1.2.10 [(2Z,5R)-2-(4-iodophenyl)imino-4-oxo-3-(1,3-thiazol-2-yl)-1,3-thiazolidin-5-yl]acetic acid (10)

White solid, Mp 162-165°C, yield: 83%. ^1^H NMR (300 MHz, DMSO-d_6_): δ 3.13-3.17 (m, 2H, CH_2_), 4.57-4.65 (m, 1H, CH), 7.20-7.23 (m, 2H), 7.23-7.31 (m, 3H), 7.40-7.41 (d, J = 2.4 Hz, 1H), 7.60-7.61 (d, J = 2.1 Hz, 1H), 12.75 (s, 1H, COOH). ^13^C NMR (75 MHz, DMSO-d_6_): 43.75, 60.91, 115.35, 117.38, 123.34 (2C), 130.71 (2C), 138.04 (2C), 147.81, 156.85, 161.20, 168.88. HRMS (ESI) m/z 460.2839 calc. for C_14_H_11_IN_3_O_3_S_2_ [M+H]+; found 460.2842.

##### 2.1.2.11 [(2Z,5R)-2-(2-chlorophenyl)imino-4-oxo-3-(1,3-thiazol-2-yl)-1,3-thiazolidin-5-yl]acetic acid (11)

White solid, Mp 147-148°C, yield: 72%. ^1^H NMR (300 MHz, DMSO-d_6_): δ 3.27-3.34 (m, 2H, CH_2_), 5.73-5.75 (m, 1H, CH), 7.41-7.65 (m, 5H), 7.67-7.70 (t, J = 3.6 Hz, 1H), 7.82-7.88 (m, 1H), 12.84 (s, 1H, COOH). ^13^C NMR (75 MHz, DMSO-d_6_): 34.04, 60.76, 115.55, 123.03, 129.11, 130.61, 131.88, 132.66, 133.29, 137.82, 156.49, 170.86 (2C), 177.63. HRMS (ESI) m/z 368.8294 calc. for C_14_H_11_ClN_3_O_3_S_2_ [M+H]+; found 368.8297.

##### 2.1.2.12 [(2Z,5R)-2-(4-bromophenyl)imino-4-oxo-3-(1,3-thiazol-2-yl)-1,3-thiazolidin-5-yl]acetic acid (12)

White solid, Mp 150-152°C, yield: 69%. ^1^H NMR (300 MHz, DMSO-d_6_): δ 3.13-3.15 (m, 2H, CH_2_), 4.56-4.60 (m, 1H, CH), 7.24-7.27 (m, 2H), 7.40-7.53 (m, 3H), 7.60-7.62 (d, J = 3.6 Hz, 1H), 7.67-7.68 (d, J = 3.6 Hz, 1H), 12.86 (s, 1H, COOH). ^13^C NMR (75 MHz, DMSO-d_6_): 36.25, 43.70, 117.35, 127.36, 128.63, 131.54, 133.11, 134.10, 136.45, 139.89, 161.29, 168.88, 171.84. 173.87. HRMS (ESI) m/z 413.2834 calc. for C_14_H_11_BrN_3_O_3_S_2_ [M+H]+; found 413.2837.

##### 2.1.2.13 [(2Z,5R)-2-(3,4-dichlorophenyl)imino-4-oxo-3-(1,3-thiazol-2-yl)-1,3-thiazolidin-5-yl]acetic acid (13)

White solid, Mp 122-125°C, yield: 71%. ^1^H NMR (300 MHz, DMSO-d_6_): δ 3.66 (s, 2H, CH_2_), 4.56-4.61 (t, J = 6.0 Hz, 1H, CH), 7.36-7.44 (m, 2H), 7.40-7.53 (m, 2H), 7.61-7.63 (d, J = 3.6 Hz, 1H), 7.72-7.73 (d, J = 2.1 Hz, 1H), 12.93 (s, 1H, COOH). ^13^C NMR (75 MHz, DMSO-d_6_): 36.28, 43.83, 117.51, 129.18, 130.55, 131.15, 131.29, 131.79, 135.03, 139.92, 161.02, 166.82, 168.70, 170.91. HRMS (ESI) m/z 403.2714 calc. for C_14_H_10_Cl_2_N_3_O_3_S_2_ [M+H]+; found 403.2717.

##### 2.1.2.14 [(2Z,5R)-2-(3-chloro-4-methylphenyl)imino-4-oxo-3-(1,3-thiazol-2-yl)-1,3-thiazolidin-5-yl]acetic acid (14)

White solid, Mp 146-148°C, yield: 77%. ^1^H NMR (300 MHz, DMSO-d_6_): δ 3.57 (s, 3H, CH_3_), 4.42-4.43 (m, 2H, CH_2_), 5.68-5.70 (d, J = 7.2 Hz, 1H, CH), 7.34-7.38 (m, 3H), 7.57-7.58 (d, J = 2.1 Hz, 1H), 7.70-7.72 (d, J = 3.9 Hz, 2H), 12.90 (s, 1H, COOH). ^13^C NMR (75 MHz, DMSO-d_6_): 45.98, 50.95, 116.96, 120.83, 121.79, 123.21, 130.92, 131.52, 131.98, 134.81, 139.84, 162.74, 166.88, 169.06, 169.31. HRMS (ESI) m/z 382.8564 calc. for C_15_H_13_ClN_3_O_3_S_2_ [M+H]+; found 382.8567.

##### 2.1.2.15 [(2Z,5R)-2-(3-chloro-4-fluorophenyl)imino-4-oxo-3-(1,3-thiazol-2-yl)-1,3-thiazolidin-5-yl]acetic acid (15).

White solid, Mp 147-149°C, yield: 82%. ^1^H NMR (300 MHz, DMSO-d_6_): δ 3.34-3.35 (m, 2H, CH_2_), 4.56-4.60 (t, J = 3.0 Hz, 1H, CH), 7.41-7.61 (m, 3H), 7.61-7.64 (m, 1H), 7.66-7.70 (m, 2H), 12.85 (s, 1H, COOH). ^13^C NMR (75 MHz, DMSO-d_6_): 43.76, 48.60, 117.32, 117.46, 117.62, 119.60, 119.86, 129.68, 129.78, 130.74, 132.09, 132.14, 139.91, 161.18. HRMS (ESI) m/z 386.8198 calc. for C_14_H_10_ClFN_3_O_3_S_2_ [M+H]+; found 386.8201.

The example NMR spectra are given in the Supplementary file (see Figs. **S1**-**S30**).

### 2.2 X-Ray Studies

The experiment was performed on a SuperNova, single-source at offset/far, Eos diffractometer. The crystal was kept at 100.00(10) K during data collection. Using Olex2 [37], the structure was solved with the XS structure solution program using Direct Methods and refined with the XL refinement package using Least Squares minimization.

### 2.3 Biological Assay

#### 2.3.1 Cell Line and Culture

The human cell lines utilized in this study included MDA-MB-231 (breast adenocarcinoma), SW620 (metastatic colorectal carcinoma), and HaCaT (immortalized human keratinocytes), all obtained from the American Type Culture Collection (ATCC, Rockville, USA). Cell culturing was conducted under specific conditions: HCT116 and SW620 cells were maintained in Minimum Essential Medium (MEM; Thermo Fisher Scientific, Waltham, MA, USA), whereas MDA-MB-231 and HaCaT cells were grown in Dulbecco’s Modified Eagle Medium (DMEM) with high glucose content. The culture media were supplemented with 10% fetal bovine serum (FBS; Sigma-Aldrich, St. Louis, MO, USA), 20 mM HEPES buffer (Biowest, Nuaillé, France), and antibiotics (100 U/mL penicillin and 100 µg/mL streptomycin; Gibco, Grand Island, NY, USA). All cell lines were incubated at 37°C in a humidified atmosphere containing 5% CO_2_ until reaching approximately 80-90% confluence [36].

#### 2.3.2 MTT Assay

To assess cytotoxic properties, the MTT assay was employed across three distinct cell lines, with results expressed as IC_50_ values. The selected cancer models included human metastatic colon carcinoma (SW620) and human breast adenocarcinoma (MDA-MB-231). As a reference for selectivity, a non-cancerous human keratinocyte line derived from adult skin (HaCaT) was used, and results were further evaluated using the Selectivity Index (SI).

The MTT assay is based on the enzymatic reduction of the yellow tetrazolium salt, 3-(4,5-dimethylthiazol-2-yl)-2,5-diphenyltetrazolium bromide (MTT), into purple formazan crystals by mitochondrial dehydrogenases in metabolically active cells.

Cells were seeded into 96-well plates at a density of 1 × 10^4^ cells per well and allowed to adhere during a 24-hour incubation period. Subsequently, they were treated with varying concentrations (20-100 µM) of 1,3-thiazolidin-5-yl derivatives, while the control group remained untreated. After a 72-hour incubation at 37°C in a humidified atmosphere with 5% CO_2_, MTT solution (0.5 mg/mL) was added to each well, followed by an additional 4-hour incubation under the same conditions. To solubilize the resulting formazan crystals, a 1:1 (*v/v*) mixture of DMSO and isopropanol was used.

Absorbance was measured at 570 nm using a MultiscanGo microplate reader (Thermo Scientific). Cell viability was calculated as the percentage of MTT reduction in treated cells relative to the untreated control, using the formula: ([A]/[B]) × 100%, where [A] represents the absorbance of treated cells and [B] denotes the absorbance of the control group. Lower MTT values indicate reduced viability. IC_50_ values, defined as the concentration of compound that inhibits 50% of cell viability, were determined using GraphPad Prism 8.0.1 software [36]. The MTT assay was conducted across a concentration range of 1 to 100 μM. For compounds that failed to induce at least 50% inhibition of cell viability at 100 μM, IC_50_ values were recorded as “>100 μM” to indicate the absence of significant cytotoxicity within the tested range.

#### 2.3.3 Cell Cycle and Apoptosis

##### 2.3.3.1 Cell Cycle Analysis

SW620 and MDA-MB-231 cells were seeded in six-well plates at a density of 1 × 10^5^ cells per well and incubated for 24 hours to facilitate attachment. Following this period, compounds 5, 6, and 7 were introduced at concentrations equivalent to their respective IC_50_ values, excluding the control wells, and cells were incubated for an additional 24 hours. Subsequently, both adherent and non-adherent cells were harvested and centrifuged at 400 × g for 5 minutes at 4°C. The cell pellets were washed twice with 0.9% NaCl and fixed overnight in 500 µL of pre-cooled 70% ethanol.

Prior to flow cytometric analysis, samples were centrifuged at 850 × g for 5 minutes at 4°C and rinsed with phosphate-buffered saline (PBS). Cells were then incubated at 37°C for 30 minutes in the dark with 50 µL of RNase (100 µg/mL) and 200 µL of propidium iodide (PI; 50 µg/mL). Before measurement, each sample was diluted with an additional 100 µL of PBS. Cell cycle distribution was analyzed using a FACSVerse flow cytometer (Becton Dickinson, Franklin Lakes, NJ, USA). All experiments were carried out in quadruplicate [34].

##### 2.3.3.2 Apoptosis

SW620 and MDA-MB-231 cells were seeded into 6-well plates at a density of 1 × 10^5^ cells per well and incubated for 24 hours to promote cell attachment. Following this, compounds 5, 6, and 7 were administered at concentrations corresponding to their respective IC_50_ values, while control wells remained untreated. Cells were then incubated for an additional 72 hours.

Apoptosis was assessed using the commercially available FITC Annexin V Apoptosis Detection Kit I (Biosciences Pharmingen, San Jose, CA, USA). After treatment, cells were harvested, washed with phosphate-buffered saline (PBS), and stained with Annexin V-FITC and propidium iodide (PI), following the manufacturer's instructions (Becton Dickinson). Flow cytometry analysis was performed using a FACSVerse instrument (Becton Dickinson, Franklin Lakes, NJ, USA) to evaluate apoptotic status. Based on staining, cells were categorized as early apoptotic (Annexin V+/PI−) or late apoptotic/necrotic (Annexin V+/PI+) [34].

#### 2.3.4 Human Interleukin 6

To assess interleukin-6 (IL-6) production, a human IL-6 ELISA kit (Diaclone SAS, Besançon Cedex, France) was used. The SW620 and MDA-MB-231 cell lines were treated with compounds 5, 6, and 7 at concentrations equivalent to their IC_50_ values and incubated for 72 hours. Non-treated cells were included as a control group. Following incubation, culture supernatants were collected and analyzed for IL-6 content using the enzyme-linked immunosorbent assay method, following the supplier’s instructions. Each experimental condition was performed in triplicate.

#### 2.3.5 *In Silico* Studies

##### 2.3.5.1 Molecular Docking

To investigate interactions with the Epidermal Growth Factor Receptor, we performed pocket-based molecular docking of 15 studied molecules against 5 different crystal structures of the receptor, each featuring a defined active site and a confirmed antagonist of the receptor under study (1M17, 2ITO, 4I23, 4LQM, 7AEM). In selecting the crystal structures, we aimed for the greatest possible diversity in the Shape of the Binding Pocket (pocket lDDT metric) and Tanimoto Ligand Similarity while maintaining the appropriate antagonist properties of the native ligand.

Molecular docking was performed using the classical software AutoDock Smina 1.1.2 [38], targeting the native ligand pocket with an additional amount of buffer space measuring 4 Å. Subsequently, the docking scoring function results were compared with the results for native ligands. Visualizations were presented using PyMOL software.

##### 2.3.5.2 Molecular Dynamics Simulation

Molecular dynamics simulations for selected complexes were performed using OpenMM 8.2.0 [39]. Prior to this, the protein was properly repaired by adding missing residues and amino acids, as well as standardizing non-standard residues. This process was carried out using MODELLER 10.6 [40] and the PDBFixer package.

For the dynamics simulations, the Amber ff14SB force field [41] was applied for the protein, while the gaff-2.11 force field was used for the ligand. The entire complex was placed in a solvent using the TIP3P water model with a padding of 10.0 Å. The ion concentration was set to 150 mM NaCl.

The prepared system underwent a multi-step preliminary dynamics process, which included the following stages: energy minimization, heating from 50 to 300 K, release of backbone restraints, and equilibration under NVT conditions, followed by NPT conditions. After these preparations, a full molecular dynamics simulation of the complex was conducted for 100 ns with a 1ps timestep and 100ps write interval.

The detailed parameters for the dynamics and each stage of the preparation process are presented in Table **1**.

Subsequently, the trajectories were analyzed using the PyTraj 2.0.2 python package [42], and free energy calculations were performed *via* gmx_MMPBSA 1.6.4 software [43]. MM/PBSA [44] and MM/GBSA [45] were calculated for every 100 simulation snapshots.

When analyzing molecular dynamics by the protein trajectory, we refer to the trajectories of all C-alpha atoms in the protein. By the ligand trajectory, we consider all atoms of the ligand molecule except for hydrogen. Finally, the pocket trajectory encompasses the amino acids located within a 6 Å distance from the native ligand in the first frame of its simulation.

##### 2.3.5.3 ADMET Prediction

To perform the prediction of key-druglike parameters, the open source ADMET-AI python package [46] was used in our local batch environment. The ottaineddata file of predictions was given to standardization using MaxAbsScaller, which is an estimator for scaling each feature by its maximum absolute value, ensuring that all values lie between -1 and 1 while preserving the data's sparsity. Then, a standardized data file was provided to hierarchical clustering using the Ward method, which groups compounds based on their similarities across selected molecular properties. The ward method minimizes the variance within each cluster, creating a more distinct clustering pattern. To create a visualization as a heatmap with a dendrogram, the cluster map function from Seaborn was used, which automatically performs hierarchical clustering and generates a heatmap with a dendrogram. To create a radial chart, standard python coding with the NumPy library was used. To display the plots with matplotlib.pyplot library was used.

#### 2.3.6 Statistical Analysis

Statistical evaluations were performed using GraphPad Prism 9 (San Diego, CA, USA). Results are expressed as mean ± standard deviation (SD) based on a minimum of three independent experiments. Differences between groups were analyzed using one-way ANOVA, followed by Dunnett’s post hoc test for multiple comparisons. A p-value of less than 0.05 was considered statistically significant [34].

## 3 RESULTS AND DISCUSSION

### 3.1 Chemistry

#### 3.1.1 Synthesis

In this paper, we disclose fifteen novel (2-imino-4-oxo-1,3-thiazolidin-5-yl)acetic acid derivatives. The synthetic strategy involved obtaining five compounds in each group based on the heterocyclic substituent used (Table **2**). We aimed to test the effectiveness of the reaction in terms of yields, universality, and biological activity outcomes. The obtained derivatives were categorized into three sets based on the heterocyclic rings attached to the core 4-thiazolidinone motif: tryptamine, thiazole, and 1,2,4-triazole. The synthetic route was consistent across all derivatives, involving a reaction between a thiourea derivative and maleic anhydride, resulting in the formation of thiazolidin-4-one ring analogs through cyclization (Fig. **2**).

Based on previous reports, acetone [47] was selected as the solvent for this reaction to predefine regioselectivity, as the nucleophilic attack by the nonequivalent nitrogen atoms of the thiourea could result in varying ratios of regioisomers. We can confirm that the use of acetone promoted the formation of a single regioisomer, as reported in the previous studies [48, 49].

However, the synthesis of 4-thiazolidinones combined with a 1,2,4-triazole ring resulted in unsatisfactory yields due to the low solubility of the triazole substrate under the reaction conditions. This limitation led to the formation of only two derivatives in this group (compounds **6** and **7**). Despite this, the promising preliminary anticancer screening results for these compounds motivated us to include them in further tests, which are detailed in this article. Additionally, we plan to perform separate studies on a larger group of 4-thiazolidinone and triazole hybrids, applying alternative synthetic pathways to improve yields, solubility, and stereochemical purity.

The structures of all obtained products were confirmed using NMR spectroscopic analysis, including ^1^H and ^13^C NMR, revealing characteristic chemical shifts consistent with the proposed molecular frameworks. In the case of ^1^H NMR, the most identifiable were: the methine proton (adjacent to carbonyl, and attached to the acetic acid group), the methylene protons of the CH_2_COOH side chain, and the COOH proton. For ^13^C NMR: the C=O (lactam carbon), the C=N (imino group), and the COOH carbon (carboxylic acid).

It should be noted that for (2-imino-4-oxo-1,3-thiazolidin-5-yl)acetic acid derivatives, tautomerism and H-bonding may appear and influence the NH, COOH, or other group signals not only due to the central 4-thiazolidinone skeleton, but also due to the chosen heterocycles. The Supplementary file includes the specific data. In addition, confirmation of stereochemistry was achieved *via* X-ray analysis of the monocrystal of compound **2** (see section 2.1.2). Such a study is widely considered the gold standard or final proof for structure confirmation because it provides direct, three-dimensional atomic-level details of a molecule.

#### 3.1.2 X-ray Diffraction Studies

After synthesis, compound **2** was successfully submitted for crystallization using a 1:1 mixture of water and absolute ethanol, resulting in the isolation of a monocrystal structure. This allowed us to perform X-ray diffraction studies, which confirmed the regioselective nature of the presented synthetic route under the given conditions and previously described formation of a single regioisomer. Crystal data and structure refinement are summarized in Table **3**. The Cambridge Crystallographic Data Centre (CCDC 2456121) contains the supplementary crystallographic data for this paper. These data can be obtained free of charge *via *https://www.ccdc.cam.ac.uk/structures/. To our knowledge, this is the first crystal structure of (2-imino-4-oxo-1,3-thiazolidin-5-yl)acetic acid derivative reported to date.

Compound **2** crystallizes in the monoclinic crystal system, belonging to the space group P2_1_/c. The asymmetric unit contains one molecule of {(2Z,5R)-2-ethylimino-3-[2-(1*H*-indol-3-yl)ethyl]-4-oxo-1,3-thiazolidin-5-yl}acetic acid, along with two water molecules (Fig. **3**). The crystal packing reveals several intermolecular N-H···O hydrogen bonds, including N_3_-H_3_···O_20_ (2.794 Å) and N_22_-H_22_···O21 (2.745 Å), as illustrated in Fig. (**4**). Moreover, the overall structure is further stabilized by O-H···O hydrogen bonding interactions involving the water molecules, with bond lengths ranging from 2.766 to 3.007 Å.

This unequivocal structural confirmation not only validates the regio- and stereochemical integrity of our synthetic route but also provides a solid foundation for the subsequent biological evaluation of these thiazolidinone derivatives in cytotoxicity assays in the next steps of this research.

### 3.2 Cytotoxic Activity

A preliminary assessment of cytotoxic activity was performed for all newly obtained compounds (Table **4**). It was expressed in IC_50_ values and established using the MTT method. The tumor cells used in the study were derived from human metastatic colon cancer (SW620) and human breast adenocarcinoma (MDA-MB-231) cell lines. The results of IC_50_ values against cancer cell lines were compared to those obtained from tests with the human immortal keratinocyte cell line (HaCaT), representing normal cells, and expressed as selectivity index (SI).

Most of the new 4-thiazolidinone derivatives from the first set, containing a tryptamine ring, were found not to be active toward the studied cancer cell lines. However, compound **5** bearing a 2-chloro-4-methylophenyl substituent exhibited significant cytotoxic activity at a level of IC_50_ =21.16 ± 0.36 µM, registered against the MDA-MB-231 cancer cell line. It is worth emphasizing that HaCaT cells were not affected by compound **5,** which resulted in a high selectivity index of 4.73. In the group of 4-thiazolidinones containing a thiazole ring, most of the compounds did not show antiproliferative activity. The best result in this group was observed for compound **15**, presenting IC_50_= 53.24 ± 2.80 µM against SW620 cell line.

In general, the thiazole-substituted derivatives exhibited the weakest cytotoxic activity among the tested compounds, with several showing IC_50_ values above 100 μM. This decreased potency may be attributed to the electronic and steric characteristics of the thiazole moiety, which introduces planarity and rigidity that may impair optimal receptor binding. In contrast to the more flexible triazole and tryptamine derivatives, the thiazole ring may reduce compound permeability or hinder critical interactions within the active site of molecular targets.

The 1,2,4-triazole derivatives (compounds **6** and **7**) contained phenyl and 4-methylophenyl substituents, respectively, and exhibited significant cytotoxic activity. Compound **7** showed IC_50_= 36.82 ± 2.78 µM and IC_50_= 24.10 ± 3.98 µM, against MDA-MB-231 and SW620 cancer cells, respectively. Compound **6** had slightly lower activity, with an IC_50_= 41.31 ± 1.98 µM (SW620). Neither of the compounds exhibited cytotoxic properties toward the normal human cell line (HaCaT), giving IC_50_ values greater than 100 µM. This indicated high selectivity indexes for compounds **6** and **7** at 2.42 and 4.16, respectively.

Based on their favorable antiproliferative profiles observed in the MTT assay, particularly in the MDA-MB-231 cell line and extraordinary selectivity, compounds **5**, **6**, and **7** were selected for further investigation. These derivatives exhibited the most pronounced cytotoxic activity within the tested series. Their enhanced performance suggested a promising structure-activity relationship, warranting more detailed mechanistic and molecular docking studies to elucidate their potential mode of action and interaction with oncogenic targets.

### 3.3 Investigation of Anticancer Mechanism

#### 3.3.1 Apoptotic Activity

Due to the results from preliminary cytotoxic activity evaluations against human metastatic colon cancer (SW620) and human breast adenocarcinoma (MDA-MB-231) cell lines, compounds **5**, **6**, and **7** were selected for further testing to investigate their mechanism of action. To establish the apoptotic effects of the novel (2-imino-4-oxo-1,3-thiazolidin-5-yl)acetic acid derivatives on these cancer cell lines, flow cytometry followed by annexin V-FITC and PI staining was used. As shown in Figs. (**5** and **6**), we observed significant differences in the number of cells in the apoptotic state in samples treated with the studied compounds compared to the untreated control group, suggesting that these compounds may possess proapoptotic properties. Overall, MDA-MB-231 cells demonstrated greater sensitivity to the studied compounds in inducing apoptosis compared to SW620 cells.

Compound **7** (Figs. **5** and **6**) had the strongest proapoptotic effect, leading 6.60 ± 0.69% (*p* ≤ 0.0001) and 2.38 ± 0.15% (*p* ≤ 0.001) of MDA-MB-231 and SW620 cells, respectively, to late apoptosis or necrosis, compared to 0.15 ± 0.029% for MDA-MB-231 and 1.05 ± 0.07% for SW620 in the control groups. Compound **6** acted similarly towards SW620 cells, resulting in 2.91 ± 0.12% (*p* ≤ 0.0001) of cells being registered in the stage of late apoptosis or necrosis (Figs. **5** and **6B**). Furthermore, derivative **5** led a significant number of MDA-MB-231 cells to enter the state of late apoptosis or necrosis (Figs. **5** and **6A**) with a percentage of 5.19 ± 0.17% (*p* ≤ 0.0001), compared to 0.93 ± 0.015% in the control group.

The results obtained from the apoptosis assay confirmed the observations from the MTT experiment, revealing the properties of compounds **5**, **6,** and **7** in inducing a moderate number of cells to late apoptosis and necrosis. Additionally, MDA-MB-231 cancer cells showed slightly higher sensitivity to this effect. This suggests that the antiproliferative properties of the studied compounds may be related to their potential to induce proapoptotic effects in cancer cells.

Since apoptosis often arises from earlier disruptions of cell-cycle control, we asked whether the same compounds that triggered late-stage cell death might also halt proliferation at specific checkpoints. Therefore, after confirming that derivatives **5**, **6**, and **7** significantly increased the fraction of cells in late apoptosis or necrosis, we analyzed their impact on cell-cycle distribution in both MDA-MB-231 and SW620 cell lines. The next section details how these compounds influence the G1, S, and G2/M phase populations, shedding light on whether cell-cycle arrest precedes and possibly drives the proapoptotic effects observed above.

#### 3.3.2 Induction of Cell Cycle Arrest

The cell cycle is a series of events, including DNA duplication, protein translation, and gene transcription, which take place in a single cell before it divides into two new cells [50]. Dysregulation of crucial cell cycle proteins and the cell cycle itself is a hallmark of cancer cells, enabling their uncontrolled proliferation and growth. Thus, therapies targeting dysregulated cell cycle processes in cancer cells can potentially hinder their development [51-53].

To evaluate the effect of compounds **5**, **6**, and **7** on cell cycle progression in MDA-MB-231 and SW620 cancer cells, the cells were exposed to IC_50_-equivalent concentrations of the compounds and incubated for 24 hours. Cell cycle distribution was then analyzed using flow cytometry.

Both compounds **5** and **7** influenced cell cycle progression in MDA-MB-231 cells, although their effects differed (Figs. **7** and **8A**). Compound **7** caused cell cycle arrest in the G0/G1 phase, as indicated by a higher percentage of cells in this phase compared to untreated controls. A reduction in the proportion of cells in the S and G2/M phases accompanied this. These findings imply that compound **7** may exhibit antiproliferative activity, potentially promoting apoptosis, which is further supported by a modest increase in the sub-G1 fraction, representing apoptotic or dead cells. In contrast, compound **5** led to an accumulation of cells in the G2/M phase, with a concurrent decline in the S phase population, suggesting a different mechanism of cell cycle disruption.

In SW620 colorectal cancer cells, compound **7** demonstrated the most pronounced effect, significantly increasing the proportion of cells in the G2/M phase, alongside a rise in the S phase population, relative to the control group (Figs. **7** and **8B**). This was coupled with a marked reduction in G0/G1 phase cells (48.89%, *p* ≤ 0.001), suggesting cell cycle arrest at a later checkpoint. Treatment with compound **6** resulted in a 1.7-fold increase in the S phase cell population (*p* ≤ 0.05), along with a decrease in cells residing in the G0/G1 phase. Furthermore, exposure to compound **6** led to a 2.77-fold increase in the sub-G1 fraction compared to the control, indicating elevated levels of apoptosis or cell death.

The results obtained from the cell cycle investigation are consistent with those from previous analyses. The induction of cell cycle arrest in the G0/G1 phase and an increased population of cells in the sub-G1 phase, representing dead cells, confirmed the proapoptotic potential of compounds **6** and **7**. Compound **5** demonstrated antiproliferative potential through inhibition of cell growth in the S phase, preventing cells from progressing to mitosis, which occurs in the G2/M phase.

Because checkpoint blockade and apoptosis are often modulated by inflammatory cues—especially the IL-6/JAK-STAT3 axis, which can either push tumor cells into G0/G1 arrest or, conversely, promote survival depending on context [54, 55] —we next examined whether our thiazolidinone derivatives alter IL-6 signaling. The following section therefore, reports how compounds **5**, **6,** and **7** affect IL-6 secretion and pathway activation in MDA-MB-231 and SW620 cultures, clarifying whether modulation of this cytokine accompanies the cell cycle and apoptotic changes described above.

#### 3.3.3 Human Interleukin 6

To better understand the cytotoxic mechanisms of action, (2-imino-4-oxo-1,3-thiazolidin-5-yl)acetic acid derivatives (compounds **5**, **6**, and **7**) were subjected to a human interleukin-6 (IL-6) assay (Fig. **9**). IL-6 is a prototypical, pro-inflammatory cytokine involved in maintaining homeostasis [56] and is overexpressed in almost every type of cancer. A higher concentration of IL-6 has been shown to promote tumorigenesis by regulating crucial metabolic and signaling pathways. Therefore, compounds that reduce IL-6 secretion in cancer cells can be considered potential antitumor agents [57].

To conduct a Human IL-6 test, previously used cancer cells were treated with the compounds at concentrations corresponding to their IC_50_ values. Among the human cancer cell lines used in the study, MDA-MB-231 was identified as more sensitive to the inhibition of IL-6 release compared to SW620. The strongest effect on MDA-MB-231 cells was observed with compound **5**, which inhibited IL-6 secretion by a factor of 4.1, compared to the control group (*p*≤ 0.001).

However, for compound **7**, the registered concentration of IL-6 was 6.63±1.76 pg/mL, compared to 8.67±1.54 pg/mL in the control group, indicating its moderate inhibitory influence on the same cancer cell line (*p*≤ 0.01). For SW620 cells, inhibition of IL-6 secretion was observed at levels of 1.38-fold and 2.21- fold compared to the control group, for compounds **6** and **7**, respectively.

The reduction of IL-6 concentration in cells caused by the studied compounds complements the previous results from cytotoxic evaluation tests. MDA-MB-231 cells were shown to be more sensitive to exposure to the studied compounds compared to the SW620 cancer cell line. The strongest effect was registered for compound **5**, which inhibited IL-6 secretion by 76%, compared to the control group.

Having shown that compounds **5**, **6**, and **7** stall the cell cycle and drive apoptosis, we next asked how these molecules might engage their protein targets at the atomic level. Therefore, the following section presents our molecular docking studies, in which each compound was fitted into the active sites of key cancer-related kinases. These *in silico* results help explain the distinct cell cycle checkpoints affected by each derivative and provide a structural basis for the antiproliferative trends observed *in vitro*.

### 3.4 *In Silico* Studies

#### 3.4.1 Molecular Docking and Molecular Dynamics Simulation

Molecular docking is a bioinformatics technique used to predict the conformation of a ligand within the active site of a macromolecule and to estimate the binding affinity of the resulting protein-ligand complex. This prediction is guided by strictly defined scoring functions. It is important to emphasize that molecular docking primarily serves as a preliminary screening tool, paving the way for more complex and computationally intensive molecular dynamics simulations of the complex.

The MDA-MB-231 cancer cell line demonstrated greater sensitivity to the studied compounds during preliminary evaluations of cytotoxicity, apoptosis induction, and cell cycle arrest. Scientific reports have shown that various types of human breast cancer, including human breast adenocarcinoma, often exhibit overexpression of the epidermal growth factor receptor (EGFR) [58]. Moreover, combined therapy of the well-known antitumor agent doxorubicin with EGFR inhibitors improved the efficacy of cytotoxic action and was identified as a synergistic interaction [59].

To determine whether the cytotoxic mechanism of the candidate compounds (**5**, **6,** and **7**) identified in the *in vitro* phase is associated with potential interference with EGFR receptor, we conducted docking experiments using five crystal structures of EGFR (1M17, 2ITO, 4I23, 4LQM, and 7AEM) [60-64], each featuring defined active pockets. These structures correspond to conformations bound with five different receptor antagonists: erlotinib, gefitinib, dacomitinib, PD-168393, and brigatinib (Fig. **10**). We evaluated the three studied molecules (compounds **5**, **6**, and **7**) against these active pockets.

This preliminary study aimed to evaluate whether compounds **5**, **6**, and **7**, later discussed as ligands, possess the biophysical potential to act as antagonists for EGFR. Such findings could provide valuable insights into their mechanism of action, which, despite existing hypotheses based on known pharmacodynamic effects, remains unconfirmed. Notably, the scoring function results for these compounds significantly exceeded those of the native ligands, as shown in Table **5**.

Through the docking results obtained from the static crystal structures, we derived not only a numerical evaluation of binding affinity but also a spatial representation of the molecular conformation. This spatial arrangement served as the initial positioning for subsequent molecular dynamics simulations. In total, we performed four simulations: three involving the target molecules within the most promising binding pocket (7AEM), and one with the same pocket bound to the native ligand.

The best-docked poses from each complex, along with the native pose of the original ligand, were used as starting points for our molecular dynamics simulations. From the generated trajectories, we analyzed and compared changes in the RMSD (root mean square deviation) of the protein's C-alpha atoms, the active site, and the entire ligand. Additionally, we evaluated the RMSF (root mean square fluctuation) of protein residues, with particular emphasis on those in the active site and the ligand atoms.

The first stage of analysis involved calculating the superimposed root mean square deviation (RMSD) of atomic positions, using the first simulation frame as the reference. As shown in Figs. (**11** and **12**) and Table **6**, the RMSD of protein trajectories revealed stable mean values (~5-6 Å) with low deviations in the presence of compounds **5**-**7**, indicating improved global structural stability over time. In contrast, the RMSD of ligands **6** and **7** showed similar values, suggesting comparable dynamic behavior, while the native ligand and compound **5** exhibited lower average RMSD values (~1.2 Å), which may reflect difficulties in achieving a thermodynamically stable conformation. A similar trend was observed in the active site residues. Except in the presence of compound **5**, the active site RMSD values remained relatively constant, averaging below 1.5 Å.

In contrast, the RMSF analysis indicated greater flexibility in the heavy atoms of the native ligand compared to the studied molecules. This observation suggests that the tested compounds may align more effectively with the active site. The increased flexibility of the native ligand could be attributed to its larger size and the spatial constraints of the active site pocket. Consequently, larger ligands, such as the native ligand, may exhibit less favorable interactions compared to smaller molecules like compounds **5**-**7**.

Surprisingly, the analysis of free binding energy using two methods (Table **7**) revealed that, despite the significant mobility of the native ligand, it exhibited the most stable affinity towards the protein. In contrast, molecule **6** displayed a different performance due to its relatively constant intermolecular van der Waals interactions (Figs. **11** and **12**), which led to a significant and unfavorable increase in binding energy. A detailed analysis of the trajectory movements of the native ligand and molecule **6** complexes, visualized in Fig. (**13**), highlights that the stability of the native ligand arises from its incomplete embedding within the active pocket. For the native ligand, only a portion of its atoms interacts with the pocket, while the remaining atoms, responsible for high fluctuations, rotate freely at a greater distance from the protein surface.

Molecular docking and dynamics studies were complementary to previous results obtained from the *in vitro* phase of the research and revealed that compounds **5**, **6**, and **7** exhibited the strongest binding to the 7AEM structure of EGFR, which represents the active kinase domain in complex with brigatinib. As EGFR activation triggers both PI3K/AKT and RAS-MAPK signaling cascades, it is likely that our compounds may affect both pathways by interfering with ATP binding at the kinase site. This hypothesis aligns with known mechanisms of EGFR tyrosine kinase inhibitors and their impact on downstream signaling in EGFR-positive tumors [65]. EGFR is commonly overexpressed in many types of human breast cancer, which indicates that an inhibitory effect on this factor’s expression and action is desirable and can explain the cytotoxic effect on MDA-MB-231 cancer cells [58]. Among all analyzed structures, molecule **5** was identified as the most promising candidate. It demonstrated the most similar binding affinity to the native ligand, which represents well-known antagonists of the EGFR. In contrast, the least energetically stable complex was observed for compound **6**, which did not exhibit cytotoxic activity against the MDA-MB-231 cancer cell line. This effect was instead noted in SW620 cancer cells, which have low EGFR expression, making therapies targeting this receptor largely ineffective [66].

#### 3.4.2 ADMET

Computer science and its various *in silico* models are increasingly used in the drug design process, assisting in the evaluation of newly discovered compounds. The integration of traditional scientific knowledge with the high computational power of artificial intelligence (AI) models helps to reduce costs and risks associated with preclinical and clinical trials, while also accelerating the drug discovery process [67, 68].

To assess the potential of the newly obtained (2-imino-4-oxo-1,3-thiazolidin-5-yl)acetic acid derivatives, we utilized the new ADMET-AI [46] model to predict key drug-like parameters and compared them to those of the reference antitumor drug etoposide [69], using standard statistical tools.

As key drug-like parameters, we selected LogP, bioavailability, quantitative estimate of drug-likeness (QED), and topological polar surface area (TPSA):

- LogP: A measure of a compound’s hydrophobicity, reflecting its ability to cross cell membranes. It helps predict a drug’s permeability, influencing its absorption and distribution [70].
- Bioavailability: Refers to the proportion of the drug that reaches systemic circulation in an active form after administration. It's critical for balancing efficacy, safety, and patient compliance [71].
- Quantitative Estimate of Drug-likeness (QED): This metric quantifies how similar a compound is to known drugs by considering properties such as molecular weight, solubility, and lipophilicity, helping prioritize candidates during drug development [72]
- Topological Polar Surface Area (TPSA): A molecular descriptor that estimates a compound’s polarity, which in turn affects its absorption, distribution, and permeability through biological membranes [73].

After prediction, the obtained data were standardized for better visualization on the heatmap and submitted to hierarchical cluster analysis, displayed as a dendrogram. As depicted in Fig. (**14**), the studied compounds formed clusters based on the similarities in their predicted parameter values. The closer and lower the branches are in the hierarchical clustering, the greater the similarity between the compounds. Particularly interesting are the placements of compounds **6** and **7,** which tend to be closer to and connected with the reference drug etoposide, despite the higher level of the branch. The connection between compounds at higher levels of the branches in the cluster may suggest that they share similarities in major parameters, with some exceptions. Compounds **6** and **7** registered the same QED and TPSA scores, 0.95 and 1.0, respectively, compared to etoposide, with scores of 0.73 and 0.77. The LogP values predicted for derivatives **6** and **7** were at the levels of 0.31 and 0.37, respectively, compared to 0.54 for Etoposide (Fig. **15**). The most significant difference between the studied compounds and the reference drug was observed in bioavailability, with a score difference of 0.59, which may account for the height of the cluster branch.

The results of *in silico* studies suggest that compounds **6** and **7** may have potential drug-like properties, due to their similarities to the reference antitumor medicine, Etoposide, in key parameters crucial for drug design. Moreover, the high QED and TPSA scores further emphasize their drug-likeness, reflecting structural and chemical similarities to known medicines, along with potentially favorable surface polarity that could enhance solubility and permeability. The height of the cluster branch, representing dissimilarities between compounds and the reference drug, is primarily due to significant differences in bioavailability. Compounds **6** and **7** achieved the highest possible bioavailability scores, indicating potentially better administration and absorption in the human body, compared to etoposide.

## CONCLUSION

The present work has established a coherent structure-activity narrative for a new family of fifteen (2-imino-4-oxo-1,3-thiazolidin-5-yl)acetic-acid derivatives and has identified two particularly promising molecules, compounds **5** and **7,** as credible leads for the development of anticancer drugs. Their superiority was first demonstrated in cytotoxic screening, where both showed low, micromolar IC_50_ values and the highest selectivity indices (2.42 - 4.73) in the panel, and was then reinforced by mechanistic assays. Flow-cytometry analysis revealed that compound **7** imposes a G0/G1 blockade in the highly EGFR-positive MDA-MB-231 line, whereas compound **5** accumulates cells in G2/M. In both cases, the arrest was accompanied by a significant rise in the sub-G1 fraction, confirming a shift toward apoptosis. Consistent with these findings, MDA-MB-231 cells proved more vulnerable than SW620 cells, and compound **7** produced the strongest pro-apoptotic effect, driving 6.60 ± 0.69% of the breast-cancer population into late apoptosis or necrosis.

Biochemical assays complemented the cell cycle data. Compound **5** suppressed IL-6 secretion in MDA-MB-231 cultures by 76%, linking its antiproliferative activity to the reduction of pro-inflammatory, tumor-promoting signaling. At the molecular level, docking experiments showed that this compound forms an energetically favorable complex with the EGFR kinase domain, surpassing the native ligand in calculated binding affinity. The 100 ns molecular dynamics simulations further demonstrated that the complex remains conformationally stable throughout the trajectory. Compound **7** distinguished itself *in silico* by combining equally strong docking scores with excellent bioavailability and drug-likeness parameters, its physicochemical profile closely matching that of the clinical reference etoposide.

Despite these encouraging *in vitro* and *in silico* results, several limitations should be acknowledged. This preliminary study provides compelling data on synthesized derivatives, but more advanced testing is needed to fully assess their therapeutic value. First, a broader panel of cancer cell lines will be included in future *in vitro* tests, involving the most potent compounds **5** and **7**. Second, only two triazole hybrids were evaluated due to solubility constraints and reaction conditions limitations. Alternative synthetic strategies will be employed to expand this series. Building on these encouraging results, future work will focus on broadening the scope of biological evaluation, including ADMET characterization, *in vivo* validation, and synthetic refinement to fully explore the clinical promise of this thiazolidinone series. Combination strategies involving the lead compounds may further enhance their therapeutic relevance.

Taken together, these findings validate (2-imino-4-oxo-1,3-thiazolidin-5-yl)acetic acid as a privileged core for cytotoxic lead generation and indicate that strategic substitution with either a tryptamine moiety (compound **5**) or a 1,2,4-triazole ring (compound **7**) is particularly advantageous. Compared with earlier reports on related thiazolidinone scaffolds [74], the present derivatives exhibit improved potency, clearer selectivity, and mechanistic features consistent with EGFR inhibition and cytokine modulation. On this foundation, future studies will focus on (i) Broadening the chemical space around the tryptamine and triazole vectors to generate a second-generation library, (ii) Refining synthetic protocols, especially for the hybrid 4-thiazolidinone and 1,2,4-triazole framework to improve yield and facilitate late-stage diversification, and (iii) Extending the evaluation to pharmacokinetic profiling and *in vivo* efficacy models. By coupling iterative scaffold optimization with targeted kinase-panel screening and metabolic stability assays, we aim to produce thiazolidinone-based candidates exhibiting nanomolar potency, robust selectivity, and a safety profile suitable for preclinical advancement.

## Figures and Tables

**Fig. (1):**
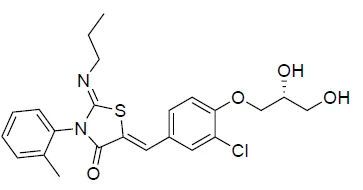
Chemical structure of Ponesimod containing 4-thiazolidinone ring.

**Fig. (2):**
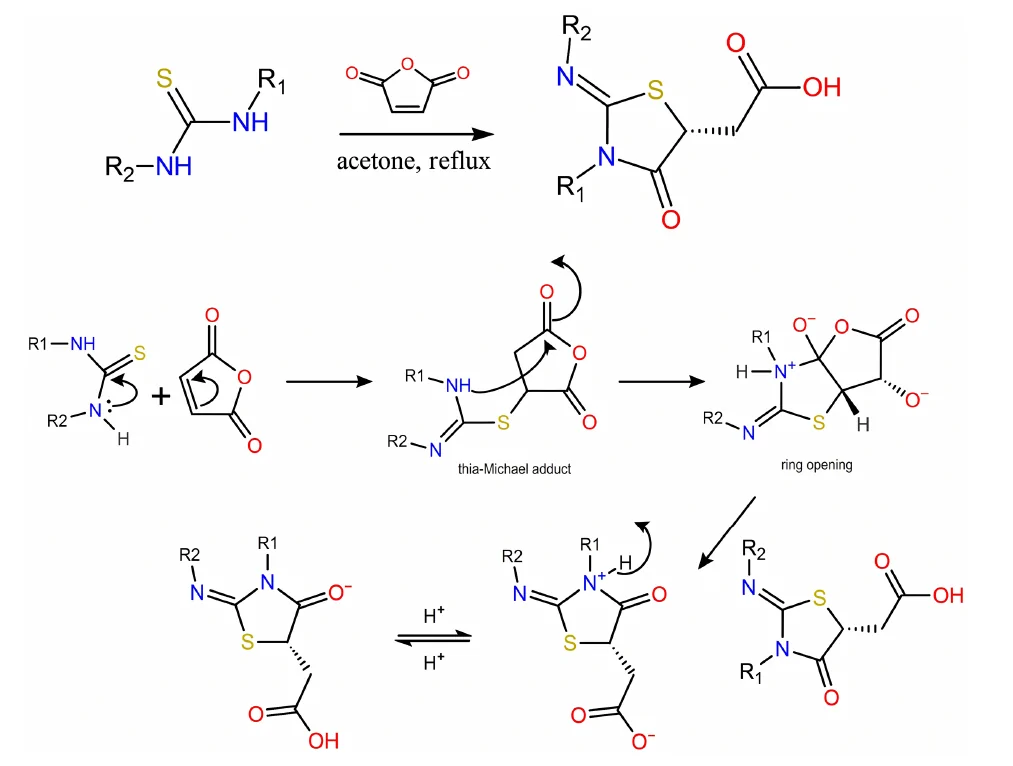
The scheme of the synthetic pathway of new (2-imino-4-oxo-1,3-thiazolidin-5-yl)acetic acid derivatives (compounds **1**-**15**) with proposed general mechanism of product formation.

**Fig. (3):**
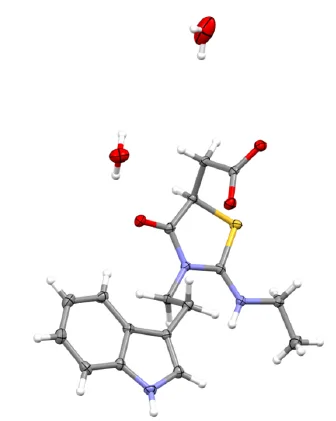
The molecular structure of compound **2** with two water molecules showing displacement ellipsoids at the 50% probability level.

**Fig. (4):**
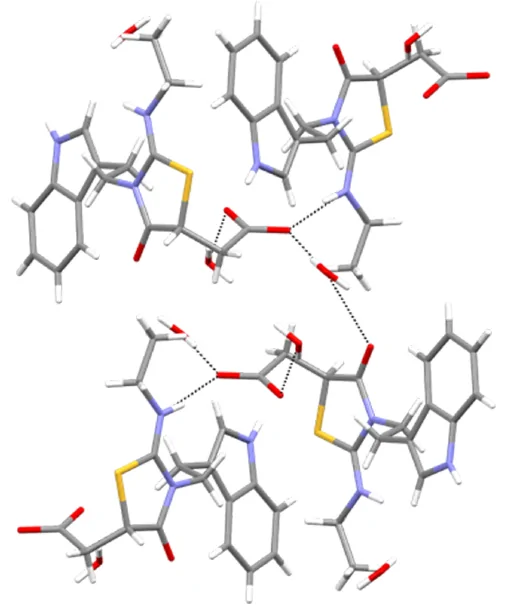
Packing arrangement and key intermolecular interactions within the crystal lattice of compound 2, viewed along the [100] crystallographic axis.

**Fig. (5):**
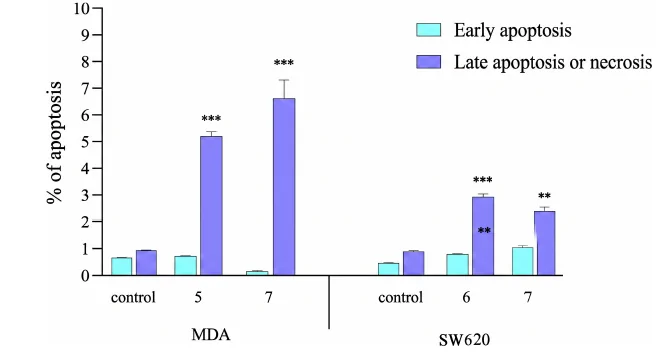
Effect of novel N-derivatives of (2-imino-4-oxo-1,3-thiazolidin-5-yl)acetic acid **5**, **6,** and **7** on late apoptosis or necrosis and early apoptosis in human metastatic colon cancer (SW620) and human breast adenocarcinoma (MDA-MB-231) cell lines. Cells were treated with compounds at their IC_50_ values and incubated for 72h, followed by staining with annexin V-FITC and propidium iodide (PI). The assay was performed using flow cytometry. The outcomes are expressed as the proportion of cells undergoing early apoptosis and those in late apoptosis or necrotic stages. *** *p* ≤ 0.0001, ** *p* ≤ 0.001, in comparison to the control group.

**Fig. (6):**
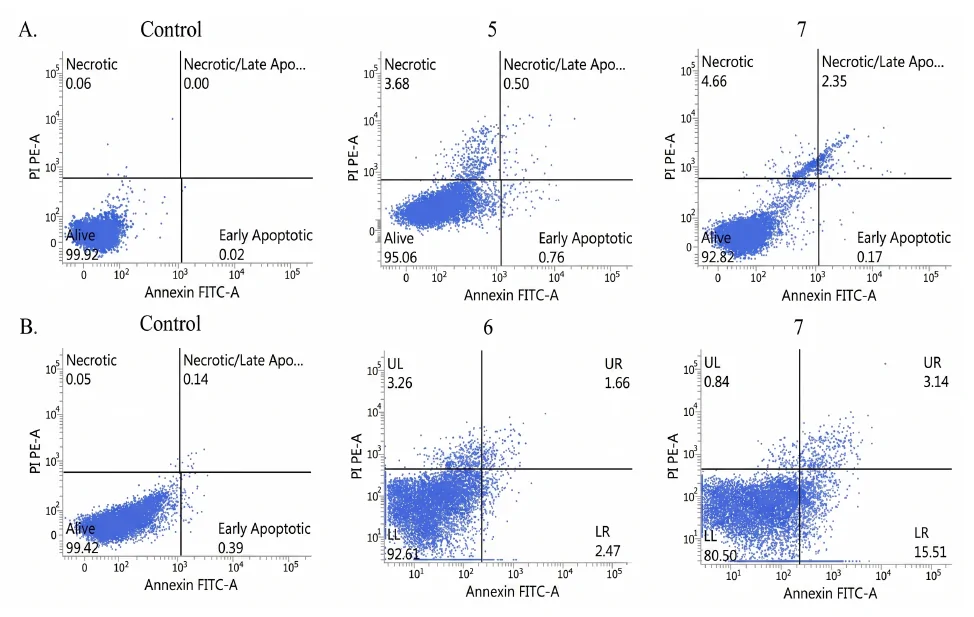
Representative percentages presented as dot plots illustrate the apoptosis assessment of (**A**) MDA-MB-231, (**B**) SW620, and other cancer cell lines treated with compounds **5**, **6**, and **7**. The analysis was carried out *via* flow cytometry using Annexin V-FITC/PI double staining, distinguishing between Q1-viable cells, Q2-early apoptotic, Q3-late apoptotic/necrotic, and Q4-necrotic populations.

**Fig. (7):**
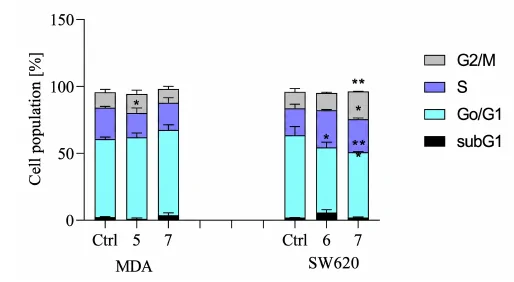
Cell cycle analysis of MDA-MB-231 and SW620 cells was performed after 24-hour treatment with compounds 5, 6, and 7 at concentrations equivalent to their respective IC_50_ values. The SubG1 population corresponds to apoptotic and non-viable cells. Data are shown as mean ± standard deviation (SD) from three independent experiments. ** *p* ≤ 0.001, * *p* ≤ 0.05, compared to the untreated control.

**Fig. (8):**
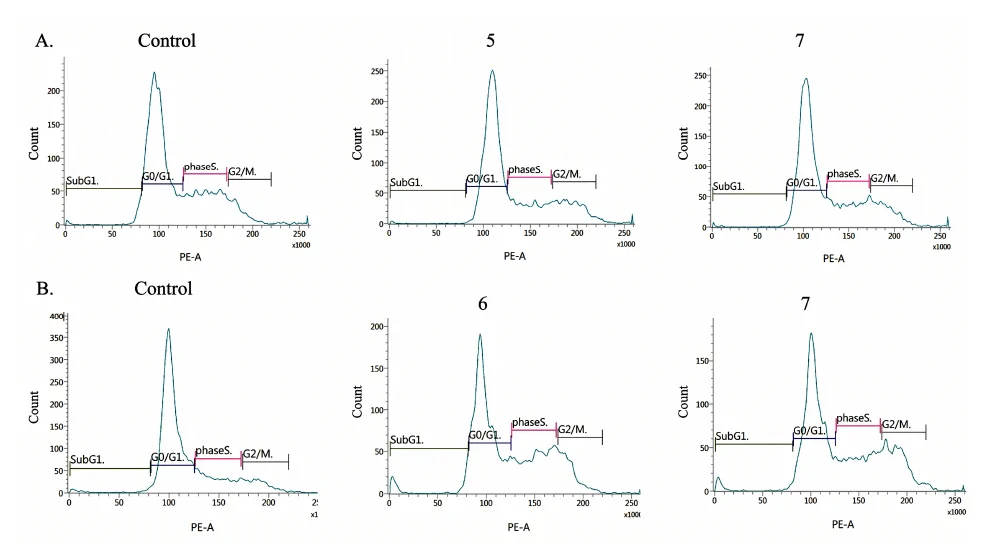
Flow cytometric evaluation of cell cycle distribution was conducted following 24-hour exposure of (**A**) MDA and (**B**) SW620 cancer cells to compounds 5, 6, and 7 at concentrations corresponding to their IC_50_ values. The SubG1 phase indicates the proportion of apoptotic or non-viable cells. Data are expressed as mean ± standard deviation (SD) from three independent experiments.

**Fig. (9):**
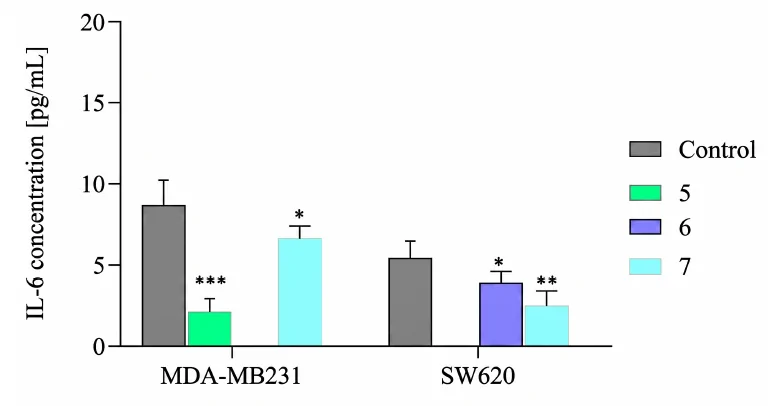
The influence of novel (2-imino-4-oxo-1,3-thiazolidin-5-yl)acetic acid derivatives 5, 6, and 7 on IL-6 secretion was evaluated using ELISA in supernatants collected from human metastatic colon cancer (SW620) and human breast adenocarcinoma (MDA-MB-231) cell cultures. Results are presented as mean ± standard deviation (SD) from three independent experiments, each performed in triplicate. *** *p* ≤ 0.001, ** *p* ≤ 0.01, * *p* ≤ 0.05, relative to the control group.

**Fig. (10):**
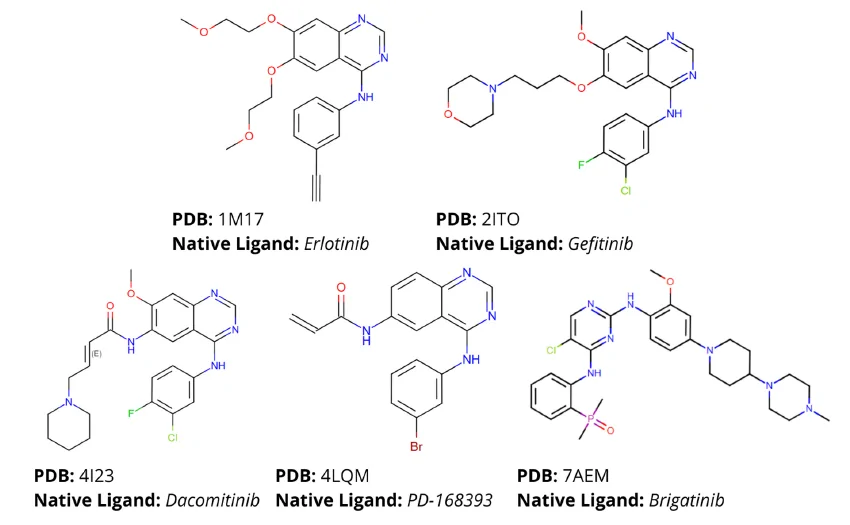
Native ligands from RCSB protein data bank EGFR complexes.

**Fig. (11):**
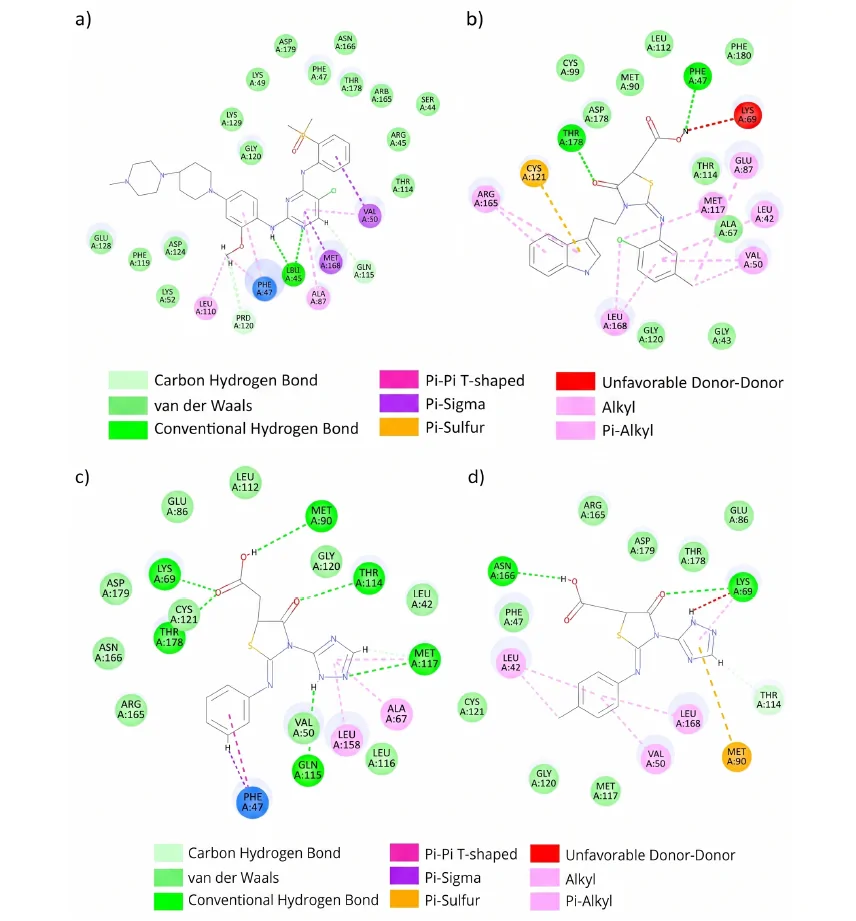
A visualization of intermolecular interactions for EGFR protein complexes with: (**a**) the native molecule, (**b**) compound **5**, (**c**) compound **6**, and (**d**) compound **7**.

**Fig. (12):**
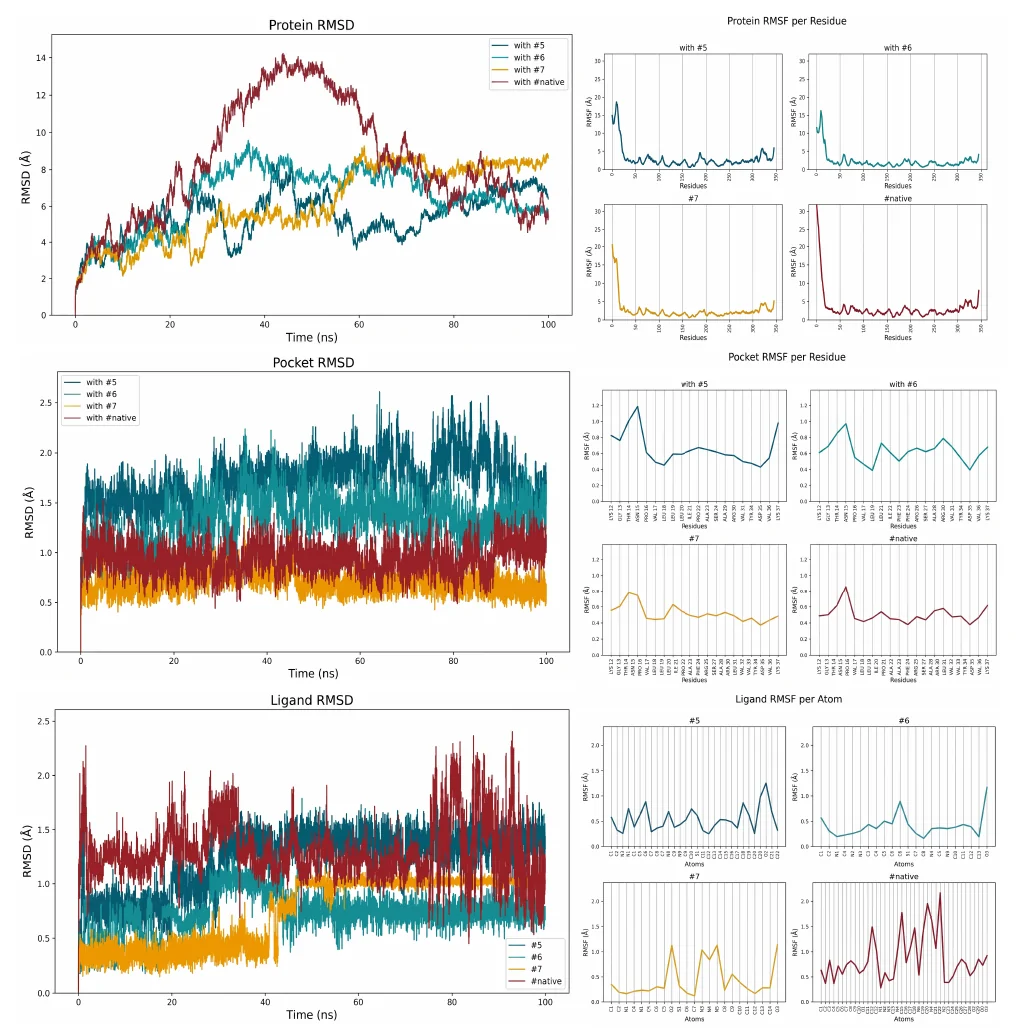
On the left, from top to bottom, are comparisons of RMSD for proteins, pockets, and ligands derived from Molecular Dynamics simulations. On the right are RMSF plots, corresponding respectively to protein amino acids, pocket amino acids, and ligand atoms.

**Fig. (13):**
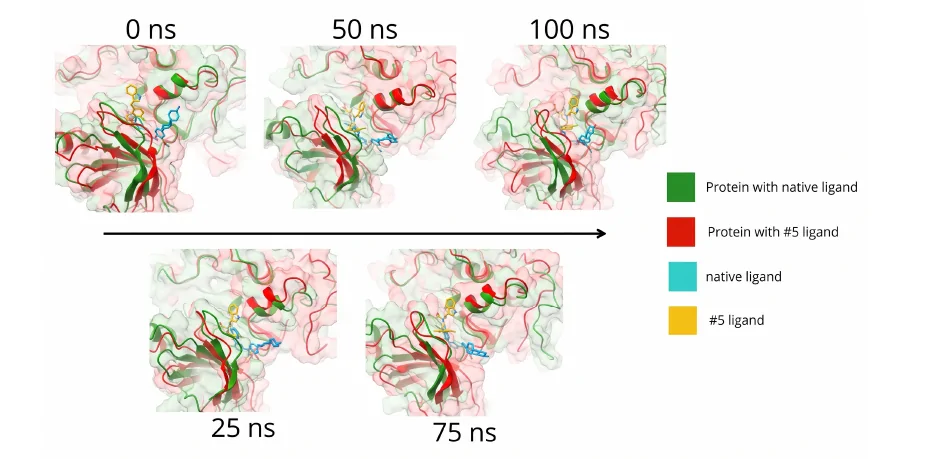
A visualization comparing the shape of the protein-ligand complex between the native protein-molecule complex and the protein-molecule 5 complex at 0 ns, 25 ns, 50 ns, 75 ns, and 100ns.

**Fig. (14):**
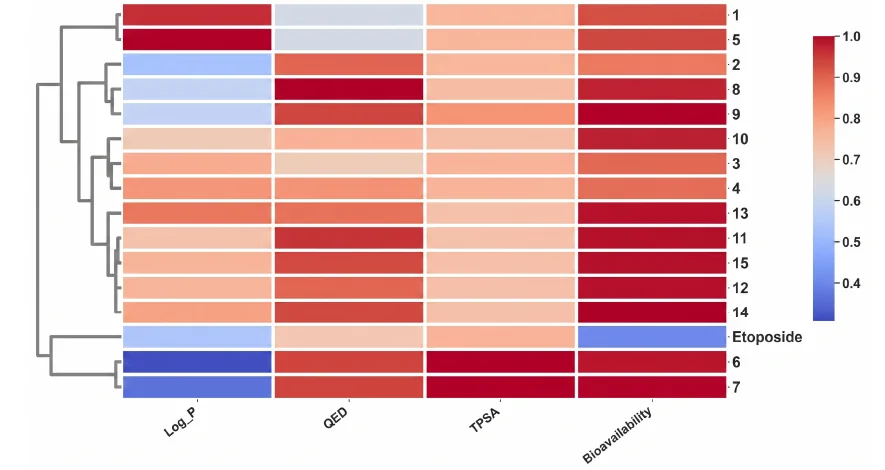
A heatmap chart with hierarchical cluster analysis of predicted and standardized key drug-like parameters- logP, bioavailability, Quantitative Estimate of Drug-likeness (QED), and Topological Polar Surface Area (TPSA), for all novel (2-imino-4-oxo**-**1,3-thiazolidin-5-yl)acetic acid derivatives, compared to the reference drug Etoposide, and performed by ADMET-AI model.

**Fig. (15):**
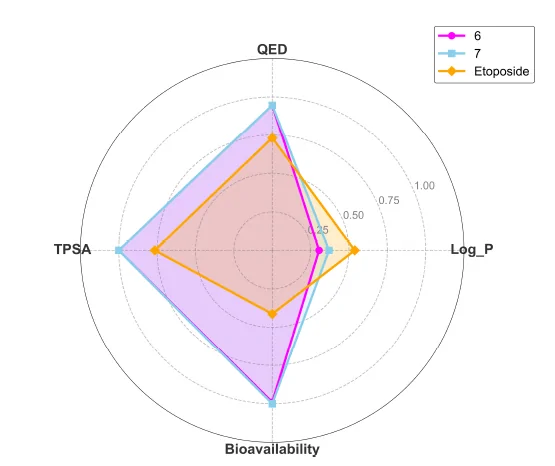
A radar chart of compounds **6** and **7**, comparing predicted and standardized values of QED, logP, bioavailability, and TPSA to the reference drug Etoposide.

**Table 1:** Summary of parameters used in molecular dynamics simulations.

**Properties**	**Molecular Dynamics Simulation Step**					-
	**Minimization**	**Heating**	**Backbone Restrains Removal**	**NVT Equilibration**	**NPT Equilibration**	**Production Run**
Time [ns]	~ until 10*kj/mole	0.025	0.5	0.5	0.5	100
Temperature	50	from 50 to 300	300	300	300	300
Integrator	Langevin Integrator					
Thermostat	Andersen Thermostat					
Barostat	MonteCarlo Barostat					
Timestep Value [ps]	1.0					
Friction Value [1/ps]	1.0					
NonBonded Method	Periodic Mesh Ewald with 10.0Å cutoff					

**Table 2:** Summary of main heterocyclic substituent (R_1_) and additional substituents (R_2_) applied for the synthesis of new (2-imino-4-oxo-1,3-thiazolidin-5-yl)acetic acid derivatives (compounds 1-15).

**R_1_**	**R_2_**	
Tryptamine	1(-3Cl,4F-Ph) 2 (-Et), 3 (-Ph)	4 (-cyclohexyl) 5 (-2Cl,4Me-Ph)
1,2,4-Triazole	6 (-Ph) 7 (-4Me-Ph)	
Thiazole	8 (-Ph) 9 (-OMe-Ph) 10 (-4I-Ph) 11 (-2Cl-Ph)	12 (-4Br-Ph) 13 (-3,4Cl-Ph) 14 (-3Me,4Cl-Ph) 15 (-3F,4Cl-Ph)

**Table 3:** Crystallographic parameters and structure refinement details for compound 2.

**Properties**	**Compound 2**
Empirical formula	C_17_H_19_N_3_O_3_S_1_,2(H_2_O)
Formula weight	381.44
Temperature [K]	100.00(10)
Space group	P2_1_/c
Unit cell dimensions	-
*a* [Å]	7.4109(2)
*b* [Å]	12.5547(4)
*c* [Å]	19.1743(7)
a [°]	90.00
β [°]	100.129(3)
γ [°]	90.00
Volume *V* [Å^3^]	1756.21(10)
Z [molecules/cell]	4
*D*_calculated_ [g cm^-3^]	1.443
Absorption coefficient μ/mm^-1^	0.219
*2θ* range for data collection [°]	3.9-52.72
Limiting indices	-6 < = *h* = > 9
-	-12 < = *k* = > 15
-	-20 < = *l* = > 23
Reflections collected/unique	6615/3594
Data/parameters	3594/254
Goodness of Fit	1.041
Final *R* index (*I* > 2σ)	0.0543
w*R*^2^	0.1334
Largest diff. Peak and hole [Å^-3^]	0.90 and -0.60

**Table 4:** Assessment of cell viability reduction by the studied compounds *via* MTT^a^ assay.

**Compound**	**Cancer Cell Lines**				**Normal Cells**
	**MDA^f^**		**SW 620^d^**		**HaCaT^e^**
	**IC_50_^b^**	**SI^c^**	**IC_50_^b^**	**SI^c^**	**IC_50_^b^**
1	100	1	100	1	>100
2	100	1	100	1	>100
3	100	1	100	1	>100
4	100	1	100	1	>100
5	21.16 ± 0.36	4.73	100	1	>100
6	100	1	41.31 ± 1.98	2.42	>100
7	36.82 ± 2.78	2.71	24.10 ± 3.98	4.16	>100
8	100	1	100	1	>100
9	100	1	100	1	>100
10	77.18 ± 3.68	1.30	100	1	>100
11	100	1	100	1	>100
12	100	1	100	1	>100
13	100	0.24	75.71 ± 8.06	0.31	23.48 ± 7.45
14	100	1	100	1	>100
15	100	1	53.24 ± 2.80	1.88	>100
Ref^g^	1.65±0.23	0.28	1.83 ± 0.10	0.16	0.46 ± 0.03

**Note:** ^a^ The MTT assay is a commonly applied colorimetric technique to evaluate cell viability based on metabolic activity. It functions through NADPH-dependent oxidoreductase enzymes that convert the MTT tetrazolium salt into an insoluble, purple-colored formazan product. ^b^ Data are reported as mean ± SD. The IC_50_ (µM) value indicates the compound concentration that reduces cell growth by 50% in comparison to untreated control cells, following 72 hours of exposure. ^c^ The Selectivity Index (SI) was determined using the equation: SI = IC_50_ for non-cancerous cells / IC_50_ for cancerous cells. ^d^ Human metastatic colon cancer cell line (SW620). ^e^ Human keratinocyte cell line derived from adult skin (HaCaT). ^f^ Human breast adenocarcinoma cells (MDA). ^g^ Doxorubicin was used as a standard reference drug due to its established role in anticancer therapy.

**Table 5:** Comparison of average AutoDock SMINA results for native and studied molecules.

**Compound**	**Crystal ID**				
-	**1M17**	**2ITO**	**4I23**	**4LQM**	**7AEM**
-	Predicted Binding Score [kcal/mol] (↓)				
Native Molecule	-6.74	-7.34	-7.83	-1.51	-7.71
-	10 Poses Predicted Binding Score (min|max) [kcal/mol]				
**5**	-8.7|-7.5	-8.8|-8.1	-8.4|-7.6	-8.7|-7.9	**-9.4**|-8.0
**6**	-7.6|-6.9	-7.6|-7.0	-8.6|-7.3	-8.4|-7.5	-8.7|-7.5
**7**	-8.4|-7.8	-8.1|-7.4	-8.7|-7.2	-8.4|-7.8	**-9.2**|-8.0

**Table 6:** Root mean square deviation (RMSD) of ligand, protein, and pocket in angstrom.

-	-	-	**Compound**	-
-	**native**	**5**	**6**	**7**
Ligand RMSD (mean|std) [Å]	1.29 | 0.27	1.22 | 0.28	0.73 | 0.18	0.74 | 0.31
Protein RMSD (mean|std) [Å]	8.41 | 3.14	5.45 | 1.3	6.47 | 1.70	6.13 | 2.06
Pocket RMSD (mean|std) [Å]	0.94 | 0.15	1.72 | 0.24	1.32 | 0.24	0.70 | 0.11

**Table 7:** ΔMM/PBSA and ΔMM/GBSA total energy comparison between native and studied molecules.

-	-	-	**Compound**	-
-	**Native**	**5**	**6**	**7**
ΔMM/PBSA	-34.42 | 0.82	94.22 | 3.40	1584.83 | 149.11	562.43 | 84.10
ΔMM/GBSA	-41.84 | 0.38	72.15 | 3.20	1554.31 | 149.25	536.40 | 84.36

## Data Availability

Detailed experimental data supporting the findings of this study are available upon request.

## LIST OF ABBREVIATIONS

ADME: Absorption, Distribution, Metabolism, and Excretion
ADMET: Absorption, Distribution, Metabolism, Excretion and Toxicity
AI: Artificial Intelligence
ATP: Adenosine-5′-triphosphate
COX-2: Cyclo-oxygenase-2
DFT: Density-functional Theory
DMSO: Dimethyl Sulfoxide
EGFR: Epidermal Growth-factor Receptor
ELISA: Enzyme-linked Immunosorbent Assay
ESI: Electrospray Ionisation
FACS: Fluorescence-activated Cell Sorting
FITC: Fluorescein Isothiocyanate
GBSA: Generalized Born Surface-area (energy)
GI%: Growth-inhibition Percentage
HRMS: High-resolution Mass Spectrometry
IC_50_: Half-maximal Inhibitory Concentration
IL-6: interleukin-6
LogP: Logarithm of the n-octanol/Water Partition Coefficient
MAPK: Mitogen-activated Protein Kinase
MD: Molecular Dynamics
MM/GBSA: Molecular-mechanics/Generalised Born Surface-area (energy)
MM/PBSA: Molecular-mechanics/Poisson- Boltzmann Surface-area (energy)
MTT: 3-(4,5-dimethylthiazol-2-yl)-2, 5-diphenyltetrazolium Bromide
NMR: Nuclear-magnetic Resonance
PBS: Phosphate-buffered Saline
PI: Propidium Iodide
PIM: Proviral-integration-site for Moloney-murine-leukemia Virus Kinase
PI3K: Phosphatidylinositol-3-kinase
QED: Quantitative Estimate of Drug-likeness
RAS: Rat Sarcoma (GTPase)
RMSD: Root-mean-square Deviation
RMSF: Root-mean-square Fluctuation
ROS: Reactive Oxygen Species
SI: Selectivity Index
STAT3: Signal Transducer and Activator of Transcription 3
TPSA: Topological Polar Surface Area
VEGFR: Vascular Endothelial Growth- factor receptor
5-LOX: 5-lipoxygenase
