# Supplementary material for: Synthesis, Cytotoxicity Evaluation, and Molecular Dynamics of 4-Thiazolidinone Derivatives: A Potential Path to EGFR-Targeted Anticancer Therapy
Source: Curr Med Chem. 2025 Oct 10;33(25):5268–96. doi: 10.2174/0109298673400295250913044755 (PMC13692766; doi:10.2174/0109298673400295250913044755)
Supplement: Supplementary file 1 [file CMC-33-25-5268_SD1.pdf]

Supplementary Material

Synthesis, Cytotoxicity Evaluation, and Molecular Dynamics of 4-Thiazolidinone Derivatives: A Potential Path to EGFR-Targeted Anticancer Therapy

Tomasz Szostek[1], Dagmara Otto-Ślusarczyk[1], Michał A. Dobrowolski[2], Maciej Wiśniewski[3,4], Piotr Roszkowski[2] and Daniel Szulczyk[1,*]

[1]Chair and Department of Biochemistry, Medical University of Warsaw, 02-097 Warsaw, Poland; [2]Faculty of Chemistry, University of Warsaw, Pasteura 1, 02-093 Warsaw, Poland; [3]Laboratory of Bioinformatics and Computational Genomics, Faculty of Mathematics and Information Science, Warsaw, Poland; [4]Laboratory of Functional and Structural Genomics, Center of New Technologies, University of Warsaw, Warsaw, Poland

S.2.1 Chemistry

Data availability statement

Part of the datasets generated and analyzed during the current study are not publicly available due to requests from co-authors (possible patent submission), but are available from the corresponding author on reasonable request.

Below are examples of the obtained derivatives' NMR spectra. More data was collected and can be shared through a reasonable request to the corresponding author.

Figure S1. ^{1}H NMR of **compound 1.**

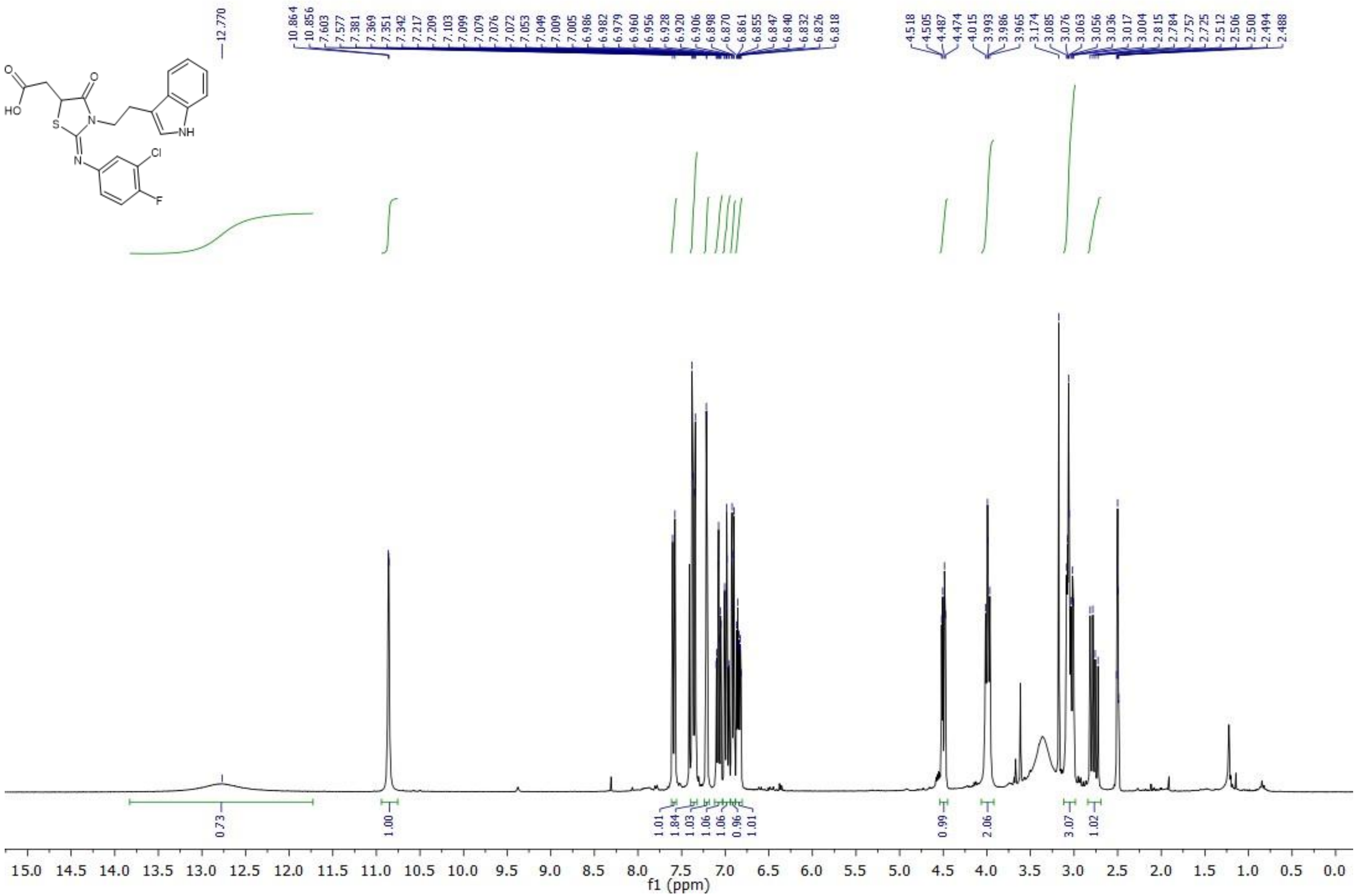

Figure S2. ^{13}C NMR of compound **1.**

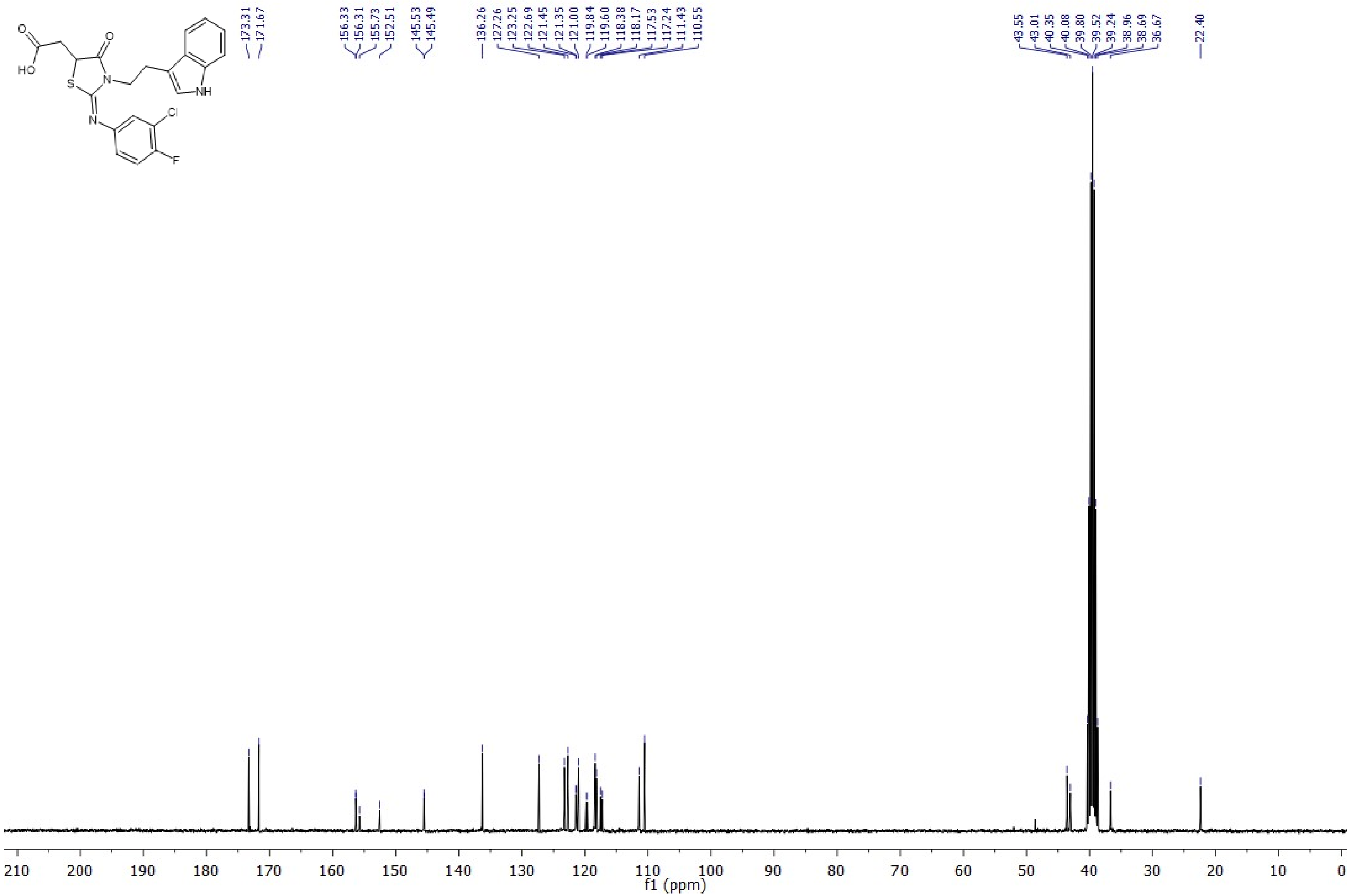

Figure S3. ^{1}H NMR of compound **2.**

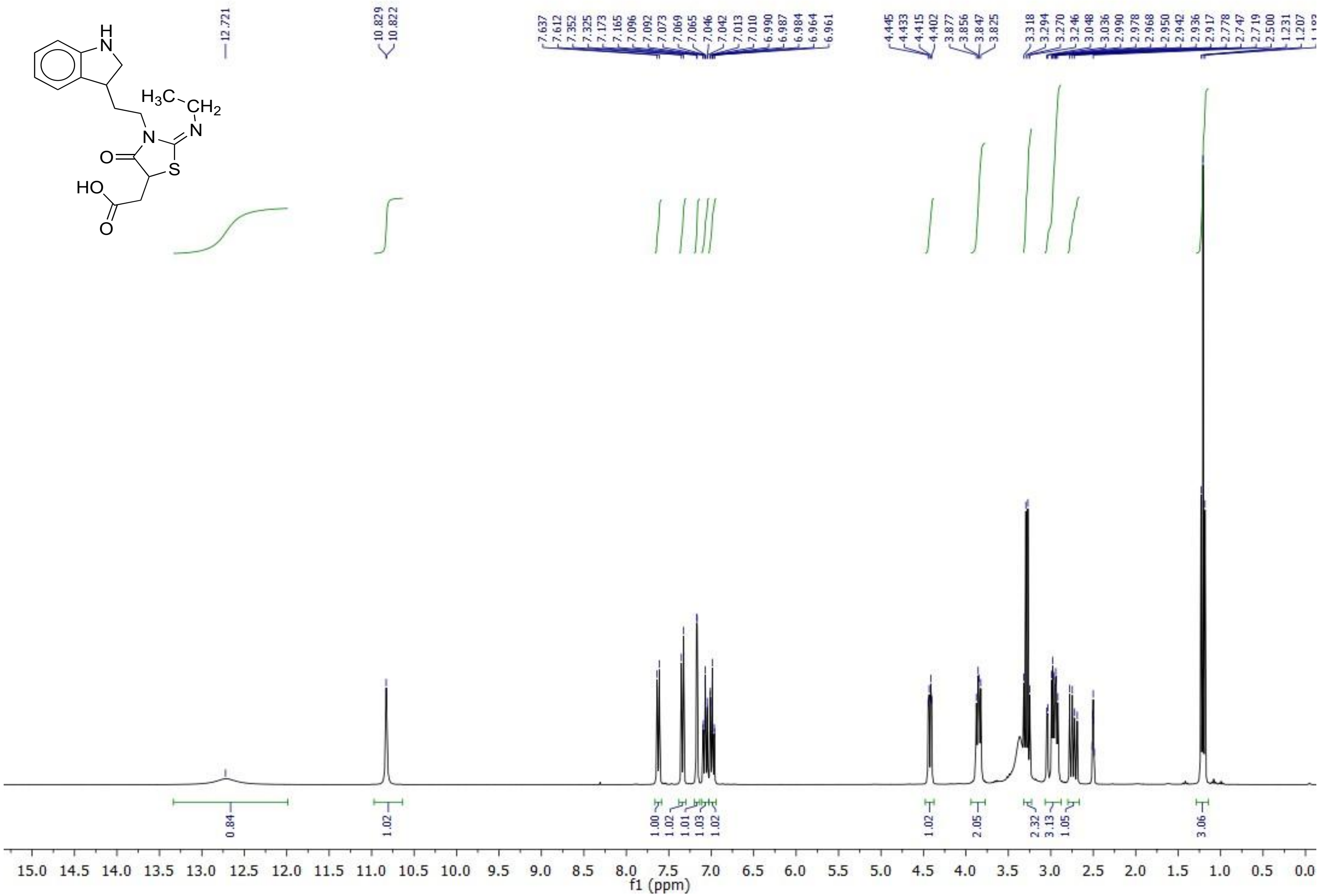

Figure S4. ^{13}C NMR of compound **2.**

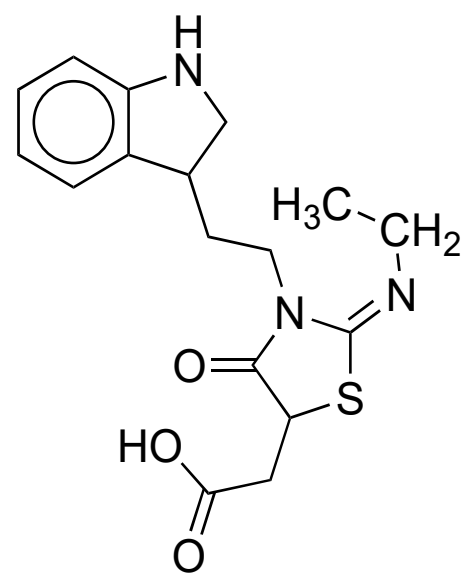

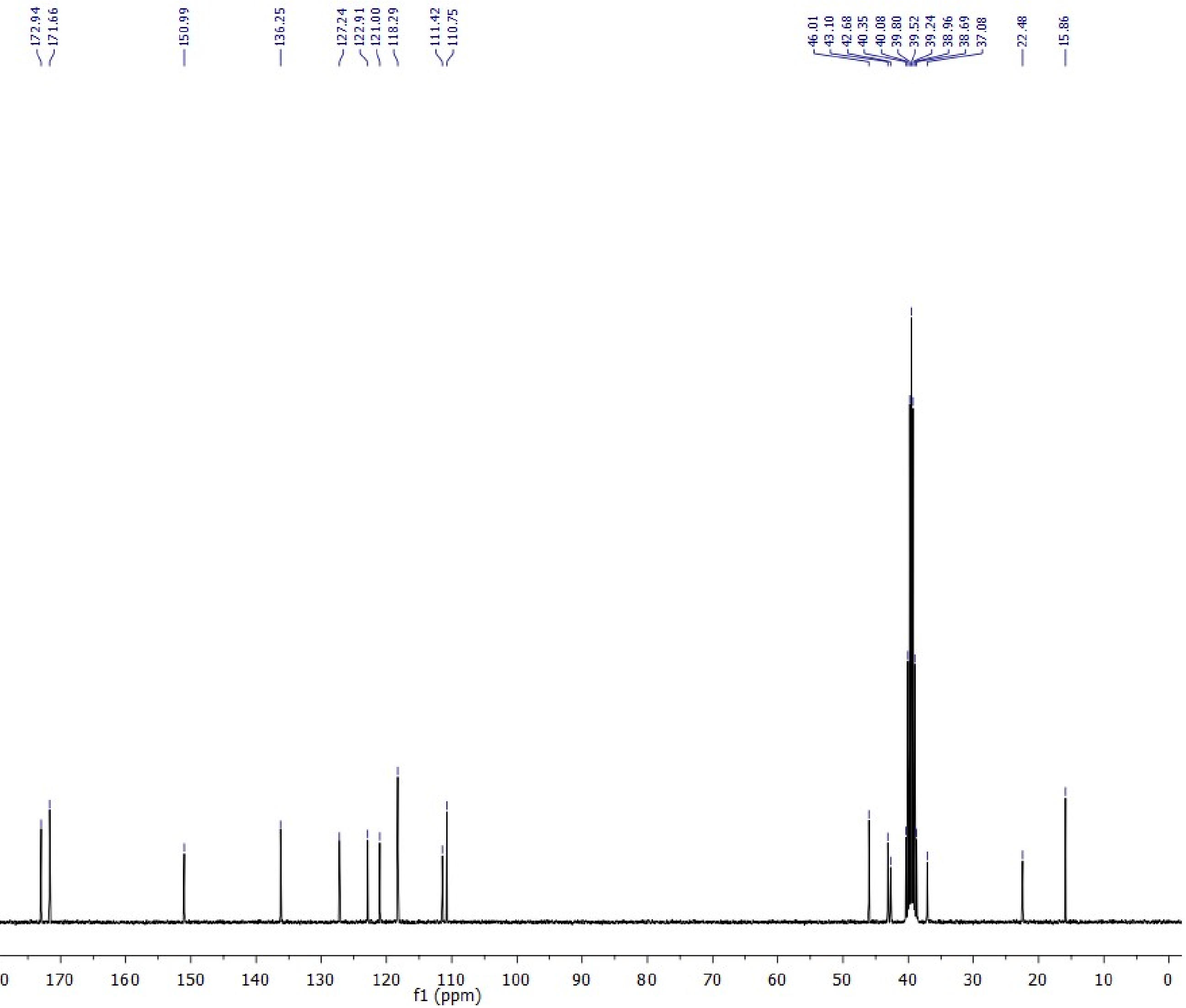

Figure S5. ^{1}H NMR of compound **3.**

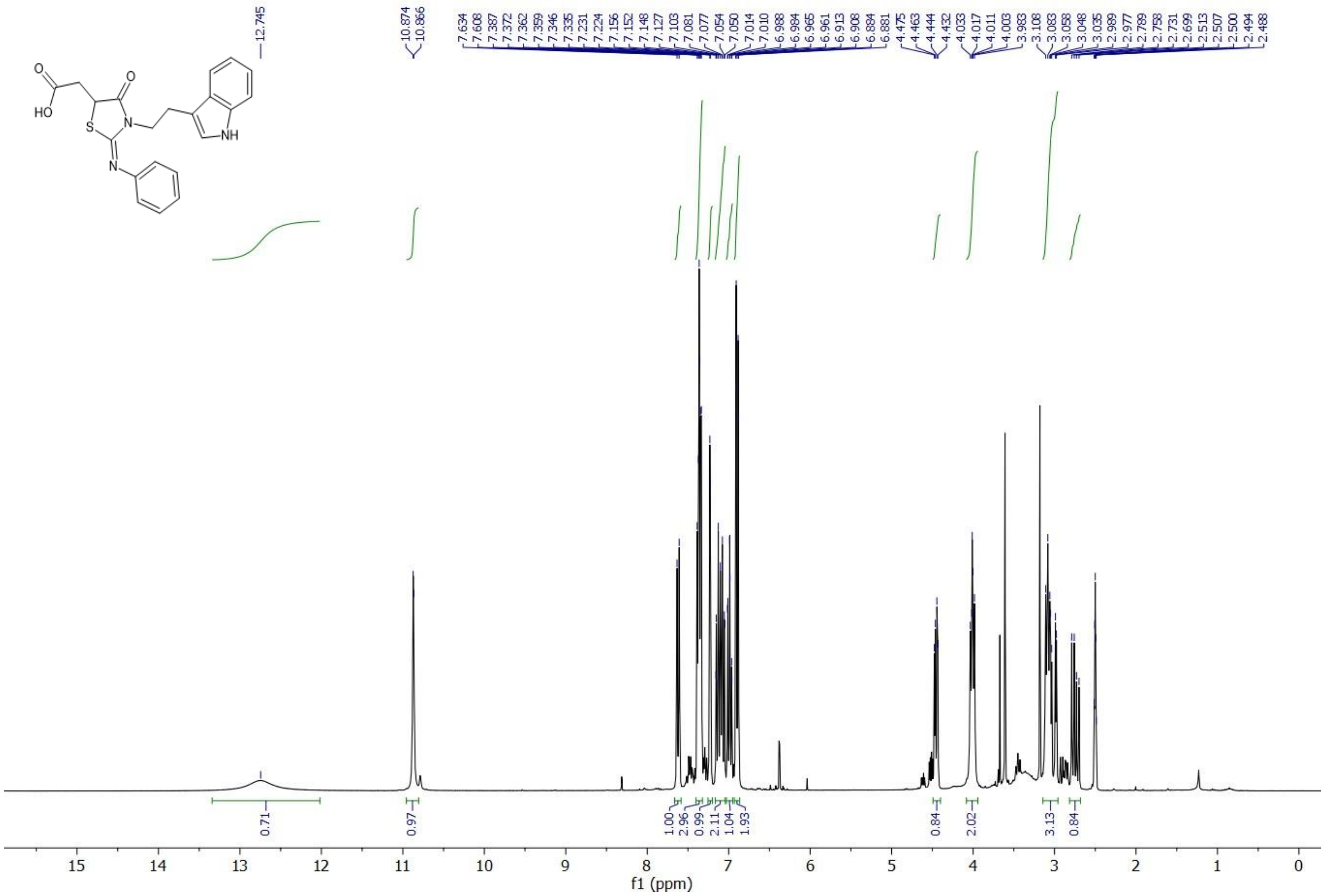

Figure S6. ^{13}C NMR of compound **3.**

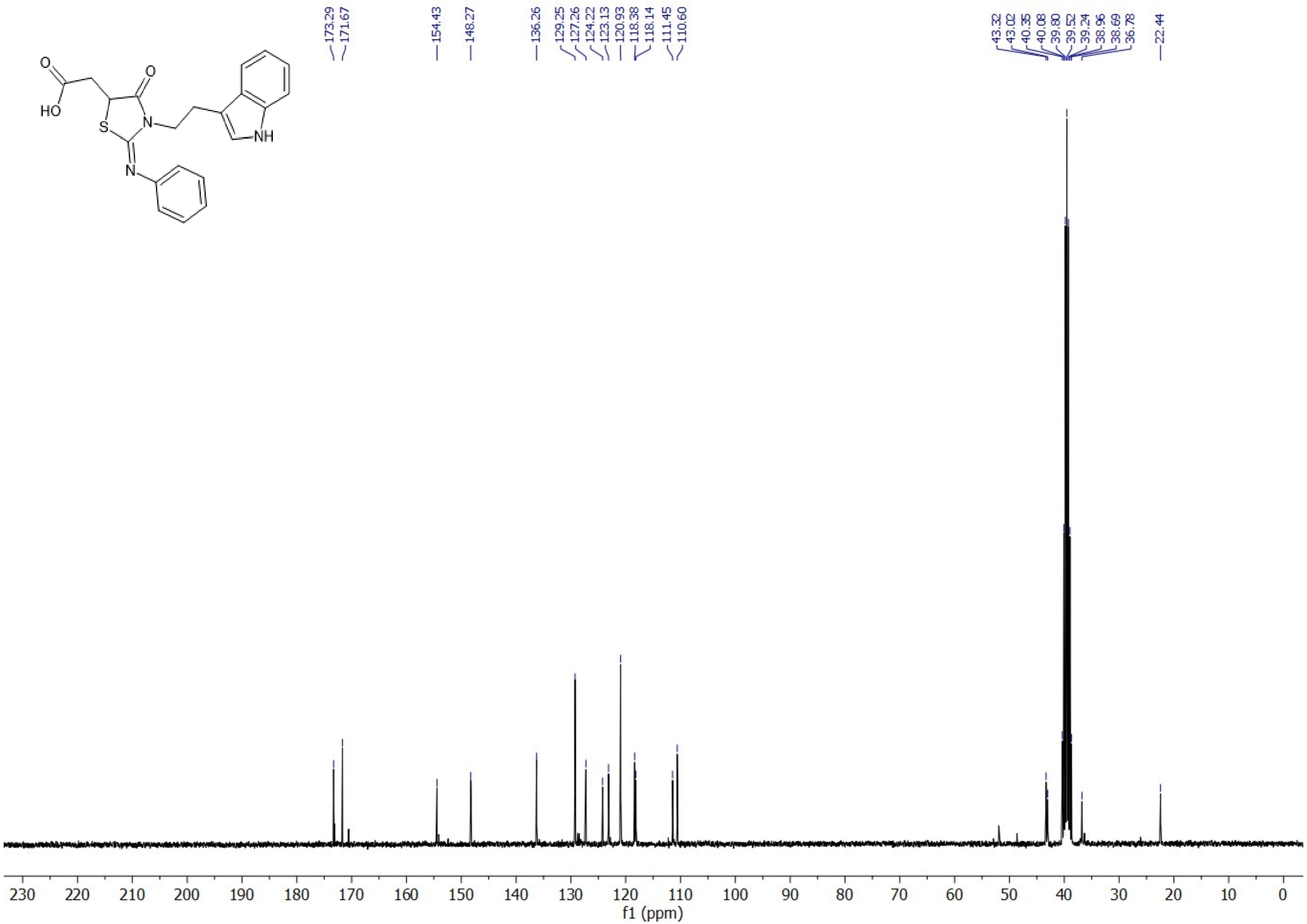

Figure S7.^{1}H NMR of compound **4.**

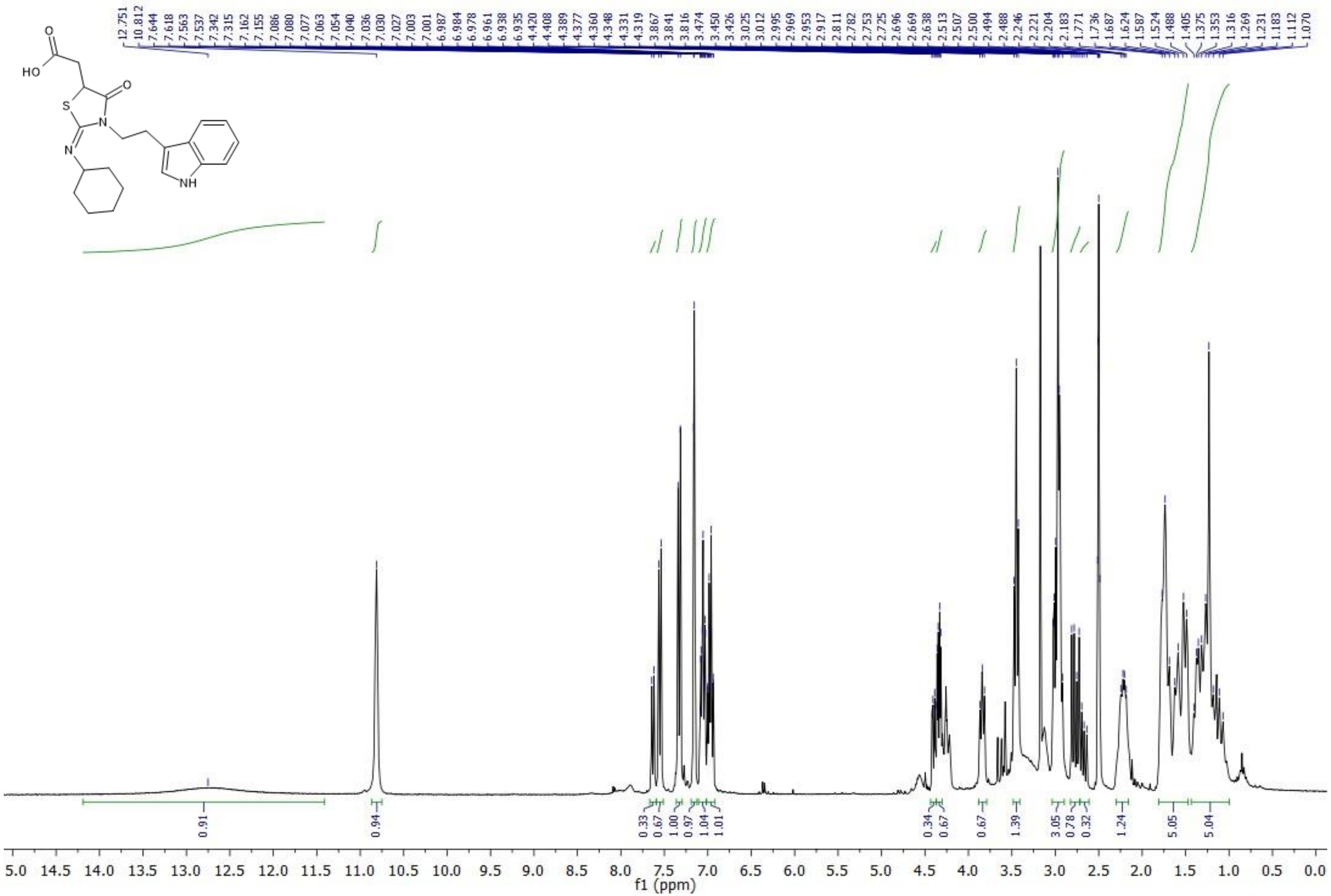

Figure S8. ^{13}C NMR of compound **4.**

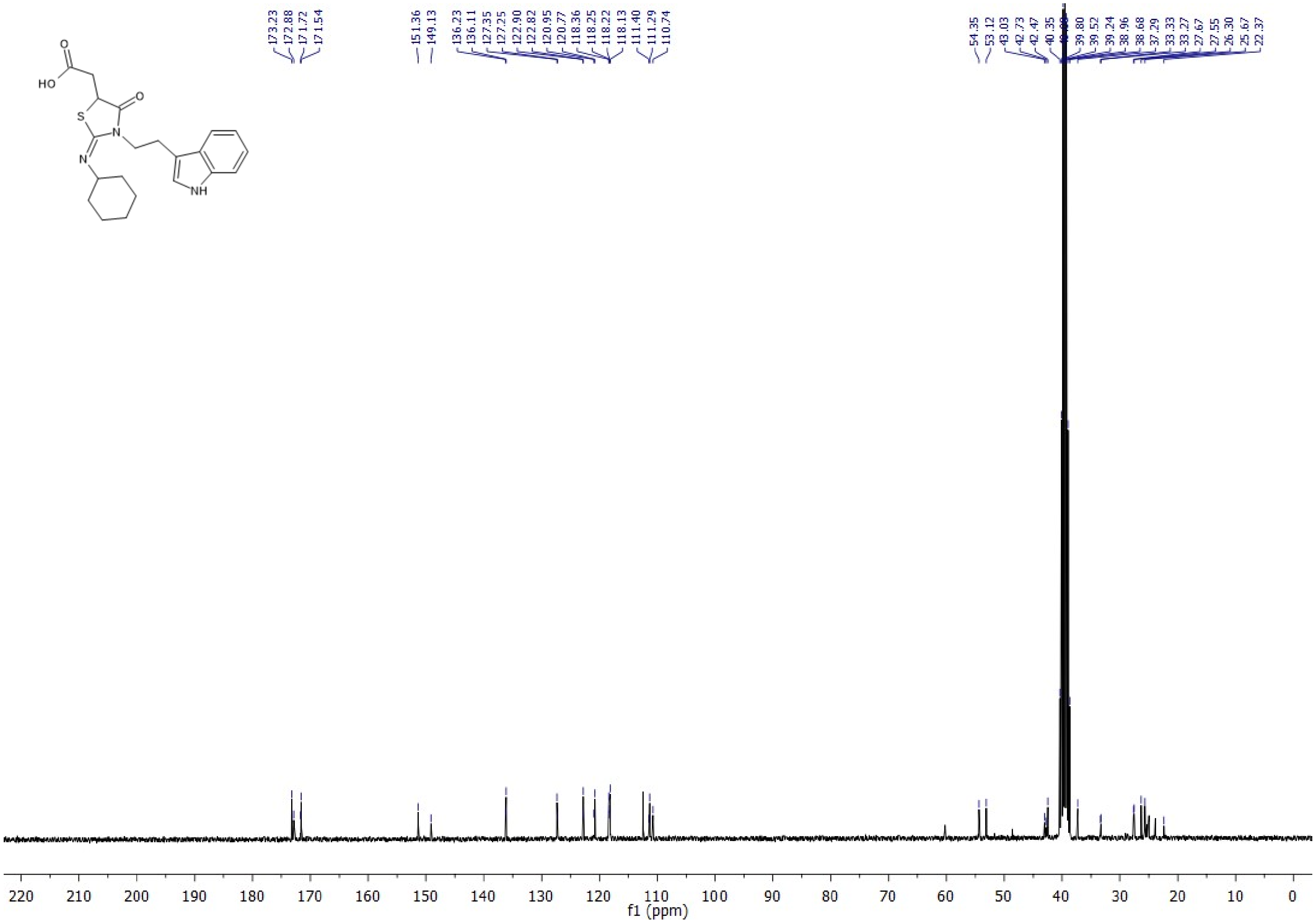

Figure S9. ^{1}H NMR of compound **5.**

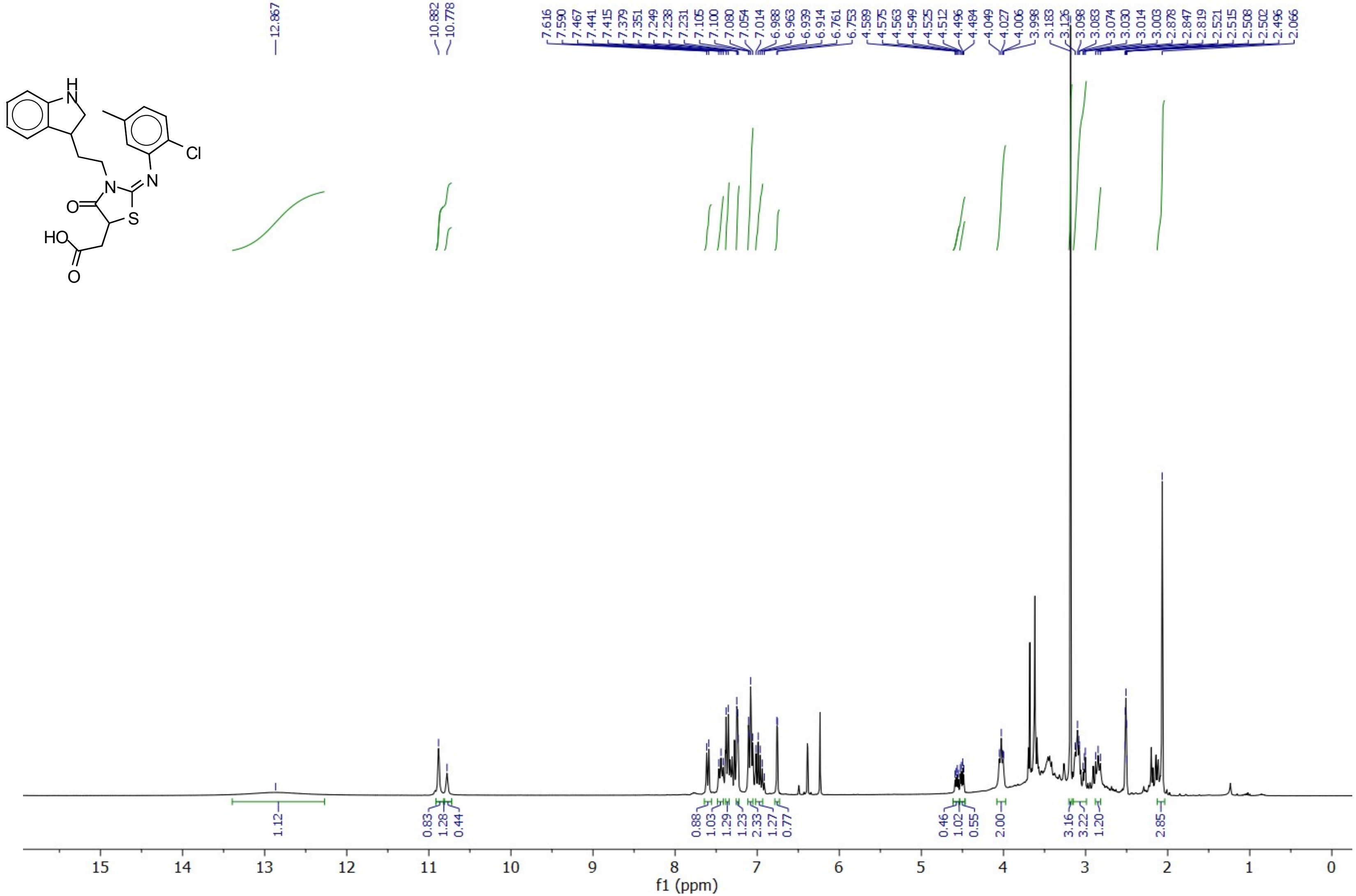

Figure S10. ^{13}C NMR of compound **5.**

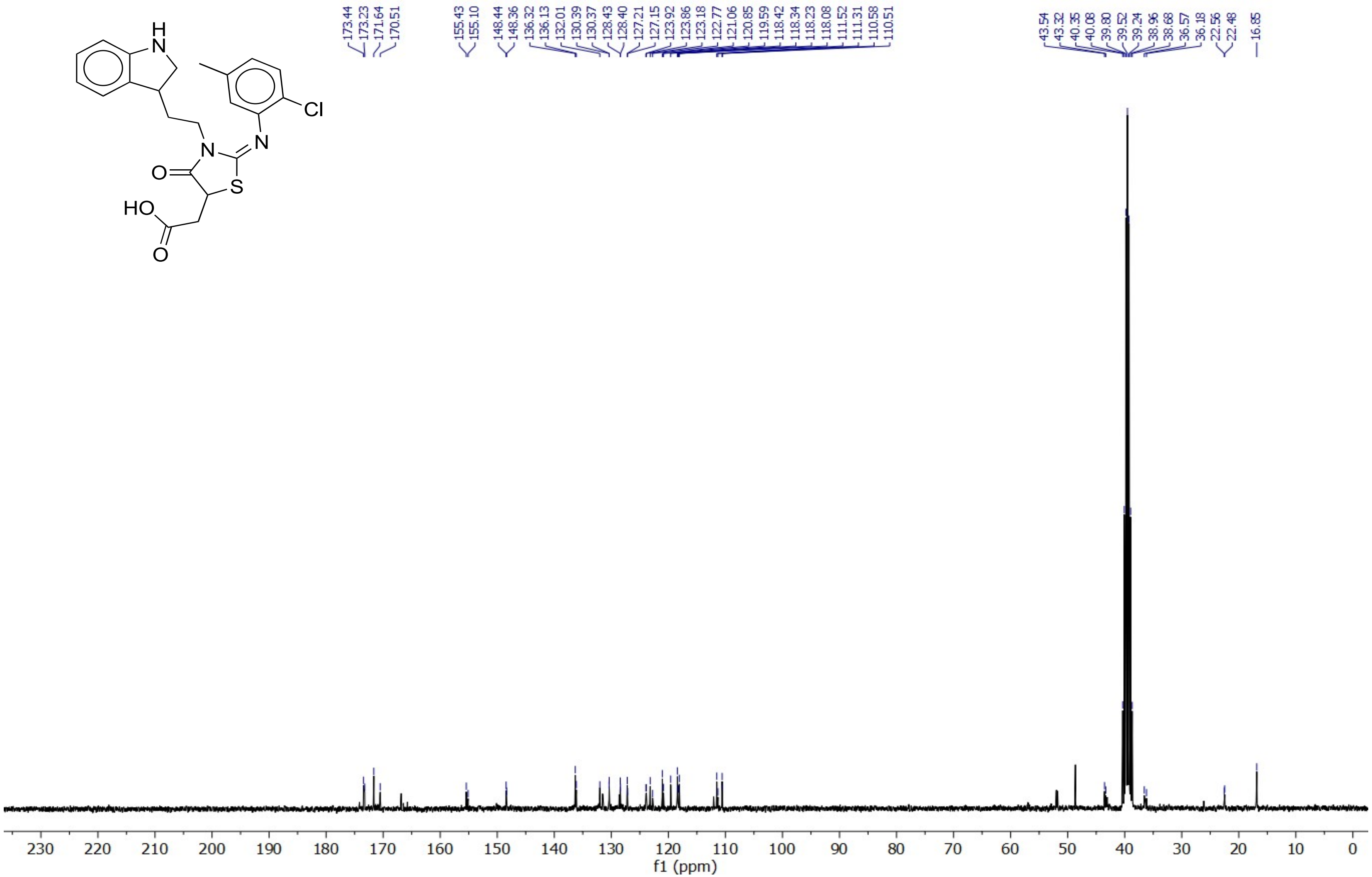

Figure S11. ^{1}H NMR of compound **6.**

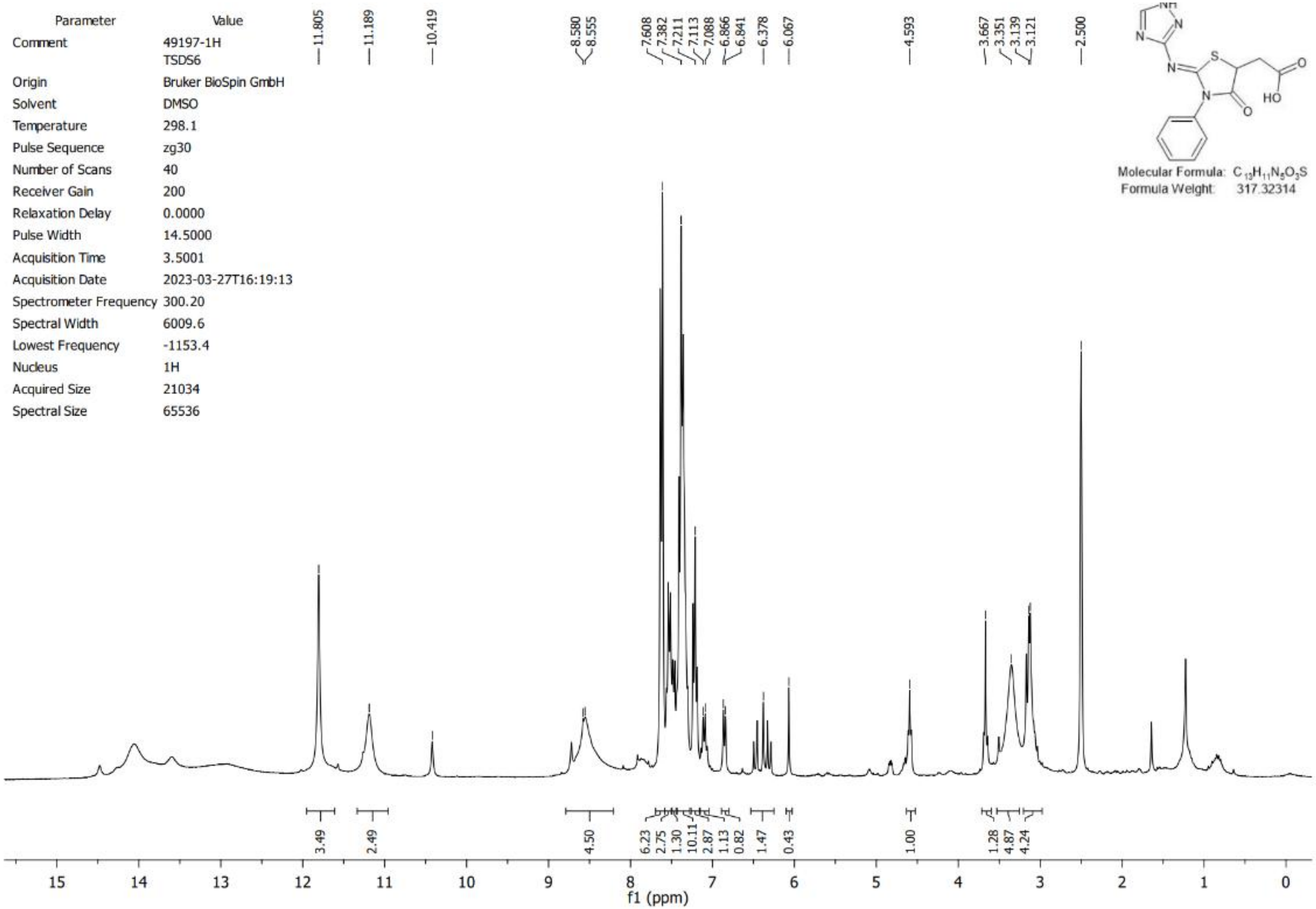

Figure S12. ^{13}C NMR of compound **6.**

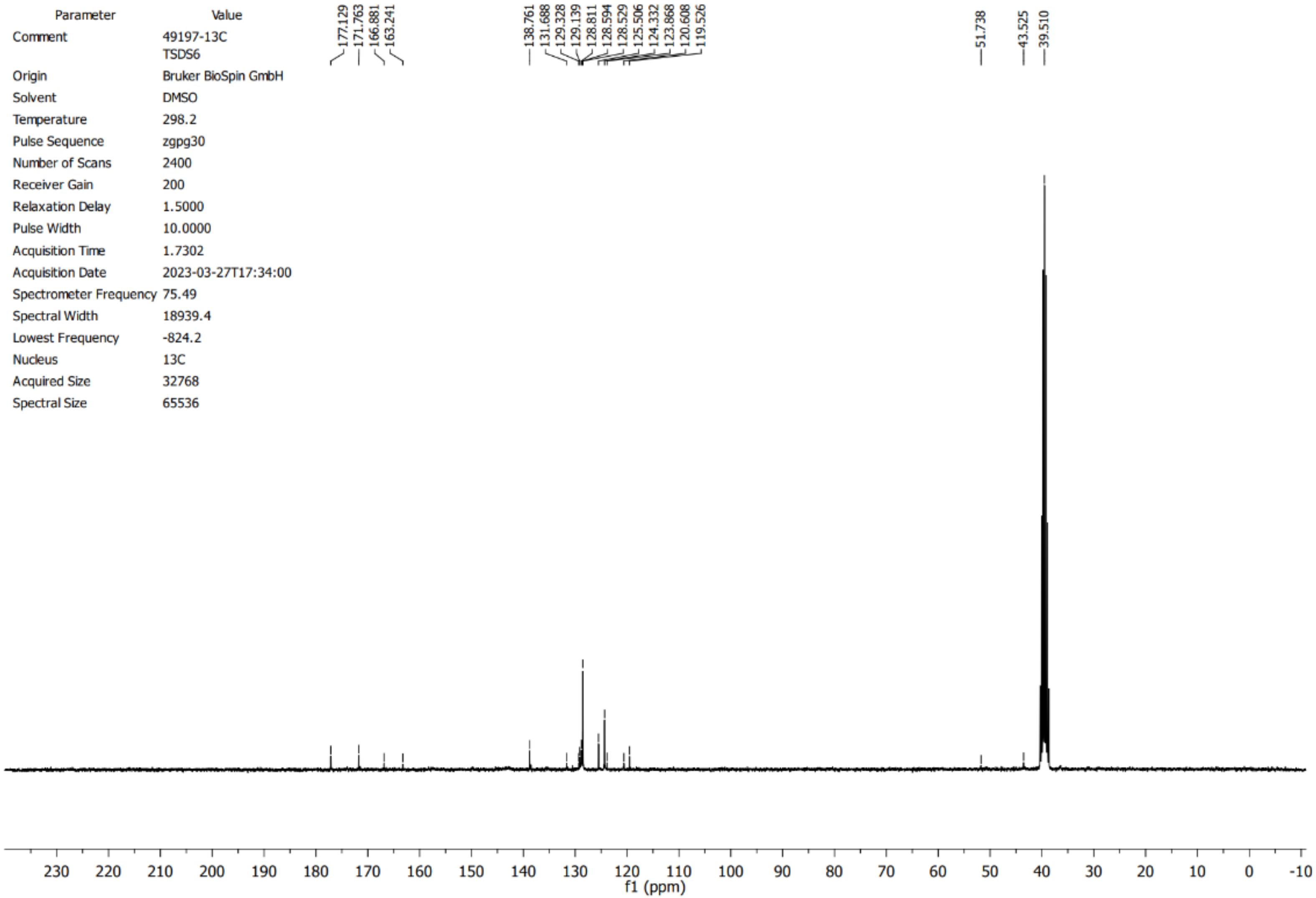

Figure S13. ^{1}H NMR of compound **7.**

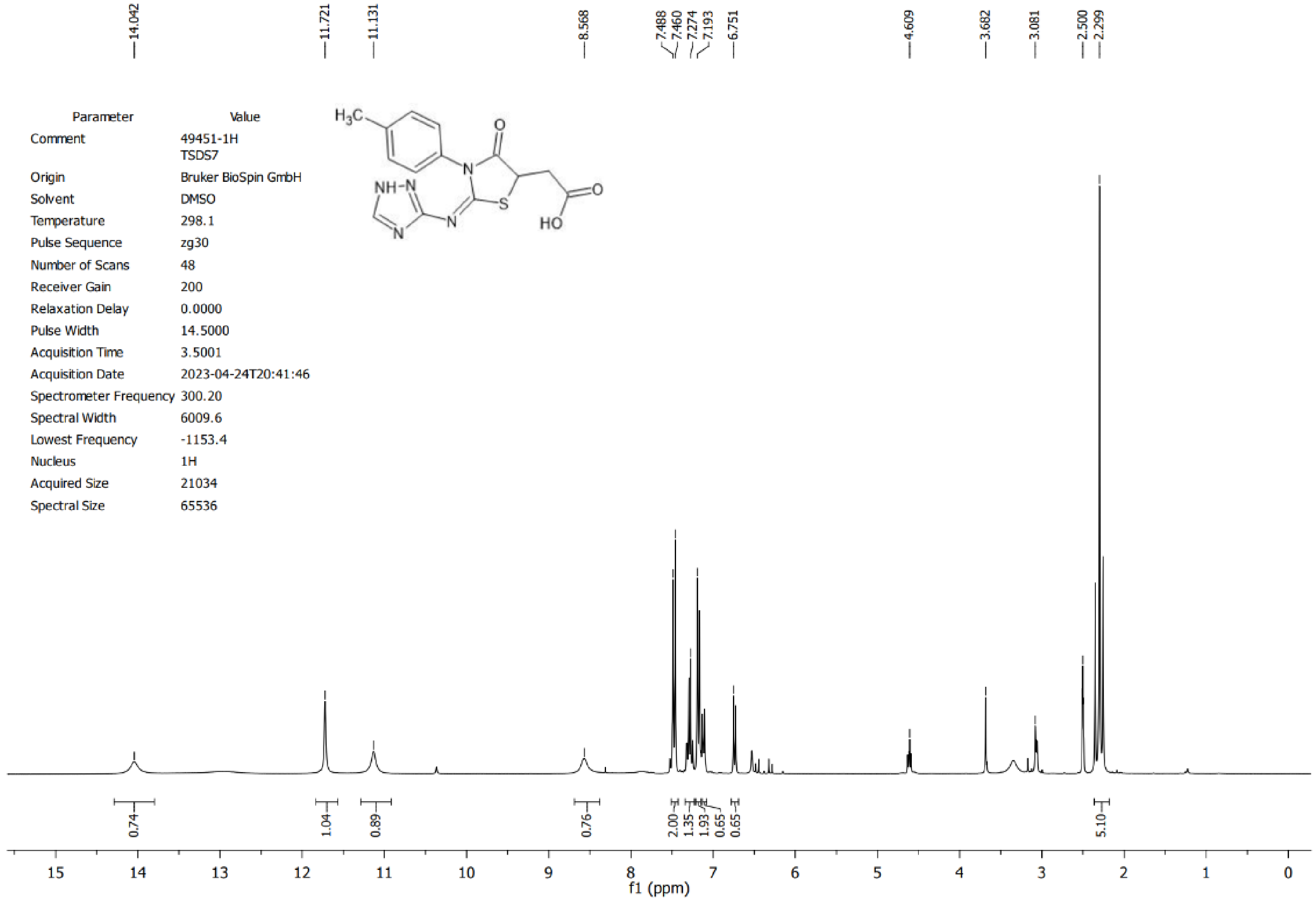

Figure S14. ^{13}C NMR of compound **7.**

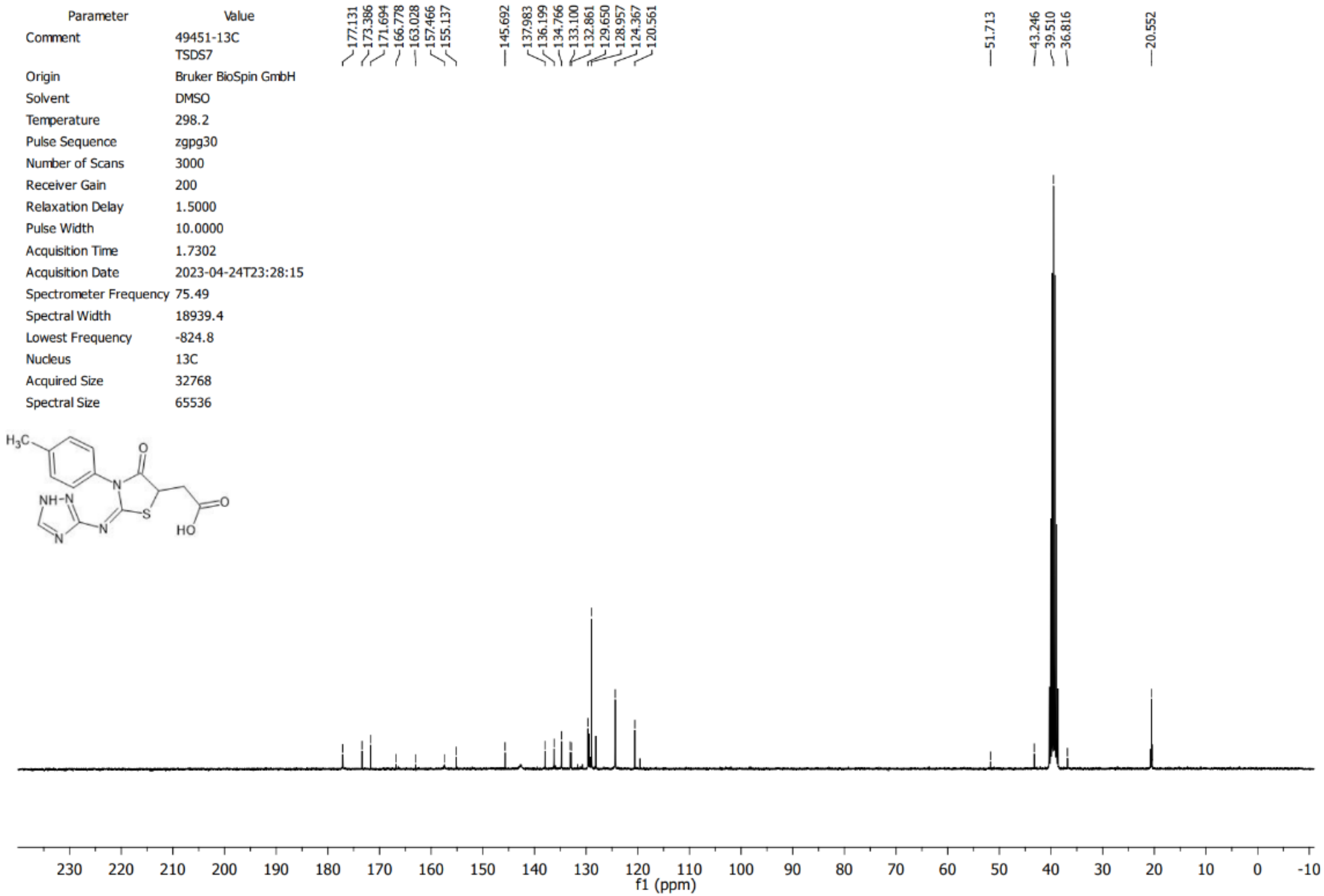

Figure S15. ^{1}H NMR of compound **8.**

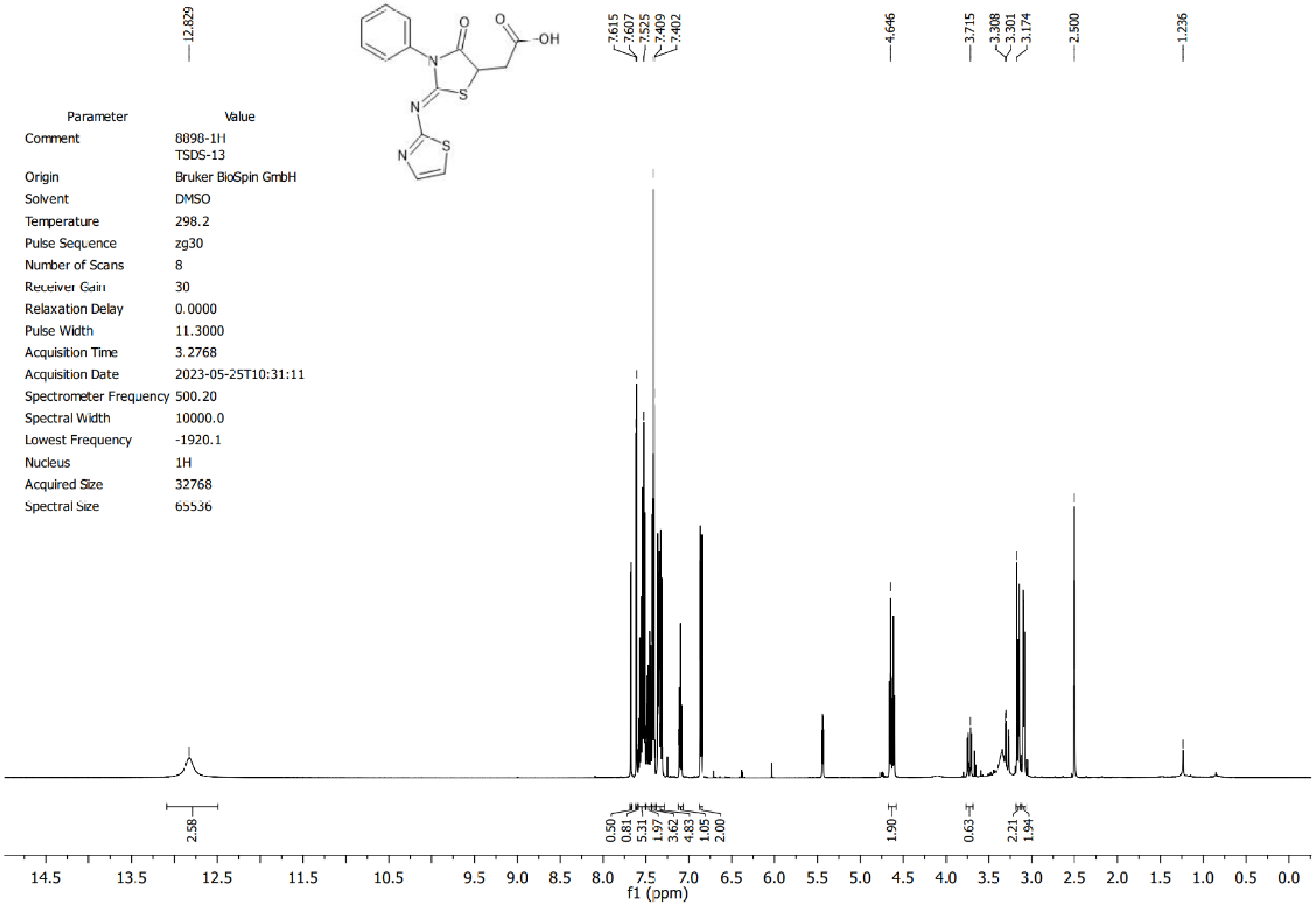

Figure S16. ^{13}C NMR of compound **8.**

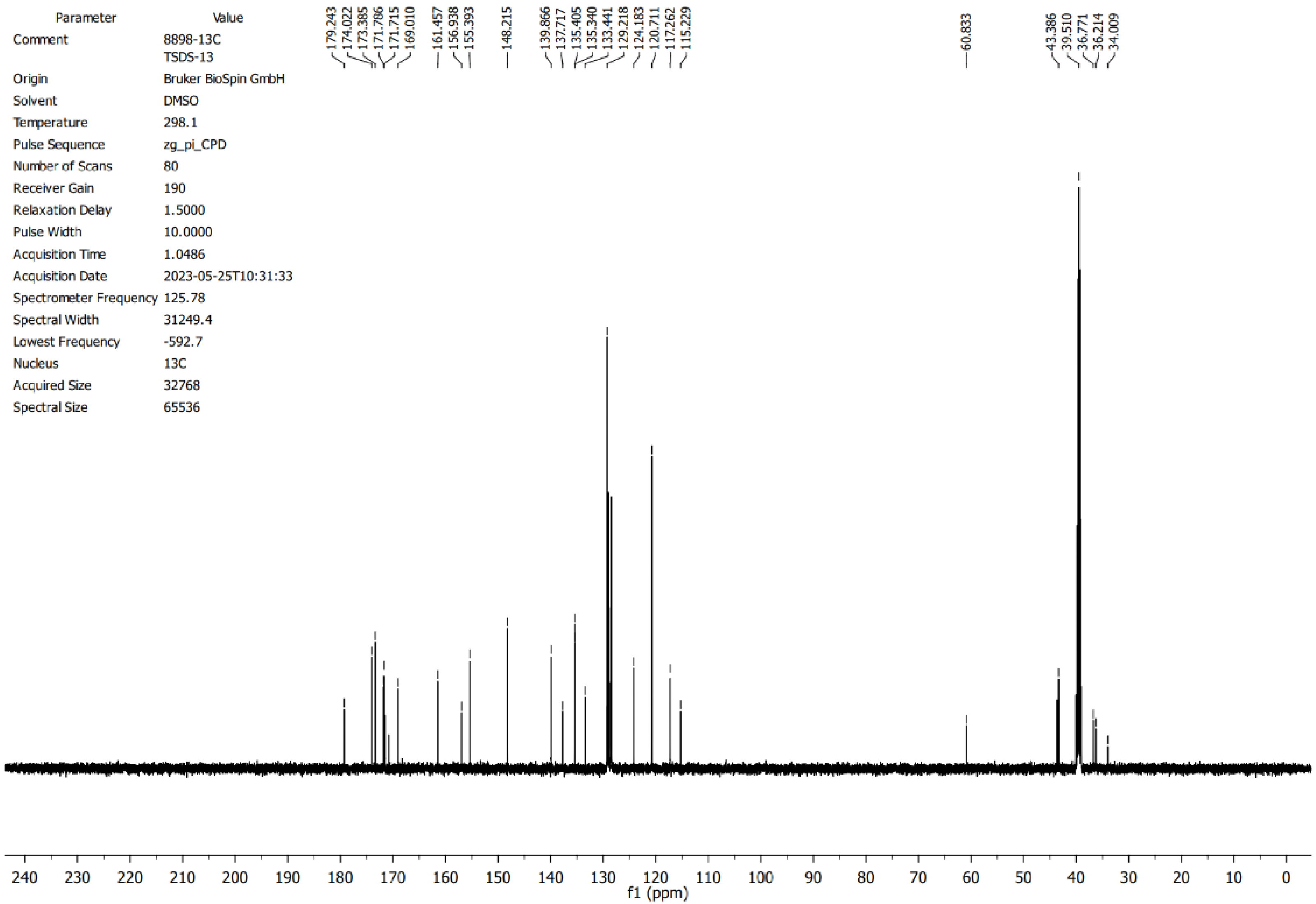

Figure S17. ^{1}H NMR of compound **9.**

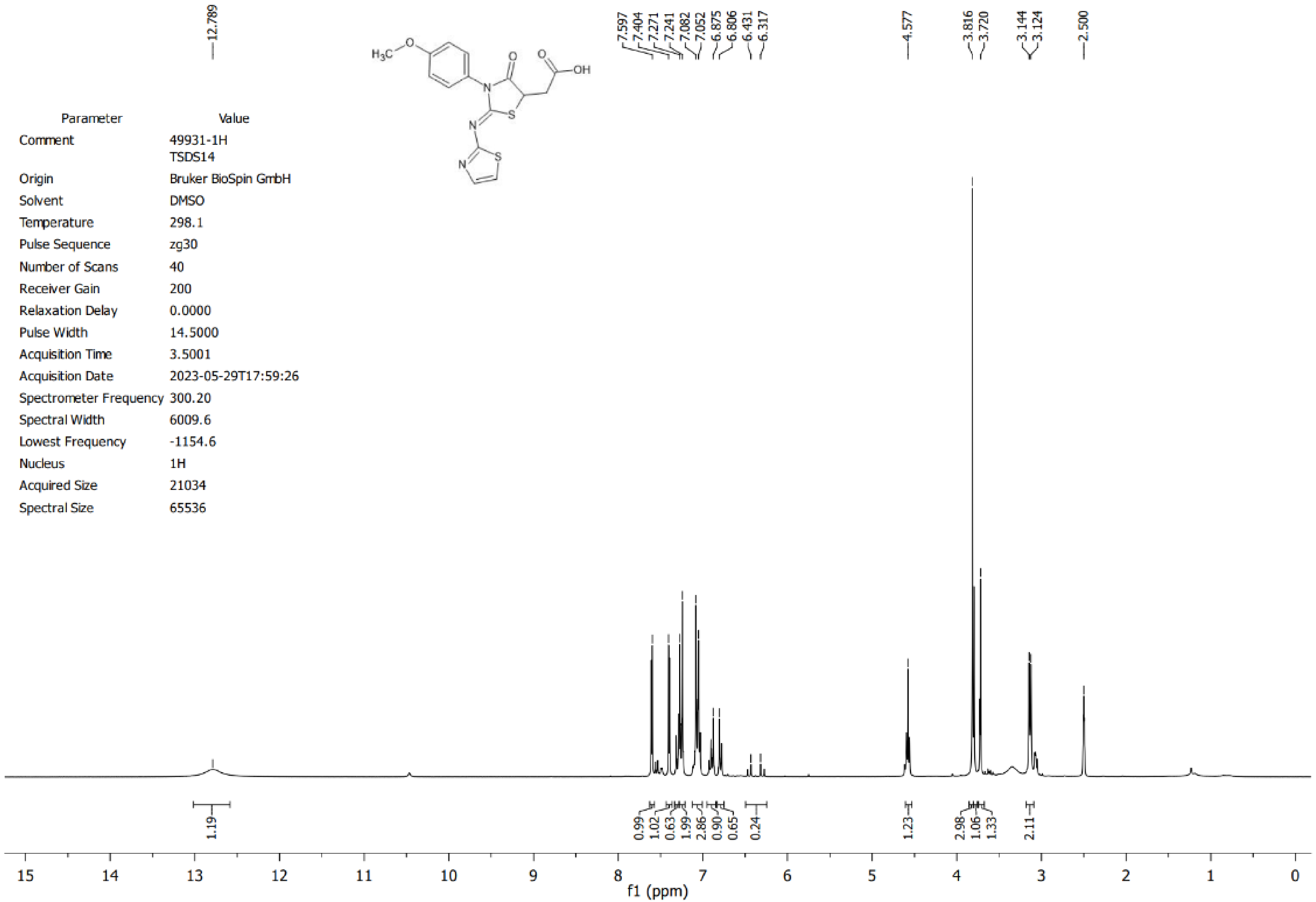

Figure S18. ^{13}C NMR of compound **9.**

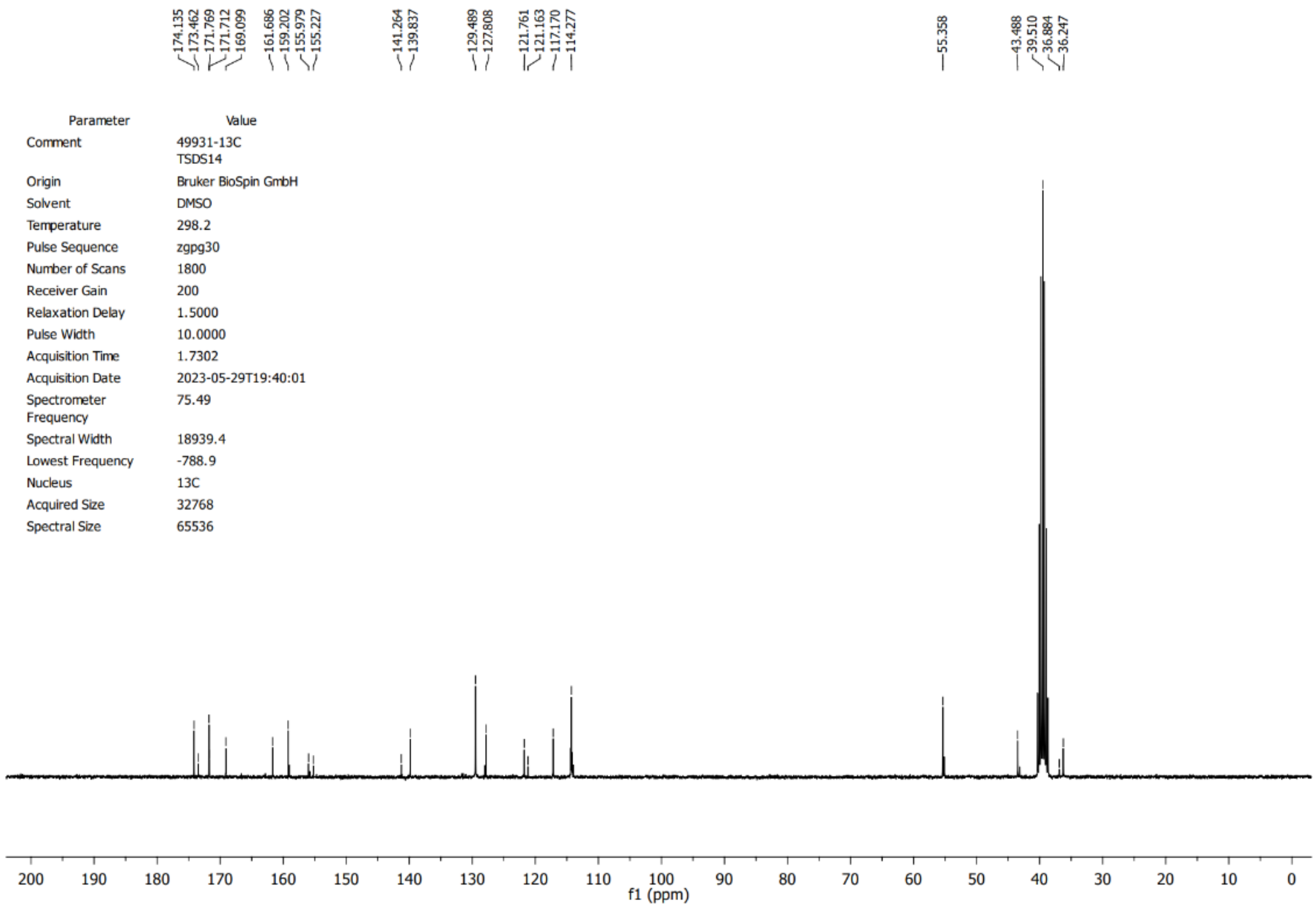

Figure S19. ^{1}H NMR of compound **10.**

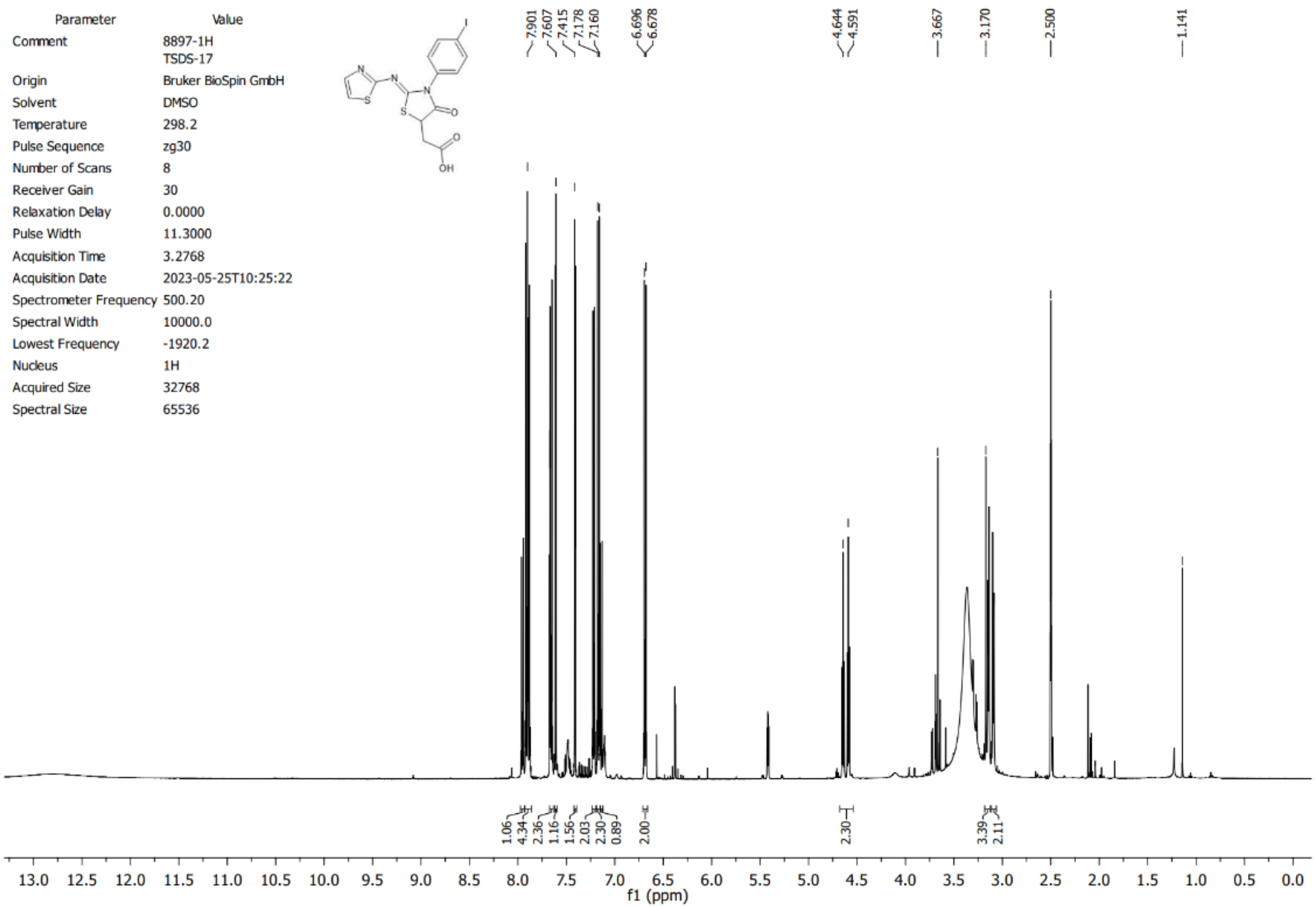

Figure S20. ^{13}C NMR of compound **10.**

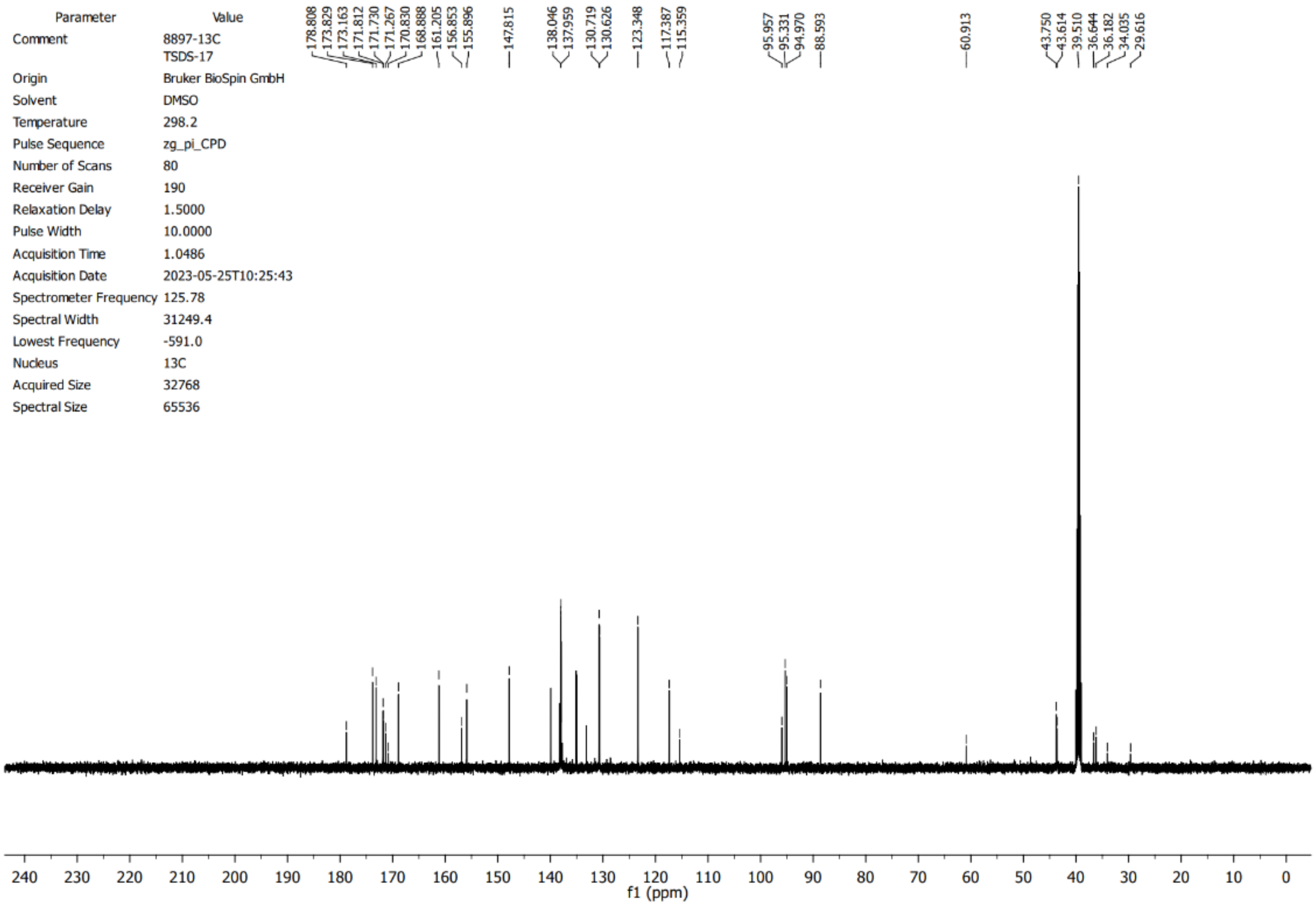

Figure S21. ^{1}H NMR of compound **11.**

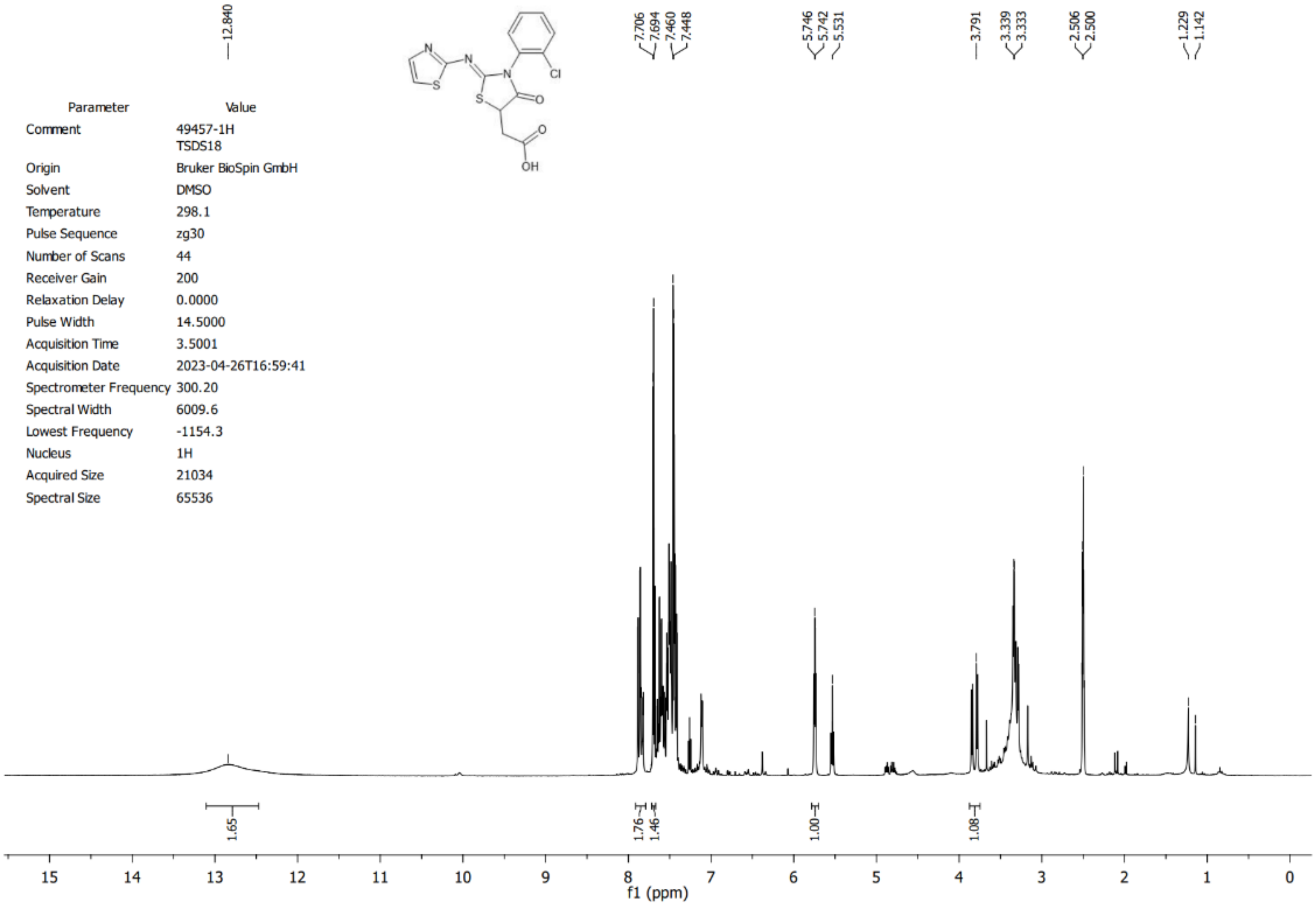

Figure S22. ^{13}C NMR of compound **11.**

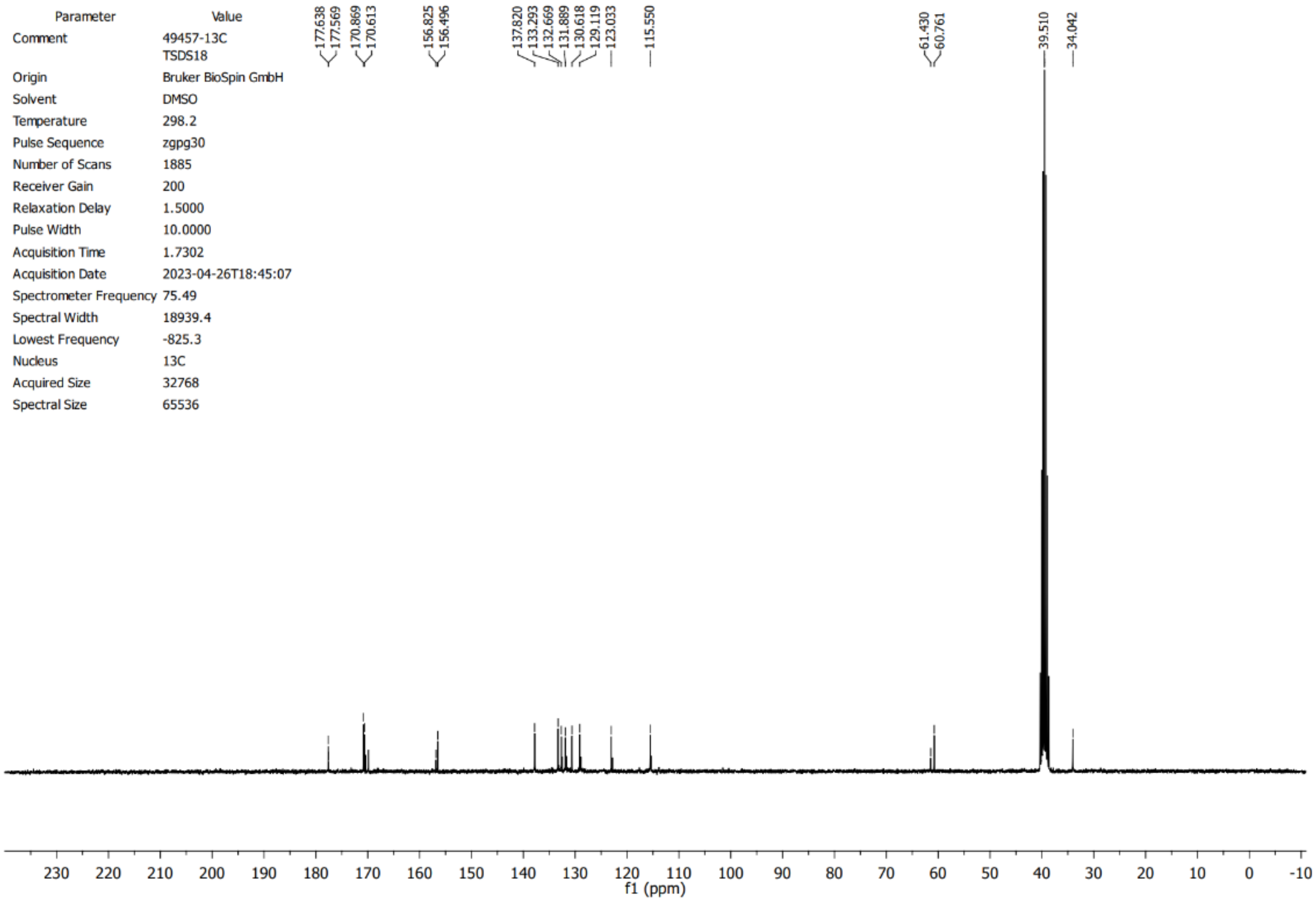

Figure S23. ^{1}H NMR of compound **12.**

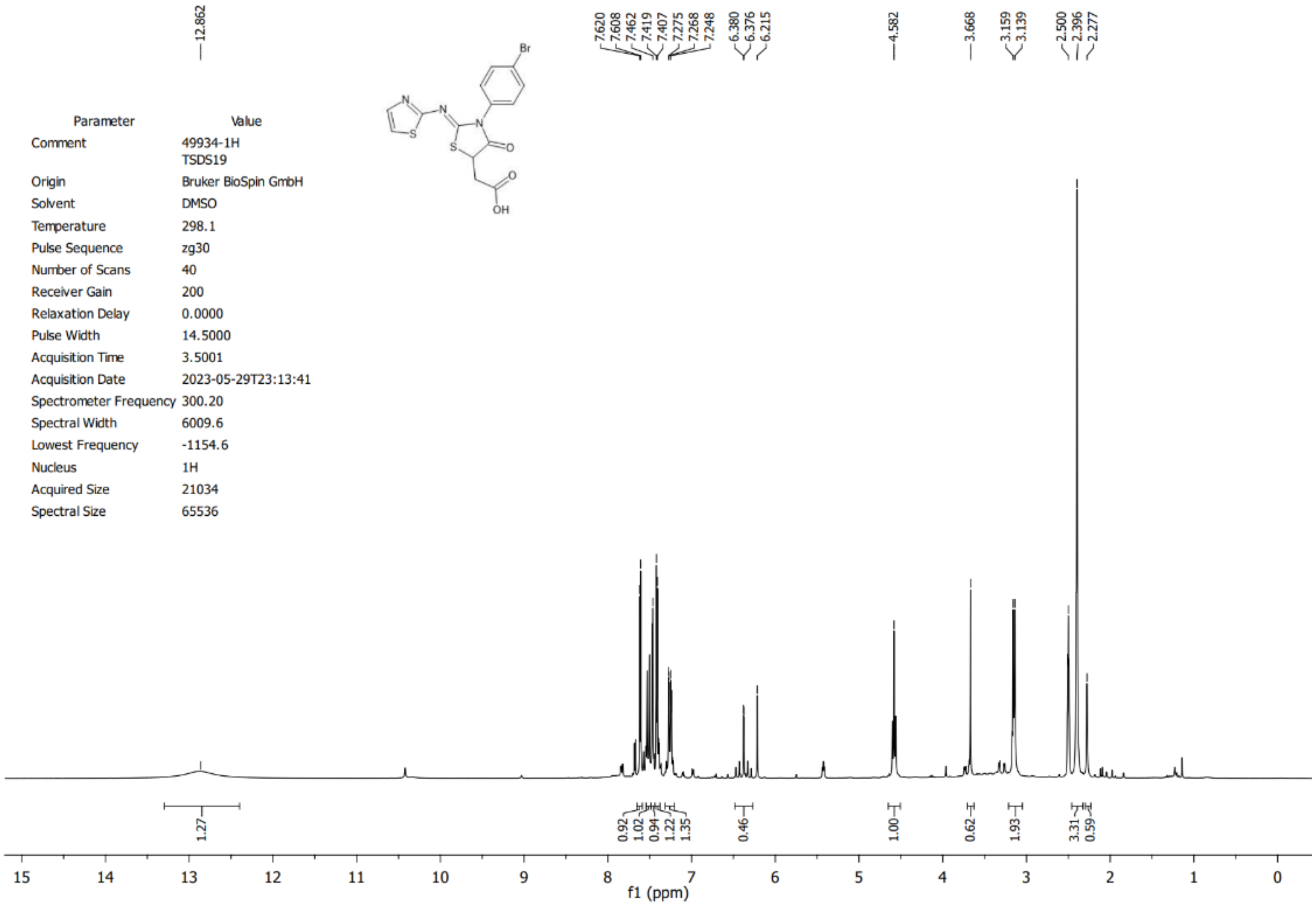

Figure S24. ^{13}C NMR of compound **12.**

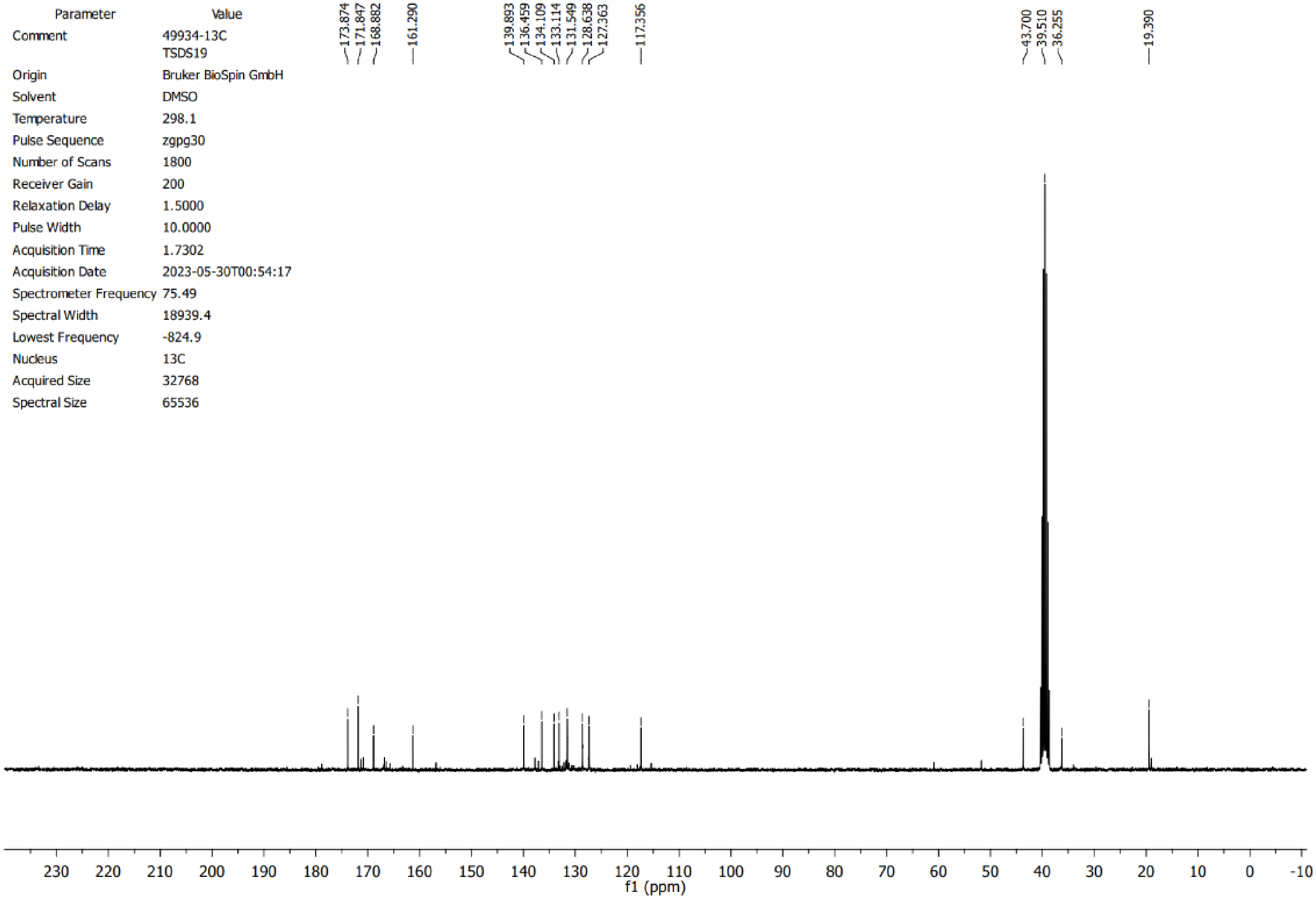

Figure S25. ^{1}H NMR of compound **13.**

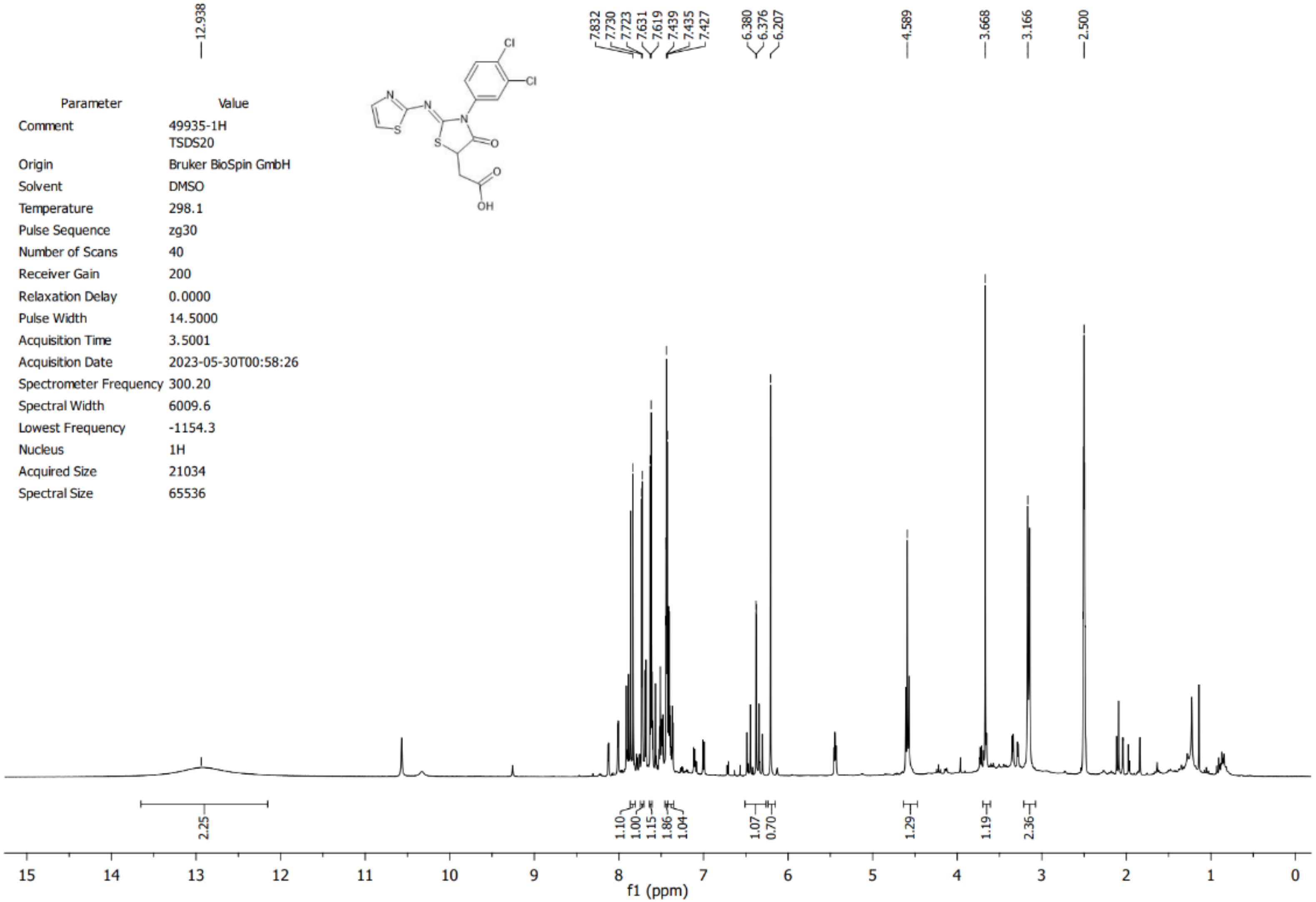

Figure S26. ^{13}C NMR of compound **13.**

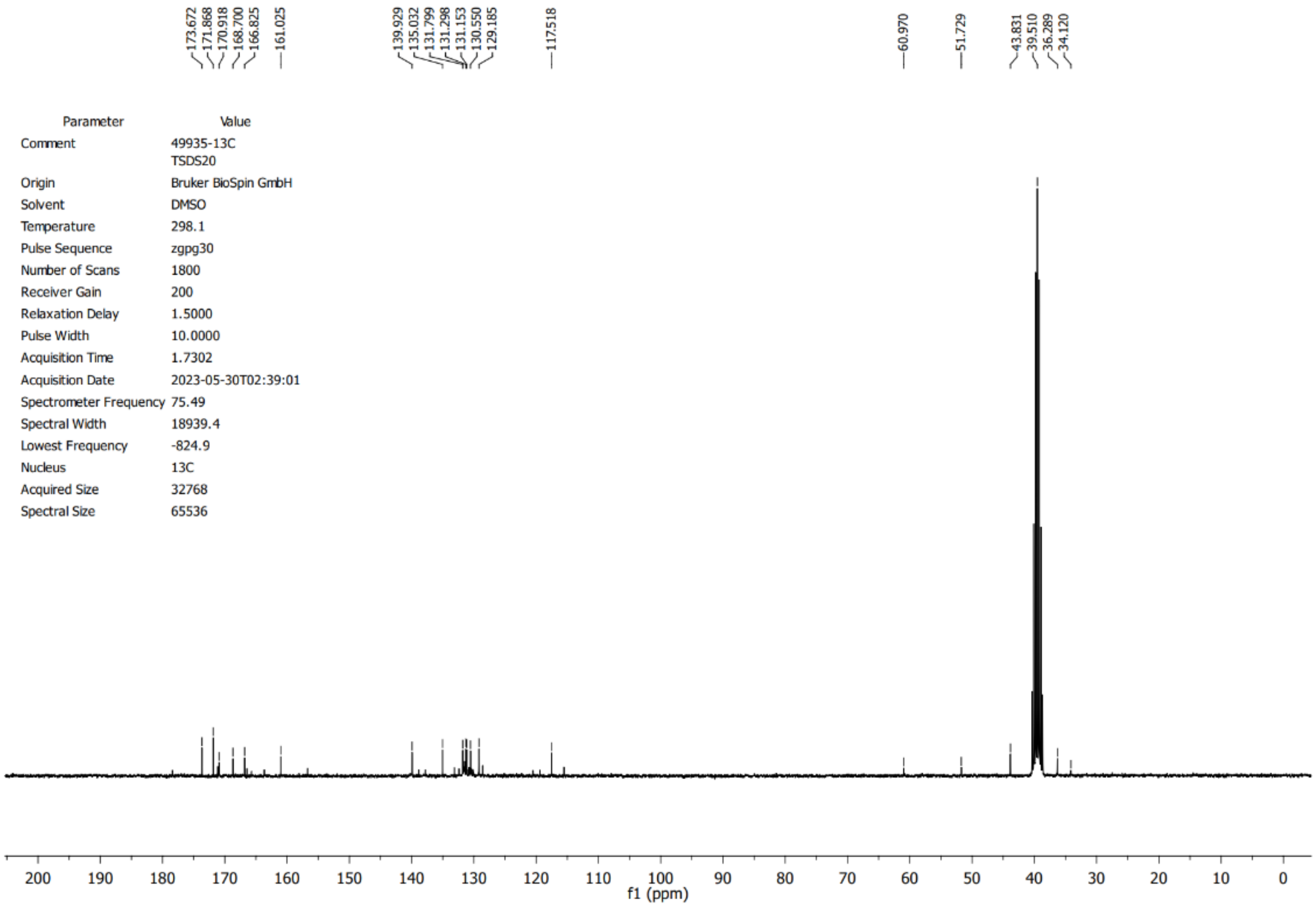

Figure S27. ^{1}H NMR of compound **14.**

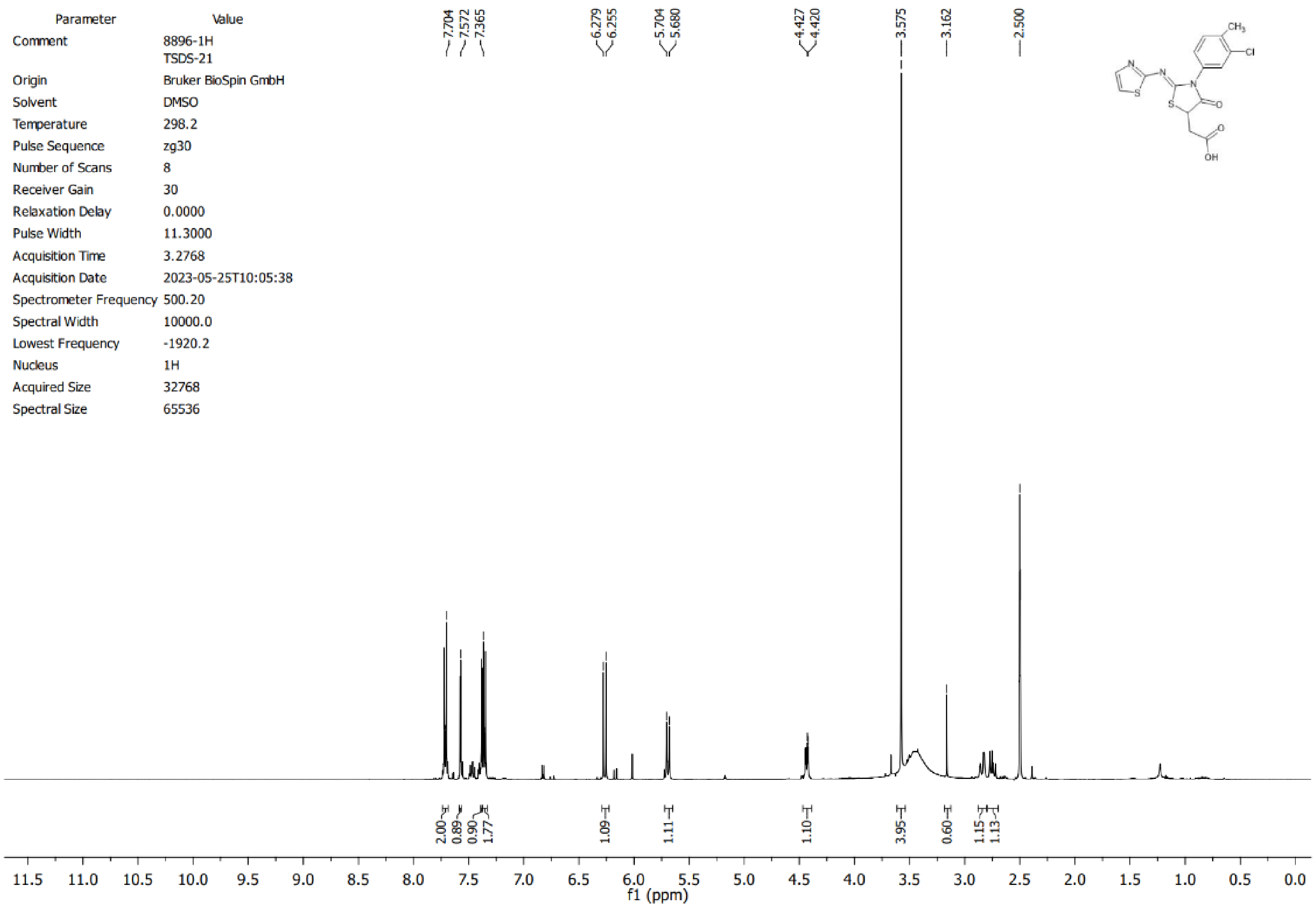

Figure S28. ^{13}C NMR of compound **14.**

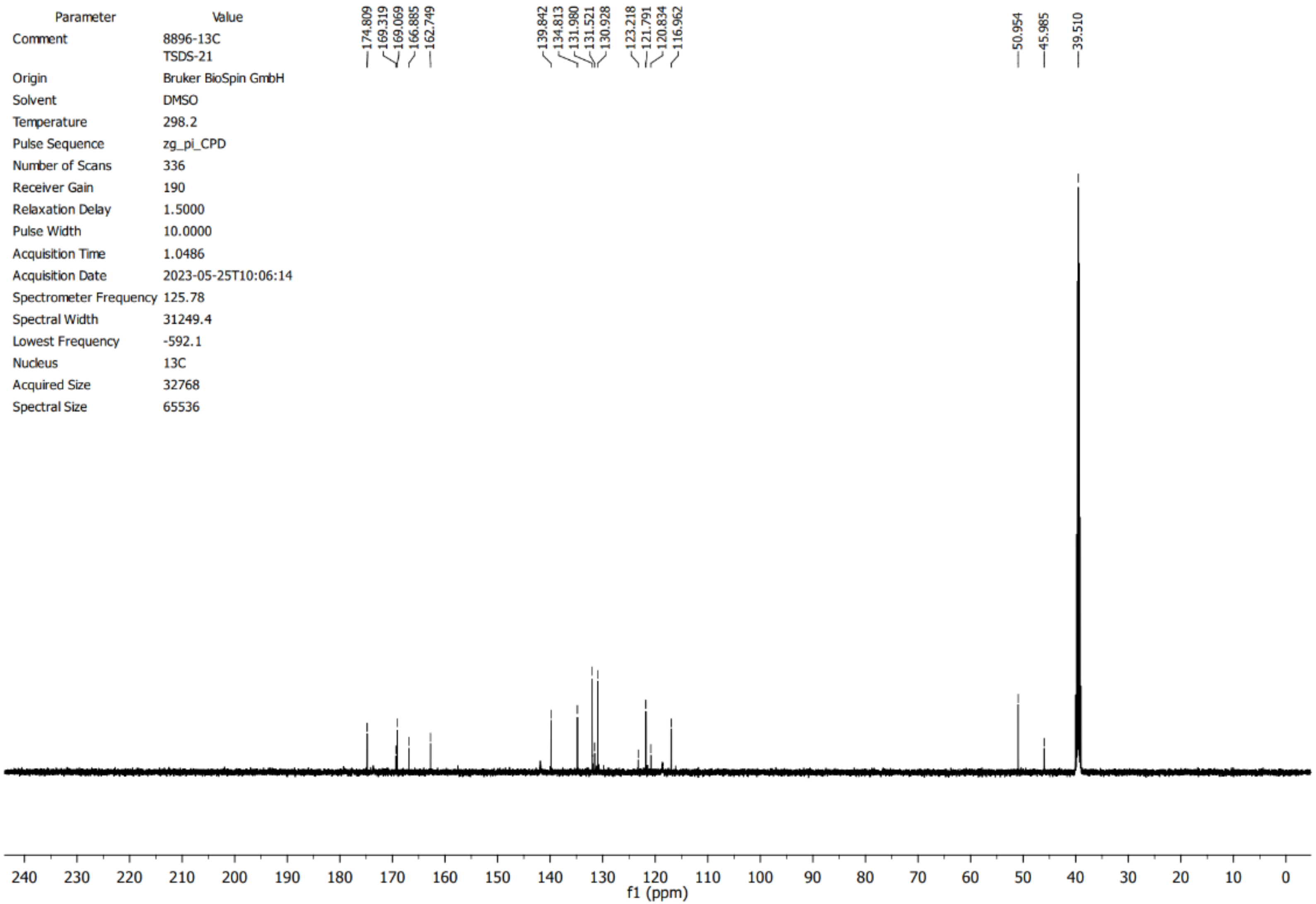

Figure S29. ^{1}H NMR of compound **15.**

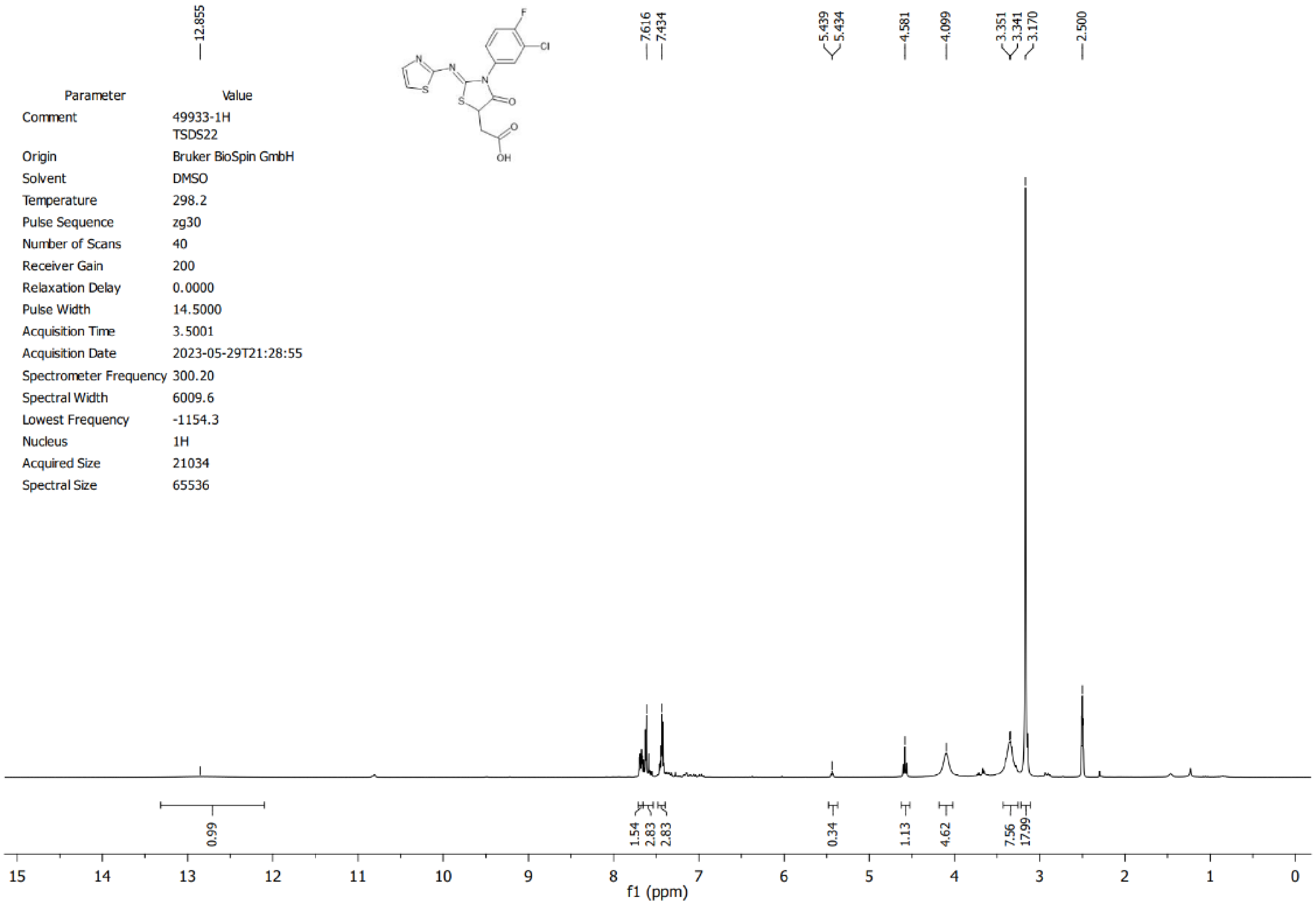

Figure S30. ^{13}C NMR of compound **15.**

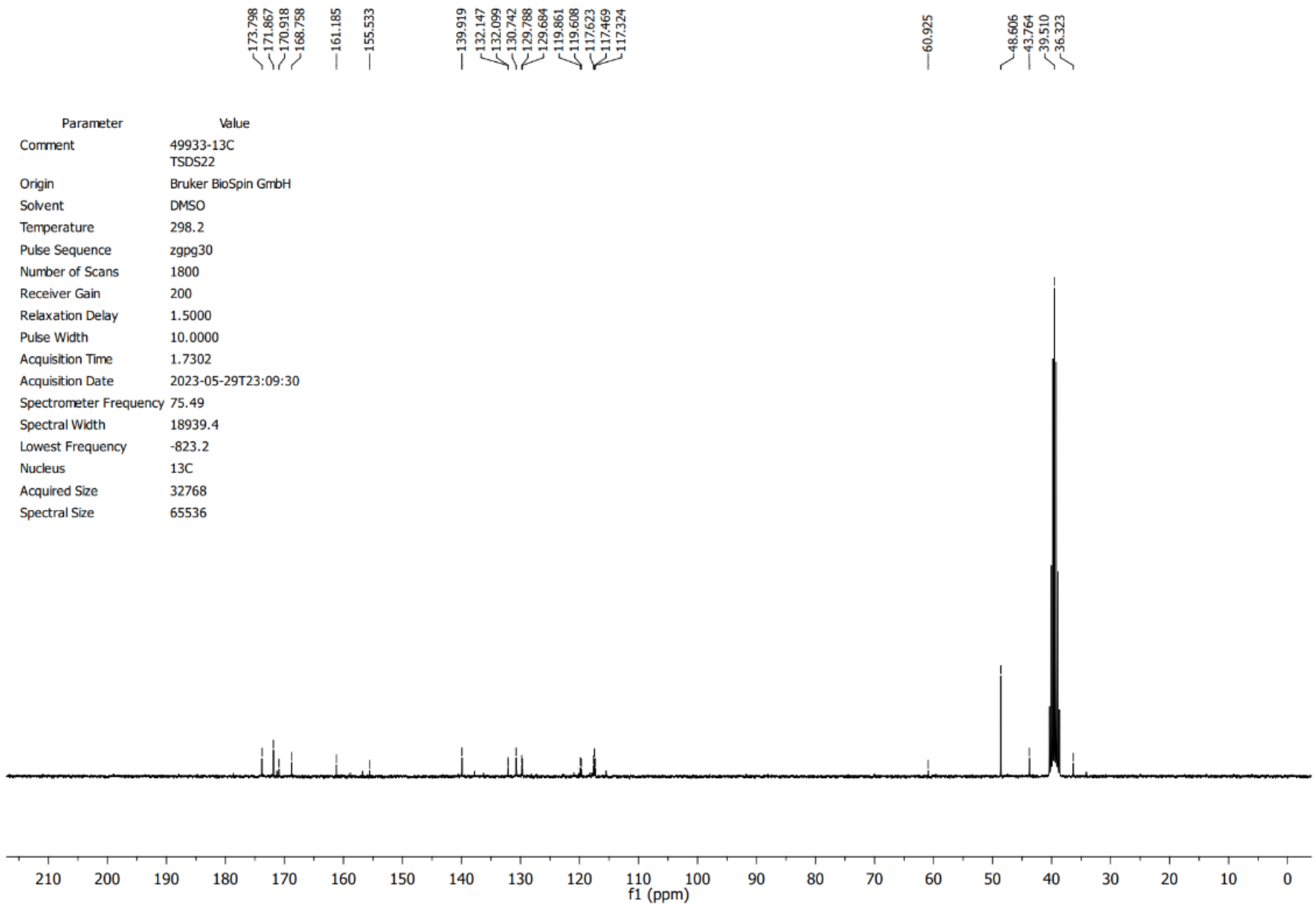

S.3.4.1 Molecular Dynamics Simulation

Trajectories for molecular dynamics are accessible through a reasonable request to the corresponding author. The file exceeded 30 GB of data and needs to be sent via data transfer.
